# Single-cell N^6^-methyladenosine regulator patterns guide intercellular communication of tumor microenvironment that contribute to colorectal cancer progression and immunotherapy

**DOI:** 10.1186/s12967-022-03395-7

**Published:** 2022-05-04

**Authors:** Yuzhen Gao, Hao Wang, Shipeng Chen, Rui An, Yadong Chu, Guoli Li, Yanzhong Wang, Xinyou Xie, Jun Zhang

**Affiliations:** 1 Department of Clinical Laboratory, Sir Run Run Shaw Hospital of Zhejiang University School of Medicine, Qingchun East Road, Jianggan District, Hangzhou, 310016 Zhejiang China; 2 Department of Gastroenterology, Second Affiliated Hospital of Zhejiang University School of Medicine, Hangzhou, 310009 Zhejiang China; 3 Department of Laboratory Medicine, Shanghai Eastern Hepatobiliary Surgery Hospital, Shanghai, 200438 China; 4 Institution of Gastroenterology, Zhejiang University, Hangzhou, 310009 Zhejiang China; 5 Key Laboratory of Precision Medicine in Diagnosis and Monitoring Research of Zhejiang Province, Hangzhou, 310013 Zhejiang China

**Keywords:** Single-cell, m^6^A, Tumor microenvironment, Colorectal cancer, Prognosis, Immunotherapy

## Abstract

**Background:**

N6-methyladenosine (m^6^A) RNA methylation plays a critical role in key genetic events for various cancers; yet, how m^6^A functions within the tumor microenvironment (TME) remains to be elucidated.

**Methods:**

A total of 65,362 single cells from single-cell RNA-seq data derived from 33 CRC tumor samples were analyzed by nonnegative matrix factorization (NMF) for 23 m^6^A RNA methylation regulators. CRC and Immunotherapy cohorts from public repository were used to determine the prognosis and immune response of TME clusters.

**Results:**

The fibroblasts, macrophages, T and B cells were respectively grouped into 4 to 5 subclusters and then classified according to various biological processes and different marker genes. Furthermore, it revealed that the m^6^A RNA methylation regulators might be significantly related to the clinical and biological features of CRC, as well as the pseudotime trajectories of main TME cell types. Bulk-seq analysis suggested that these m^6^A-mediated TME cell subclusters had significant prognostic value for CRC patients and distinguished immune response for patients who underwent ICB therapy, especially for the CAFs and macrophages. Notably, CellChat analysis revealed that RNA m^6^A methylation-associated cell subtypes of TME cells manifested diverse and extensive interaction with tumor epithelial cells. Further analysis showed that ligand-receptor pairs, including MIF −  (CD74 + CXCR4), MIF −  (CD74 + CD44), MDK–NCL and LGALS9 − CD45, etc. mediated the communication between m^6^A associated subtypes of TME cells and tumor epithelial cells.

**Conclusions:**

Taken together, our study firstly revealed the m^6^A methylation mediated intercellular communication of the tumor microenvironment in the regulation of tumor growth and antitumor immunomodulatory processes.

**Supplementary Information:**

The online version contains supplementary material available at 10.1186/s12967-022-03395-7.

## Introduction

Colorectal cancer (CRC) ranks third in incidence and second in mortality. Although tremendous efforts have been made to facilitate screening strategies, the prevalence of CRC has been increasing, and 1.9 million new cases were estimated in 2020. Among them, a large population is diagnosed at an advanced stage; nevertheless, the efficacy of current therapies for late-stage CRC is limited [1]. Thus, a deeper understanding of the molecular mechanisms of CRC might help to bring novel strategies to CRC prevention and therapy.

While DNA-related aberrations are widely studied in the literature, post-transcriptional alterations have been relatively less studied. Yet, these changes play a critical role in regulating the initiation and progression of CRC [2, 3]. Among these post-transcriptional alterations, N6-methyladenosine (m^6^A) is the most common internal modification in transcripts, comprising more than 50% of eukaryote methylations now identified as a new level of crucial epigenetic regulation for mRNA stability, splicing, and translation [4], as well as the generation of small and long non-coding RNAs [5]. Dynamic and reversible m^6^A methylation consists of readers (m^6^A-binding proteins), writers (methyltransferases), and erasers (demethylases) responsible for m^6^A's functions, methylation, and demethylation, respectively [6]. Importantly, m^6^A mRNA regulation plays a vital role in tumorigenesis, tumor development, and metastasis, and the dysregulation of m^6^A is closely correlated with the development and pathogenesis of CRC [7–9].

Nowadays, emerging evidence has proven the critical role of tumor microenvironment (TME) in the progress and metastasis of tumor. In addition, single-cell transcriptomics further revealed the complex intercellular communication between diverse subtypes of TME cells and tumor cells [10, 11]. The TME cells consists of multiple cell types in addition to tumor cells, such as cancer-associated fibroblasts (CAFs), tumor-associated macrophages (TAMs), T cells, and B cells. Recently, Huilong et al. discovered that ablation of Mettl3 in myeloid cells promotes tumor growth and metastasis in vivo [12]. Dali et al. reported that loss of YTHDF1 in dendritic cells can lead to enhanced cross-presentation of tumor antigens and the cross-priming of CD8^+^ T cells in vivo [13]. However, less research was performed to investigate the cell–cell interaction between m^6^A mRNA modification associated subtypes of TME cells with tumor cells.

Here, we investigated the influence of m^6^A mRNA methylation on the main TME cells, including stromal cells, myeloid cells, T cells, and B cells, based on 65,362 single-cell sequencing data derived from 33 CRC tumor samples [14]. By nonnegative matrix factorization (NMF) clusters of 23 m^6^A RNA methylation regulators, as described previously [15, 16], it was observed that different patterns of m^6^A mRNA methylation in each CRC TME cell type subpopulations manifested extensive and diversity communication with tumor epithelial cells, and associated with different immune characteristics, metabolic pathways, transcription characteristics and prognosis. To the best of our knowledge, the present study reveals, for the first time, that m^6^A mRNA may guide intercellular communication of TME cells with tumor cells to contribute to colorectal cancer progression based on our comprehensive single-cell analysis.

## Materials and methods

### Study design and data collection

Single-cell mRNA sequence (scRNA-seq) data from 23 CRC patients with 33 samples in the SMC dataset, 23 tumor tissues, and 10 normal adjacent tissues were collected to analyze the landscape of 23 m^6^A RNA methylation modification regulators. After preliminary sample integration, we generated a gene expression and phenotype matrix for 65,362 scRNA-seq datasets. Full data were downloaded from GSE132465 in the Gene Expression Omnibus (GEO) database (www.ncbi.nlm.nih.gov/geo). In addition, 11 public datasets of bulk mRNA sequence or microarray data for 2653 CRC patients were also obtained from The Cancer Genome Atlas (TCGA) and GEO databases (Additional file 1: Table S1). All data generated or analyzed during this study are freely available in previous publications or the public domain.

### Visualization of TME cell types and subtypes in CRC

Using the Seurat package in R software, we created Seurat objects for total and individual cell types belonging to the scRNA-seq gene expression matrix based on acquisition. Then, the top 2000 genes, selected as the top variable features, were used as the basis for normalizing the scRNA-seq data for each cell by using the FindVariableFeatures of the Seurat package. Furthermore, we performed ScaleData and RunPCA functions to obtain the number of principal components (PC) based on the Seurat objects. We used “t-SNE (t-distributed stochastic neighbor embedding)” dimensionality reduction to further summarize the top principal components. Finally, with the annotated information for each cell in CRC supported by the previous article, the Idents and DimPlot functions were used to annotate and visualize the cells of the major TME cell types or subtypes.

### Pseudotime trajectory analysis of m^6^A mRNA regulators for TME cells

To investigate the relationship of cell pseudotime trajectories with m^6^A regulators, we employed the Monocle R package for single-cell RNA data for all cell types in CRC [17]. Highly variable genes were set according to the following filtering criteria: mean expression  ≥  0.1 and dispersion_empirical ≥ 1* dispersion_fit. The DDRTree method was used for dimensionality reduction. Then we used the ‘plot_pseudotime_heatmap’ function to visualize heatmaps showing the dynamic expression of m^6^A regulators in the pseudotime trajectories of different TME cell types in CRC.

### Non-negative matrix factorization of m^6^A mRNA regulators in TME cells

To best observe the effect of m^6^A-mediated regulator expression on TME cell types, we carried out a dimension reduction analysis for 23 m^6^A regulators in all TME cell types, using the non-negative matrix factorization algorithm (NMF R package, version 0.20.6), and identified different cell subtypes for these cell types, depending on the scRNA expression matrix. All these steps were performed in a manner similar to the previous studies [10, 18].

### Identification of the marker genes of m^6^A-related cell subtypes in TME cells

We used the FindAllMarkers function to list the markers of each NMF cluster of each cell type in CRC. The parameters for min.pct and logfc.threshold were set as 0.15, and then we filtered the genes by using the adjusted p value < 0.05 for further research. The Dotplot function was performed to show the top highest gene expressions in each NMF cluster. The AddModuleScore function calculated the signature scores based on differentially expressed genes (DEGs) among these NMF cell clusters. The FeaturePlot function was used to show the distribution of specific signatures of NMF cluster scores in the TME of CRC. The special gene sets used in the comparisons among m^6^A-related clusters were listed in Additional file 1: **Table S2**.

### Functional Enrichment Analysis for NMF m^6^A-related subtypes

Based on these marker genes among NMF clusters in different TME cell types, the clusterProfiler R package was used to detect Kyoto Encyclopedia of Genes and Genomes (KEGG) and the Reactome pathway database. Ctyoscape enrichment map function was used to cluster the pathways. Only gene sets with adjusted p-value < 0.05 were considered significantly enriched. In addition, 50 hallmark signatures and 113 metabolic pathways were collected from the molecular signature database (MSigDB) and a previous study [19], followed by performing gene set variation analysis (GSVA) to calculate the enrichment scores for these NMF clusters.

### SCENIC analysis for NMF m^6^A-related subtypes

The pySCENIC package (version 0.9.0), a Python-based implementation of the SCENIC pipeline was used to investigate the gene regulatory network of transcription factors (TFs) in CRC [20]. Two gene-motif rankings (hg19-tss-centered-10 kb and hg19-500 bp-upstream) from the RcisTarget database were used to detect the transcription start site (TSS) and the gene regulatory networks in the scRNA-seq data in CRC. TFs with adjusted by Benjamini–Hochberg false discovery rate (BH-FDR) < 0.05 were considered for further research.

### Cell–cell communication analysis for NMF m^6^A-related subtypes

CellChat, an R package described previously, contains ligand-receptor interaction databases for human and mouse that can analyze the intercellular communication networks from scRNA-seq data annotated as different cell clusters [21]. First, we used CellChat to evaluate the major signaling inputs and outputs among all NMF TME cell clusters using CellChatDB.human. Then, we used the netVisual_circle function to show the strength or weakness of cell–cell communication networks from the target cell cluster to different cell clusters in all NMF clusters. Finally, the netVisual_bubble function shows the bubble plots of significant ligand-receptor interactions between the target cell cluster and other TME NMF clusters.

### Survival analyses with m^6^A-related signatures in public bulk RNA-sequence datasets

Based on the FindAllmarker function in the Seurat R package, we generated m^6^A-related gene signatures for all NMF cell clusters. Also, the main cell type of CRC TME were also calculated based on the scRNA data. Then the GSVA function was used to calculate these gene signature scores in all 12 public datasets of CRC. The log-rank test and Cox proportional hazard regression were conducted to explore the relationship between m^6^A-related NMF signatures and patients’ prognosis, including overall survival (OS) rate and recurrence-free survival (RFS) rate. The cutoff values of different NMF cell signatures in the different public datasets were determined by the survminer R package used to plot Kaplan–Meier curves. To obtain the prognosis of NMF m^6^A-related signatures, we used the RMA function from the metafor R package to pool the Cox-regression results of the same signatures from all available public datasets. Finally, the forestplot R package was used to show meta-analysis results.

### Collection of immunotherapy transcriptomic

12 Immune checkpoint blockade immunotherapeutic (ICB) cohorts with FPKM or CPM transcriptomic were collected from the public database, included 8 melanoma datasets (Ulloa et al. (2013, MAGE A3,Melanoma [22]);Gide et al. (2019, anti-PD1 or anti-PD1 + CTLA4, Melanoma [23]); Nathanson (2017 CTLA4, Melanoma [24]); Hugo et al. (2016, anti-PD1, Met Melanoma [25]); Lauss et al. (2017, ACT, Melanoma [26]); Liu et al. (2019, anti-PD1, Met Melanoma [27]); Riaz et al. (2017, anti-PD1, Melanoma [28]); VanAllen (2016, CTLA4, MetMelanoma [29])), and other 4 non-melanoma cohorts (IMvigor210 (2018, anti-PDL1,Urothelial Cancer [30]); Braun et al. (2020, anti-PD1, CCRCC [31]); JaeWon et al. (2020, anti-PD1, NSCLC [32]);and Rose et al. (2021, ICB, Bladder Cancer [33])). All patients had the immune response in these cohorts.

### Statistical analysis

Standard tests included Student’s *t*-test, Wilcoxon rank-sum test, Kruskal–Wallis test, and Chi-square test for the differences of continuous target or category variables in these cell subgroups. Pearson analysis was used to show the correlation of different cell signatures or gene expressions among TME CRC cell types. To compare the biological features of each m^6^A-related subtype for each TME cell type in CRC, we collected many current CRC-related gene signatures or TME cell function-related gene lists from the previous public publication. Then, we used the ComplexHeatmap or pheatmap packages to visualize the different expressions of the scale data of target variables among the NMF clusters from the TME CRC cell types. Routine statistical analyses of the present study were performed in R 4.0 software, and a two-sided p-value below 0.05 was considered statistically significant.

## Results

### The landscape of m^6^A regulators in TME cells in CRC

**Fig. 1:**
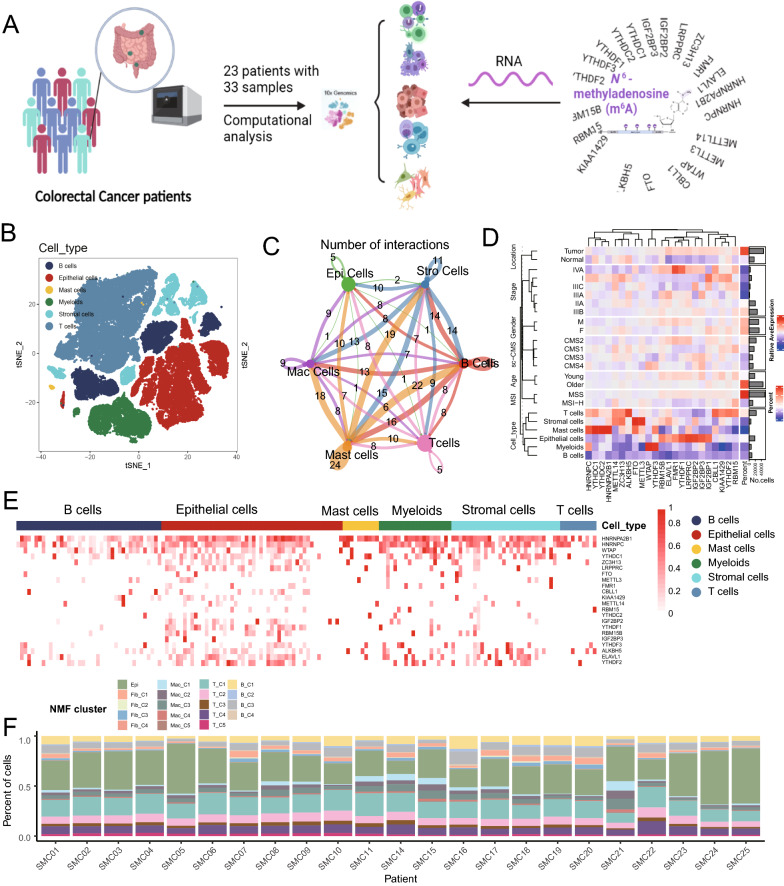
Overview of m^6^A RNA methylation regulators in the single-cell data for colorectal cancer. **A** The overall design of the present study and the data sourced from the SMC dataset (GSE132465). **B** Cell type annotations by using the Seurat t-distributed stochastic neighbor embedding (t-SNE) plot of 65,362 cells; **C** Cell–Cell communications between main six cell types by Cell chat analysis. **D** Average expression of m6A RNA methylation regulators in 65,362 cells according to different clinical variables in the SMC dataset containing cell types, including class types (normal vs. tumor), MSI (MSI-H vs. MSS), Age (old vs. young), Stage (I, IIA, IIIA, IIIB IIIC, and IVA), and Gender (female *vs*. male) by using z-score. **E** Heatmap distribution of m^6^A RNA methylation regulators in B cells, epithelial cells, mast cells, myeloid cells, stromal cells, and T cells. **F** NMF cluster by using the 23 m6A regulators RNA expression respectively for the main four types of TME cells (CAFs, Macrophage cells, T cells, and B cells) in the scRNA data

Overall, the scRNA-seq dataset of CRC, as described previously, was used to explore the landscape of m^6^A RNA methylation regulators (Fig. 1A). The SMC dataset contained 65,362 TME cells annotated with major cell types, including epithelial cells, mast cells, myeloid cells, stromal cells, T cells, and B cells, in 33 samples from 23 CRC patients (Fig. 1B). Cell-chat analysis showed diverse and distinct interactions among these cell types (Fig. 1C). Here, we then fully evaluated the significant different expression between the mean RNA expression of m^6^A regulators and common variables of the CRC samples by using the AverageExpression function of Seurat, such as class type (normal *vs*. tumor), MSI status (MSI-H *vs*. MSS), age group (older > 60 vs. young <  = 60), AJCC stage (I, IIA, IIIA, IIIB IIIC, and IVA) and gender (female *vs*. male) (Fig. 1D). Obviously, the expression of m^6^A regulators was indeed different among six cell types in CRC from the SMC dataset (Fig. 1E). The heatmap also shows the differential expression of m^6^A regulators in 33 CRC samples with significantly different percentages of major cell types (Additional file 2: Figure S1A). Furthermore, to assess the relationship between m^6^A regulators and immunologic state in TME cells, we also estimated the ImmuneScore using the “estimate” R package [34] for 65,362 TME cells of CRC. Among six TME cell types, we found different strong associations of writer and eraser regulators using ImmuneScore (Additional file 2: Figure S1B).

Lastly, Fig. 1F showed the proportions of the m^6^A special NMF clusters for the interested four cell types (Fibroblasts, Macrophages, T cells, and B cells) by using the expression of 23 m^6^A regulators in scRNA data, respectively. All of their marker genes of these m^6^A related clusters were listed in the Additional file 1: Table S2 in all special TME cells.

### Novel m^6^A-mediated fibroblasts contributed to the TME of CRC

**Fig. 2:**
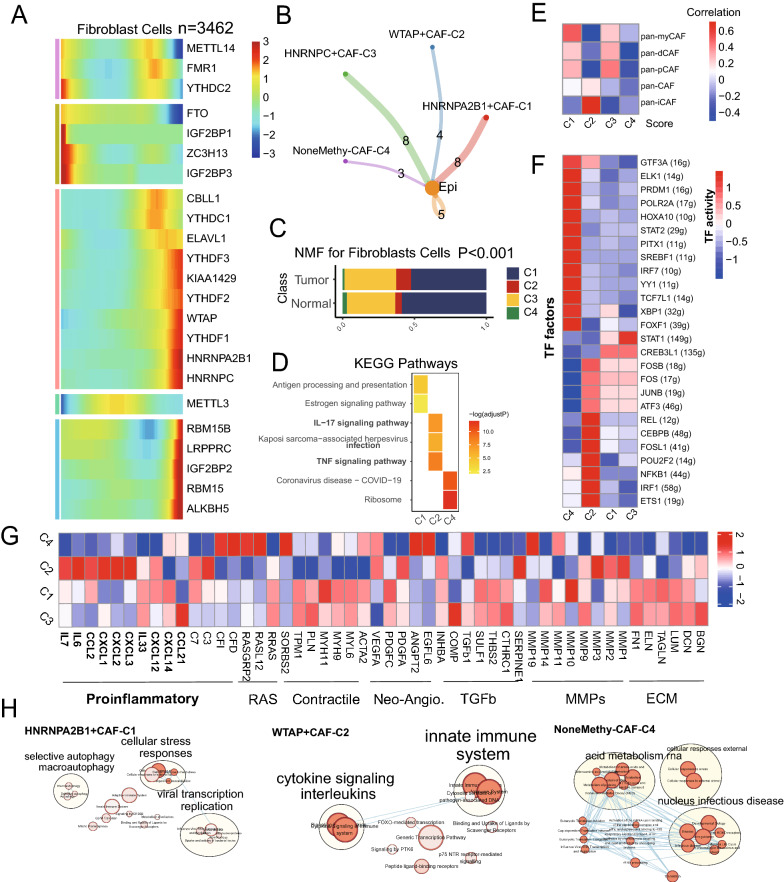
m^6^A regulators modified the features of fibroblast cells. **A** Trajectory Analysis reveals the role of m6A genes in for fibroblast cells (3462 cells). **B** Cell–Cell communications from m6A-related fibroblast cells to epithelial cells. **C** Bar plot for four m^6^A-fib-clusters, along with source class, reveals that the percentage of m^6^A-fib-C2 in tumor is higher than that in normal mucosa (p < 0.001). **D** Heatmap showing the activated KEGG pathway in main m^6^A-fib-clusters by using the DEGs among these groups (p < 0.05). **E** Different m6A-related fibroblast clusters were correlated with the previous signatures (p < 0.05). **F** Heatmap showing the significantly different activities of TFs among four m^6^A fibroblast cell clusters by comparing the average AUC using pySCENIC in Python software (Kruskal–Wallis test, p < 0.001). TF activity is scored using AUCell. **G** Heatmap showing the different average expression of common signaling pathway genes in the four m^6^A-fib-clusters, including collagens, ECM, MMPs, TGFb, Neo-Angio, Contractile, RAS and Proinflammatory. **H** Enrichment cluster analysis for activated signaling ways and functions of m6A-related fibroblast types in the Cytoscape by the REAC database

Stromal cells in the CRC dataset could be grouped into fibroblast and non-fibroblast cells in both tumor and normal tissues of CRC (Additional file 2: Figure S2A, B). By combining TCGA-COAD and TCGA-READ into one dataset, we used the xCell algorithm to calculate tumor infiltration of fibroblast cells, and their high abundance showed a poor prognosis for CRC patients (Additional file 2: Figure S2C, p = 0.014). Also, the pseudotime analysis revealed that the m^6^A RNA regulators had a critical role in the trajectory process of TME cells including fibroblasts, NK cells, macrophages, CD4 + T cells, and CD8 + T cells, etc. (Fig. 2A, and Additional file 2: Figures S2D, S3). Thus, by the cell-chat analysis, we found that there were different number of ligand-receptor links between these m^6^A-related fibroblast clusters (named as HNRNPA2B1 + CAF-C1 (n = 1939), WTAP + CAF-C2 (n = 245), HNRNPC + CAF-C3 (n = 1194), and NoneMethy-CAF-C4 (n = 84)) and epithelial cells (Fig. 2B and Additional file 2: Figure S2E). Among them, the WTAP + CAF-C2 population had a higher percentage in tumor samples (n = 1501) than that in normal samples (n = 1961, Chi-square test p < 0.001) (Fig. 2C). Enrichment analysis with KEGG showed that the WTAP + CAF-C2 cell cluster exhibited IL-17 activity, TNF signaling, and neutrophil-related functions based on DEGs (Additional file 1: Table S3). Pan-CAF signatures, originated from a previous study [35], were also calculated, and we found that the WTAP + CAF-C2 score was strongly associated with inflammatory CAF (pan-iCAF) (Fig. 2E).

Next, gene regulatory network analysis showed the expression of 26 TFs was significantly different among the four clusters. It is noteworthy that TFs of REL, CEBPB, FOSL1, POU2F2, NFKB1, IRF1, and ETS1 were upregulated in the WTAP + CAF-C2 cluster (Fig. 2F). We additionally compared the expression of some surface protein genes among the four m^6^A-mediated CAF NMF clusters and found that CD34, LRRN3, BAMB1, P2RY6, CLDN1, and ICAM1 were higher in the WTAP + CAF-C2 (Additional file 2: Figure S2F). From the pathway heatmap (Fig. 2G), these the WTAP + CAF-C2 and NoneMethy-CAF-C4 had significantly different expressions of these pathway genes. Lastly, the enrichment map revealed that the HNRNPA2B1 + CAF-C1, WTAP + CAF-C2, and NoneMethy-CAF-C4 had different REACTOME pathway features (Fig. 2H).

### m^6^A-mediated macrophages resembled classical features

**Fig. 3:**
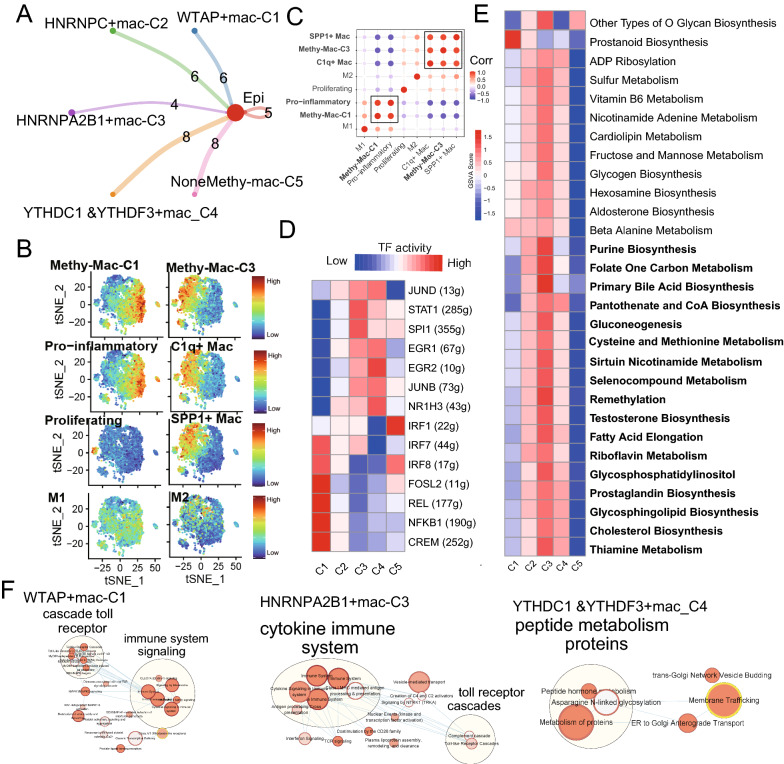
m^6^A regulators contributed to the production of tumor-associated macrophages5822 macrophages. **A** Cell–Cell communications between main m6A-related macrophage cells to epithelia cells by Cell chat analysis. **B** t-SNE plots of methy-mac-C1, methy-mac-C3, proinflammatory, C1q+, proliferating, SPP1 + mac, M1, and M2 macrophage signatures for 5582 macrophage cells. **C** Correlation plot for the above eight gene signatures in 5582 tumor macrophage cells. Methy-mac-C1 clusters are significantly related to proinflammatory macrophages, and methy-mac-C3 clusters are significantly related to SPP1 + and C1q + macrophage cells (p < 0.001, r > 0.5). **D** Heatmap showing significantly different TFs among m^6^A macrophage clusters by using pySCENIC in Python software to compare their average AUCs (Kruskal–Wallis test, p < 0.001). TF activity is scored using AUCell. **E** Heatmap showing significantly different activity of 41/113 metabolic signaling pathway scores by GSVA for 5582 cells among five methy-mac clusters (Kruskal–Wallis test, p < 0.001). **F** Enrichment cluster analysis for activated signaling ways and functions of m6A-related macrophage types in the Cytoscape by the REAC database

A total of 5822 macrophages were extracted from myeloid cells and divided into tumor-associated macrophages (5586 cells) and normal macrophages (236) (Additional file 2: Figure S4A). Then, we obtained five main m^6^A-mac clusters (Additional file 2: Figure S4B, C), including four clusters with the expression of m^6^A regulators (WTAP + mac-C1, n = 1432; HNRNPC + mac-C2, n = 1538; HNRNPA2B1 + mac-C3, n = 2169; YTHDC1 & YTHDF3 + mac-C4, n = 442) and one cluster without expression of m^6^A regulators (NoneMethy-mac-C5, n = 241) (Fig. 3A). The average number and cell proportion of m^6^A-mac NMF clusters showed a significant difference between normal and tumor (Chi-squared test p < 0.001, Additional file 2: Figure S4D). Similar to fibroblasts, we also observed different number of ligand-receptor links between these m^6^A related macrophage clusters to tumor epithelia cells.

Next, using the AddModulScore function of Seurat for these signatures (Fig. 3B, C, Additional file 2: Figure S4E and F) among TAMs, we found that WTAP + mac-C1 were significantly related to proinflammatory macrophages and HNRNPA2B1 + mac-C3 were significantly related to SPP1 + and C1q + macrophages [14]. Checkpoints expression also showed significantly different expression among five m^6^A-mac clusters (Additional file 2: Figure S4G). Furthermore, the SCENIC analysis of macrophages showed different activation of potential TFs among WTAP + mac-C1 and HNRNPA2B1 + mac-C3 clusters (Fig. 3D).

To assess the relationship between our m^6^A-mac clusters and special pathways, we used GSVA and detected that 41 out of 113 metabolic pathways were significantly different among the five m^6^A-mac clusters (Fig. 3E). Then, the 50 hallmark pathways showed broadly different activity among the five clusters (Additional file 2: Figure S4H). The enrichment map also revealed that the WTAP + mac-C1, HNRNPA2B1 + mac-C3 and TTHDC1 & YTHDF3 + mac-C4 had the different REACTOME pathway features (Fig. 3F).

### m^6^A-mediated T/B cell phenotypes underscored the antitumor immune response in CRC

**Fig. 4:**
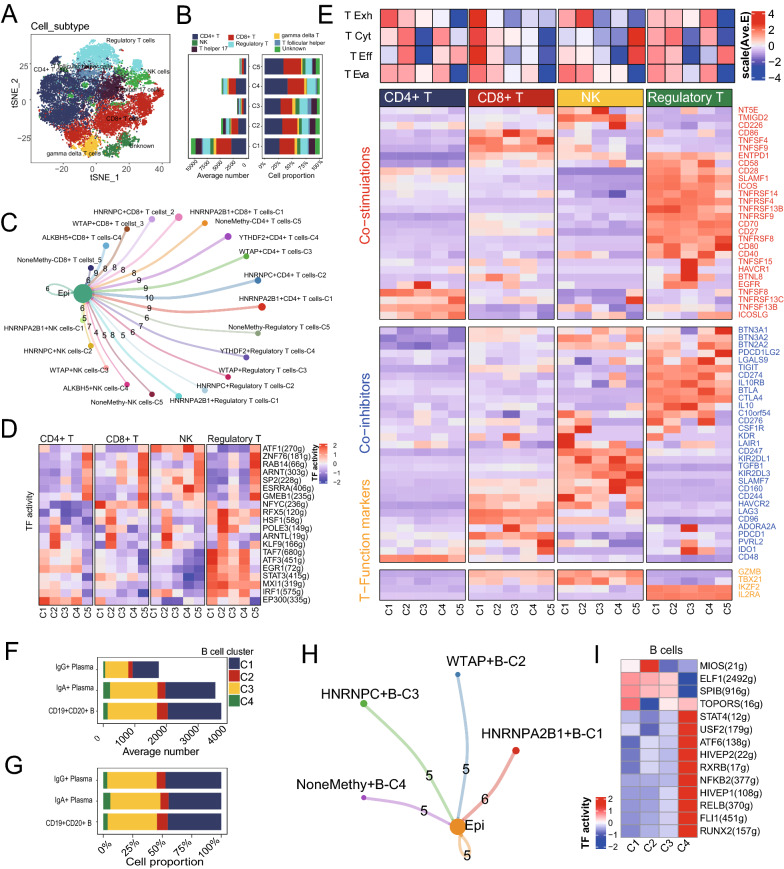
NMF clusters of m^6^A methylation regulators for T cells and B cells. **A** t-SNE plot for 23,115 T cells by 8 cell types in the SMC dataset, including CD + 4, CD + 8, Treg, NK, T helper 17, T follicular helper, gamma delta T, and Unknown. **B** Cell–Cell communications from main m6A-related T cells to epithelia cells by Cell chat analysis. (C1, HNRNPA2B1 dominant; C2, IGF2BP1 and HNRNPC dominant; C3, IFG2BP3, WTAP, and FTO dominant; C4, ALKBH5, YTHDF2, and YTHDC1 dominant). **C** Bar plot showing the number and percentage of methy-T cell clusters, including the cluster without m^6^A methylation regulator expression (n = 1265), among different cell types of T cells. **D** Heatmap showing significantly different TFs among m^6^A clusters in CD + 4, CD + 8, Treg, and NK cells by comparing their average AUC by using pySCENIC in Python (Kruskal–Wallis test, p < 0.001). TF activity is scored using AUCell. **E** Heatmap showing significantly different features among methy-T clusters in CD + 4, CD + 8, Treg and NK cells, including four T function signatures (T exhaustion score, T cytotoxic score, T effector score, and T evasion score), as well as some immune stimulators, inhibitors and T cell function marker genes (Kruskal–Wallis test, p < 0.001). **F** Cell–Cell communications between main m6A-related B cells types by Cell chat analysis. **G**, **H** Bar plot for the number and percentage of m^6^A clusters by using the NMF clustering algorithm for 9146 B cells as above. **J** Heatmap showing significantly different TFs among m^6^A clusters in total B cells

Among the detected 23,115 T cells, 8 main cell types, including CD4+, CD8+, Treg, NK, T helper 17, T follicular helper, etc., were identified to further analysis (Fig. 4A). A total of 5 m^6^A-related cell clusters were recognized by the NMF algorithm, and named as methy-T-C1 to methy-T-C5 (Fig. 4B), with the different numbers of ligand-receptor links between these m^6^A-related T cell clusters and tumor epithelia cells (Fig. 4C). Checkpoints expression analysis also showed significantly different expression among these m^6^A-related T cell clusters (Additional file 2: Figure S5A). Network regulatory analysis showed significant differential expression of TFs among these m^6^A clusters of T cells (Fig. 4D). In addition, to assess the overall effects of m^6^A-related T clusters on T cells, we found many differences in the average expression of immune genes of co-stimulation, co-inhibition, and some function-related markers. We also found many differences in the average expression of signatures among these m^6^A clusters of CD4 + T, CD8 + T, Treg, and NK T cells, including T exhaustion score, T cytotoxic score, T effector score, and T evasion score (Fig. 4E). Also, according to the DEGs, as listed in Additional file 1: Table S3, the enrichment map releveled that CD8 + T-C1, Treg-C1, CD8 + T-C4 and Treg-C4 had more immune function related terms (Additional file 2: Figure S5B). For 9146 B cells, NMF m^6^A clusters had similar ligand-receptor links to epithelial cells. No significant relationship was found between m^6^A-related B cell groups with IgG + plasma B cells, IgA + plasma B cells, and CD19 + CD20 + B cells, respectively (Fig. 4F–H). However, the heatmap still revealed the significantly different TFs among the m^6^A clusters (Fig. 4I).

### m^6^A-mediated TME patterns contributed the CRC prognosis and immunotherapy

**Fig. 5:**
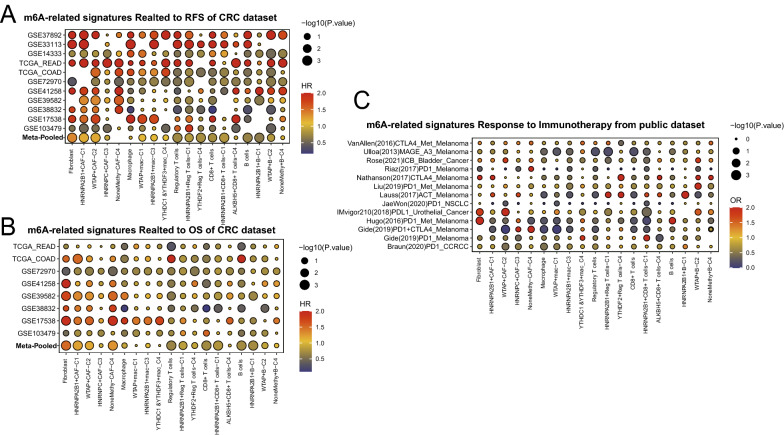
Overall of the prognosis and immunotherapy response of m6A-related cells types (GSVA score) in the bulk sequence from public cohorts. The cut-off were calculated by the survival R packages (**A**). RFS analysis (data from 11 CRC cohorts); **B** OS analysis (data from 8 CRC cohorts); **C** immunotherapy response analysis (data from 13 immunotherapy cohorts with response rate)

To obtain the signature of main CRC TME cell types, we re-calculated the DEGs of them in the CRC scRNA data and extracted the top 30 as the cell markers. Then, according to all DEGs of m^6^A-mediated TME cells (Additional file 1: Table S3), we used the GSVA to calculate the m^6^A sub-score, and to explore the prognosis of them in the CRC patients and pan-cancer by using the meta-analysis for OS and RFS in the 1892 and 2315 CRC patients from 8 and 11 CRC cohorts, as listed in the Fig. 5, respectively. All score were divided into two groups to conduct the cox regression analysis. Interestingly, along with the changing of the main dominated m^6^A genes in special m^6^A-mediated sub-cell types, we found that the recurrence-free survival (Fig. 5A, Additional file 2: Table S6) and overall survival (Fig. 5B, Additional file 2: Table S7) rates of them were significantly different among these sub-clusters, including CAF, macrophages, CD8 + T, Treg and B cells. In addition, we used the logistic regression method to observe the same meaningful phenomena of m6A sub TME cells for predicting the immune response for patients who underwent the immunotherapy in the 13 public cancer cohorts, including clear cell renal cell carcinoma (ccRCC), non-small cell lung cancer (NSCLC), met-melanoma, melanoma, urothelial cancer, and bladder cancer (Fig. 5C, and Additional file 1: Table S8). Finally, we inspected the prognosis of m6A sub clusters in the pan-cancer patients which were listed in Additional file 2: Figure S6A and B, and found that different cell sub clusters contributed to different cancers with the significant prognosis.

### m^6^A-mediated TME patterns enhanced the intercellular communication

**Fig. 6:**
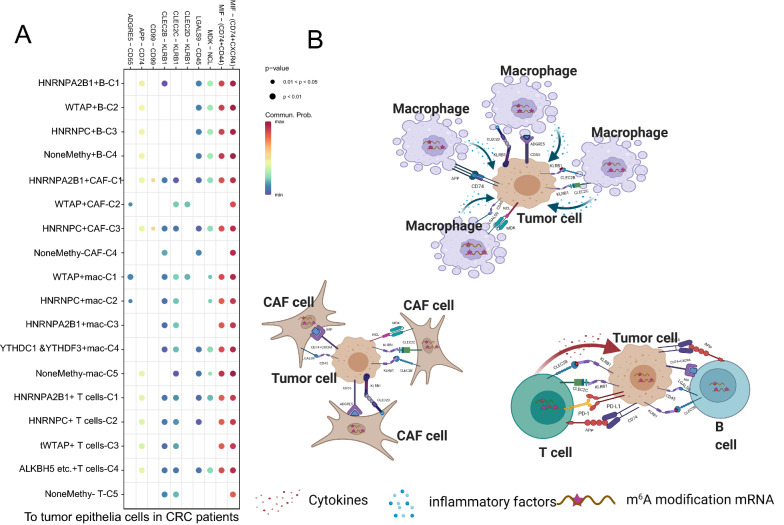
Cell–Cell communications from main m6A-related TME cells to epithelial cells. **A** The significantly related ligand–receptor interactions from main m6A-related clusters to epithelial cells. **B** Hypothesis of the mechanism of m^6^A clustering TME cells affecting cell communication

By the cell-chat analysis, we listed all ligand-receptor pairs of intercellular communication, including MIF −  (CD74 + CXCR4), MIF −  (CD74 + CD44), MDK–NCL, LGALS9 − CD45, CLEC2D − KLRB1, CLEC2C − KLRB1, CLEC2B − KLRB1, APP − CD74, CD99 − CD99, and ADGRE5 − CD55, were existed from m^6^A sub clusters to the tumor epithelial cells (Fig. 6A and Additional file 1: Table S4). Herein, the hypothesis of potential mechanism between them was demonstrated in Fig. 6B. Each m^6^A subtypes might have different strengths and ligand-receptor pairs with tumor epithelial clusters, which suggests that m^6^A-mediated TME cells might have more interactions with tumor cells and thus contribute to the progress of CRC.

## Discussion

To date, several studies have revealed the correlation between RNA m^6^A modification and the pathogenesis of colorectal cancer [36–40]. However, only a few have investigated the potential tumorigenic role of m^6^A-modified single cells. In the present study, we, for the first time, have comprehensively explored m^6^A modification regulators of main cell types in the TME of colorectal cancer and further identified the diversity cell–cell interaction between m^6^A associated TME cell subtypes and tumor cells at the 10X Genomic single-cell sequence level. This unique and new perspective allowed us to understand how RNA m^6^A modification of these diverse cellular components of TME affects the fate of individual CRC patients.

Cancer epithelial cells constitute the majority of tumor tissue and drive tumor development. Meanwhile, the heterogeneity of cancer epithelial cells indicates the responsiveness of patients to treatment and determines prognosis. Besides cancer epithelial cells, TME cells, such as multiple types of stromal cells, vascular endothelial cells and infiltrating immune cells, all support growth and promote immune evasion of solid tumors. In the study, we found that the TME cells, including stromal cells, macrophages, T cells and B cells all manifested the diverse m^6^A regulatory patterns and the extensive communication with tumor epithelial cells based on the single-cell analysis. Furthermore, Cellphone analysis showed that ligand-receptor pairs, including MIF −  (CD74 + CXCR4), MIF −  (CD74 + CD44), MDK–NCL, LGALS9 − CD45, CLEC2D − KLRB1, CLEC2C − KLRB1, CLEC2B − KLRB1, APP − CD74, CD99 − CD99, and ADGRE5 − CD55 mediated the communication between m^6^A associated subtypes of TME cells and tumor epithelial cells.

Cancer-associated fibroblasts (CAFs), as one of the critical components of stromal cells, were classified as pan-myCAFs, pan-dCAFs, pan-iCAFs, pan-nCAFs, and pan-pCAFs, according to specific molecular characteristics [35]. To date, few studies have revealed the potential role of RNA m^6^A modification in CAFs. In our study, we found that m^6^A-mediated fibroblasts manifested more extensive communications with tumor epithelial cells compared with non-m^6^A-mediated fibroblasts. Furthermore, WTAP-fibroblasts had a strong relationship with inflammatory-CAFs and the elevated expression of proinflammatory factors, such as CXCL1, CXCL2, CXCL3, CCL2, IL-6 and IL-7. Pathway analysis also revealed the participation of CAFs in IL-17 signaling pathway, TNF signaling pathway and the innate immune associated pathways. CAFs may shape an immunosuppressive microenvironment through the secretion of CXCL1, IL6 and CCL2 [41–44]. Therefore, we speculated that RNA m^6^A modification CAFs may form the immunosuppressive interaction with tumor cells to promote the progress and metastasis of tumor.

Nowadays, increasing research has revealed the significant role of RNA m^6^A methylation in the regulation and reprogramming of immune cells [12, 13, 45–47]. In the study of Lihui Dong and colleagues [45], C1q + TAMs, a subtype of TAMs, were found to be regulated by an RNA m^6^A program and promote CD8 + T cell dysfunction by expressing Ebi3 transcript with decreased m^6^A level. In addition, Lei Zhang et al. [48] reported that SPP1 + TAMs had a pro-angiogenic signature and weakened tumor immunity. By NMF clusters, we found that m^6^A-mediated subtypes of macrophages all manifested extensive communications with tumor cells. Correlation analysis showed that HNRNPA2B1 + mac-C3 was significantly related to SPP1 + and C1q + macrophages, and further prognosis analysis revealed the inverse correlation between the expression with survival probability. The metabolic process had a profound influence on TAMs and thus modulated cancer progression and immune responses, including glucose, glutamine and fatty acid metabolism [49]. Our research found that m^6^A-mediated macrophage, especially for HNRNPA2B1 + mac-C3 subtypes, manifested obvious activation of metabolism-related pathways, such as purine biosynthesis, gluconeogenesis, and cysteine and methionine metabolism et al. Moreover, except for macrophages, we found that m^6^A-mediated T cells (CD8+, CD4+ and regulatory T cells), NK cells and B cells also showed extensive interaction with tumor cells. Furthermore, m^6^A-mediated subtypes of four main T cells exhibited variable T cell active and inactive characteristics. These findings all indicate the significant role of RNA m^6^A methylation in immune escape and the tumor-promoting effect of macrophages and T cells.

To identify cell-specific gene regulatory networks, we performed an analysis of TFs at the single-cell level. In general, each subtype of CAFs, macrophages, B cells and various types of T cells all manifested distinct TFs characteristics. For CAFs, WTAP-fibroblasts exhibited a unique TF gene signature, such as ETS1, CEBPB, IRF1, REL and NFKB1. Previous studies have revealed the relationship between m^6^A modification and the expression of ETS1, CEBPB, IRF1 and REL, which suggests the role of m^6^A in the regulation of CAFs [50–53]. In addition, for macrophages, we observed higher activity of SPI1 and STA1 on HNRNPA2B1-mac. Similarly, the correlation between m^6^A modification and both SPI1 and STA1 was reported in previous research [52, 54–56]. Moreover, for B and T cells, we also found distinct TF characteristics of m^6^A-mediated cell subtypes. To sum up, m^6^A-mediated cell subtypes may modulate distinct TF regulatory networks to reshape and reprogram the TME. Finally, cell network analysis revealed that these m^6^A-mediated TME cells were closely connected and communicated with tumor cells. Notably, either m^6^A-mediated CAFs or immune cell subtypes had more communication with cancer epithelial cells, indicating that formation of an immunosuppressive tumor microenvironment might partially be determined by RNA m^6^A methylation.

Considering the complex intrinsic patterns of RNA m^6^A methylation in TME cells, we comprehensive summarized the relationships of these sub clusters' scores with prognosis and immune response from the public bulk RNA-seq cohorts. Clearly, the patients with different domination of m6A regulators of the TME cells had huge prognosis differences of CRC and exceedingly distinguished the immune response for patients who underwent ICB therapy, especially for the CAFs and macrophages, which revealed that the critical role of TME m^6^A for CRC patients in further research.

As a preliminary study, the major limitations of our analysis were that the low depth of scRNA-seq and the inadequate samples, and our conclusion need to acquire verification in more patients. Compared with bulk RNA-seq, the scRNA-seq of some m^6^A regulators in CRC would typically be minor and had more zero observation, which might contribute to the bias of the clustering method in our study. Nonetheless, the scRNA-seq analysis still provides us a novel view to reveal the characteristics of m^6^A methylation regulators in various TME single cells to reduce the tumor heterogeneity in CRC, which is a key forward step for clinical practice.

## Conclusions

We, for the first time, identified specific RNA m^6^A-modificated cell subtypes of TME cells by using the single-cell sequencing analysis method and revealed the m^6^A methylation mediated intercellular communication of tumor microenvironment in the regulation of tumor growth and antitumor immunomodulatory processes.

## Supplementary Information

**Additional file 1: Table S1.** The information of including cohorts in the study. **Table S2.** The geneset lists using in the study. **Table S3.** the list of methylation-related genes in each NMF sub cluster of CRC. **Table S4.** Complex intercellular communication networks between the m6A-mediated immune cells clusters and epithelia cells. **Table S5.** REAC analysis for Each m6A-mediated subClusters. **Table S6.** OS of m6A-related Cell types In the Bulk sequences. **Table S7.** RFS of m6A-related Cell types In the Bulk sequences. **Table S8.** m6A-related Cell type signatures to response of ICB immunotherapy in the Bulk sequences.

**Additional file 2: Figure S1.** The expression of m^6^A regulators and the association of them with ImmuneScore in CRC (Related to Fig. 1). **A** The expression of m^6^A regulators in CRC samples with main TME cell types. **B** The association of m^6^A expression with ImmuneScore in different TME cell types in scRNA-seq data. **Figure S2.** Exploration of cancer-association fibroblast cells in CRC. Related to Fig. 2. **A** t-SNE plot for stromal cells reveals different distribution of fibroblasts and non-fibroblasts between normal mucosa (2197 cells) and CRC tissue (2736 cells). ** B** Heatmap showing the top four genes in normal mucosa and tumor fibroblasts. ** C** The prognosis of fibroblast cells by xCell in TCGA-COAD and READ. ** D** Trajectory analysis for cancer-association fibroblast cells. **E** Top genes in four NMF clusters for 3462 fibroblast cells, including m^6^A-fib-C1 (1939 cells, HNRNPA2B1 dominant), m^6^A-fib-C2 (245 cells, WTAP dominant), m^6^A-fib-C3 (1194 cells, HNRNPC dominant), and m^6^A-fib-C4 (84 cells, no m^6^A methylation). ** F** Heatmap showing the average expression of cell surface protein genes in four m^6^A fibroblast cell clusters (Kruskal–Wallis test, p < 0.001). **Figure S3.** Heatmap of pseudotime Trajectory analysis for TME cell subtypes in TME main cell types of CRC, including macrophage, and B cells, as well as four types of T cells (CD8 + T, CD4 + T, Treg, and NK T cells). **Figure S4.** Features of m^6^A-mac clusters in CRC. Related to Fig. 3.** A** t-SNE plot of 5822 macrophages by their source class in SMC dataset. **B** Heatmap showing the correlation of four m^6^A methylation regulator clusters by NMF, named as methy-mac-C1 (n = 1432), methy-mac-C2 (n = 1538), methy-mac-C3 (n = 2169), and methy-mac-C4 (n = 442) for 5822 macrophage cells. ** C** Heatmap showing the different expressions for m^6^A methylation regulators in macrophages cells. ** D** Bar plot showing the number and percentage of methy-mac clusters, including the cluster without m^6^A methylation regulator expression between tumor and normal samples. ** E **and** F** The m^6^A-mac clusters were related to proinflammatory, proliferating and SPP1 + macrophage cells. ** G** The distribution of checkpoints gene expression among five m^6^A-mac clusters. ** H** The activity of hallmark pathways among five m^6^A-mac clusters. **Figure S5.** The immune features and prognosis of m^6^A-T cell clusters in CRC. Related to Fig. 4.** A** The distribution of checkpoints gene expression among m^6^A-related T sub cluster cells, including CD4+, Cd8 + NK, and Regulatory T cells. ** B** Enrichment cluster analysis for activated signaling ways and functions of m6A-related macrophage types in the Cytoscape by the REAC database. **Figure S6.** The dynamic effects to the prognosis of m6A-related subtype TME cells in the Pan Cancer patients. Related to Fig. 5. ** A** The significant prognosis of the m6A-related cells, including CAF subtype cells (HNRNPA2B1 + CAF-C1; WTAP + CAF-C2; HNRNPC + CAF-C3; NoneMethy-CAF-C4), macrophage subtype (WTAP + mac-C1;HNRNPA2B1 + mac-C3;YTHDC1 &YTHDF3 + mac_C4), T subtype cells (HNRNPA2B1 + CD8 + T cells-C1;ALKBH5 + CD8 + T cells-C4; HNRNPA2B1 + Reg T cells-C1; YTHDF2 + Reg T cells-C4), and B subtype cells (HNRNPA2B1 + B-C1; WTAP + B-C2; NoneMethy + B-C4). ** B** Tables showed dynamic changes of the number of the prognosis among different subtypes in Pan-Cancer patients.

## Data Availability

All data are available in a public, open access repository. The datasets used and/or analyzed during the current study are available in the Gene Expression Omnibus (GEO, https://www.ncbi.nlm.nih.gov/geo/) and The Cancer Genome Atlas (TCGA) network (https://cancergenome.nih.gov/). R and other custom scripts for analyzing data are available upon reasonable request.
