# Supplementary material for: Single-cell N6-methyladenosine regulator patterns guide intercellular communication of tumor microenvironment that contribute to colorectal cancer progression and immunotherapy
Source: J Transl Med. 2022 May 4;20:197. doi: 10.1186/s12967-022-03395-7 (PMC9066909; doi:10.1186/s12967-022-03395-7)
Supplement: Supplementary file 1 — Additional file 1: Table S1. The information of including cohorts in the study. Table S2. The geneset lists using in the study. Table S3. the list of methylation-related genes in each NMF sub cluster of CRC. Table S4. Complex intercellular communication networks between the m6A-mediated immune cells clusters and epithelia cells. Table S5. REAC analysis for Each m6A-mediated subClusters. Table S6. OS of m6A-related Cell types In the Bulk sequences. Table S7. RFS of m6A-related Cell types In the Bulk sequences. Table S8. m6A-related Cell type signatures to response of ICB immunotherapy in the Bulk sequences. [file 12967_2022_3395_MOESM1_ESM.pdf]

Description
TableS1 The information of including cohorts in the study.
TableS2 The geneset lists using in the study
TableS3: the list of methylation-related genes in each NMF sub cluster of CRC
TableS4: Complex intercellular communication networks between the m6A-mediated immune cells clusters and epithelia cells
TableS5: REAC analysis for Each m6A-mediated subClusters
TableS6:OS of m6A-related Cell types In the Bulk sequences
TableS7:RFS of m6A-related Cell types In the Bulk sequences
TableS8:m6A-related Cell type signatures to response of ICB immunotherapy in the Bulk sequences

TableS1 The information of including cohorts in the study.				
No.	Cohort Names	Samples/cells	Prognostic data type	Source Website
1	GSE132465	65362	none	https://www.ncbi.nlm.nih.gov/geo/
2	GSE17538	238	RFS,OS	https://www.ncbi.nlm.nih.gov/geo/
3	GSE33113	96	OS	https://www.ncbi.nlm.nih.gov/geo/
4	GSE37892	130	RFS	https://www.ncbi.nlm.nih.gov/geo/
5	GSE38832	122	RFS,OS	https://www.ncbi.nlm.nih.gov/geo/
6	GSE39582	585	RFS,OS	https://www.ncbi.nlm.nih.gov/geo/
7	GSE41258	182	OS	https://www.ncbi.nlm.nih.gov/geo/
8	GSE39084	71	RFS,OS	https://www.ncbi.nlm.nih.gov/geo/
9	GSE14333	251	RFS,OS	https://www.ncbi.nlm.nih.gov/geo/
10	GSE72970	124	RFS,OS	https://www.ncbi.nlm.nih.gov/geo/
11	GSE103479	156	RFS,OS	https://www.ncbi.nlm.nih.gov/geo/
12	TCGA-COAD	539	RFS,OS	https://xenabrowser.net/datapages/
13	TCGA-READ	159	RFS,OS	https://xenabrowser.net/datapages/

TableS2 The Geneset lists using in the study

Gene Set names	Types	Genes						
m6Agenes	Erasers	CBLL1	KIAA1429	METTL14	METTL3	RBM15	RBM15B	WTAP
	Readers	ZC3H13	ELAVL1	FMR1	HNRNPA2B1	HNRNPC	IGF2BP1	IGF2BP2
		IGF2BP3	LRPPRC	YTHDC1	YTHDC2	YTHDF1	YTHDF2	YTHDF3
	Writers	ALKBH5	FTO					
CCR_CAF_genes	pan-myCAF	ADIRF	ACTA2	MYH11	TAGLN	SPARCL1	MCAM	A2M
		PPP1R14A	CRIP2	ADAMTS1	CSRP2	NDUFA4L2	TPM1	MAP1B
		RGS5	MEF2C	CALM2	APOLD1	OAZ2	MGST3	ISYNA1
		NDRG2	ID3	RGS16	CYB5R3	CRYAB	OLFML2A	TIMP3
		MYLK	IGFBP7	CRIP1	TINAGL1	TPM2	PTP4A3	CPE
		FRZB	PRKCDBP	CSRP1	CAV1	ADAMTS4	GJA4	
		CPM	PGF	GUCY1B3	UBA2	YIF1A	PHLDA1	
		GUCY1A3	FILIP1	FAM13C	NDUFS4	ITGB1	KCNE4	
	pan-dCAF	COL1A1	THBS2	CTHRC1	COL3A1	LUM	COL1A2	LGALS1
		COL12A1	MMP2	INHBA	TNFAIP6	VCAN	ADAM12	THY1
		EMP1	ANGPTL2	RARRES2	SULF1	IGFBP3	COL8A1	GREM1
		P4HA3	CRABP2	TPM4	LOXL1	CPXM1	COL6A1	ASPN
		YIF1A	SNAI2	C1S	TMEM176B	CTGF	MORF4L2	UAP1
		LMNA	CYP1B1	MGP	ANGPTL4	TIMP1	SERPINF1	ITGB1
		COL5A1	POSTN	SERPINE1	LOXL2	COL11A1	CTSK	TGFBI
		FN1	STEAP1	SPON2	PLAUR	SPHK1	LOX	HTRA3
		DCN	ITGA5	ANTXR1	RIN2	TMEM119	TNFRSF12A	C1R
		PDLIM4	ITGA11	PRSS23	COL6A2	SFRP2	PLAU	
	pan-iCAF	CFD	GPC3	C3	ADH1B	IGF1	EFEMP1	PODN
		PLA2G2A	DPT	WISP2	CCDC80	SFRP2	PTGDS	DCN
		FHL2	ELN	KLF4	TMEM176B	SERPINF1	FHL1	GPX3
		RARRES1	CYR61	IGFBP5	FBLN1	MGST1	MFAP4	CTGF
		SEPP1	CXCL12	ABI3BP	GSN	TMEM176A	FIBIN	C1R
		MGP	C1S	IGFBP6	CYP1B1	CST3	SLC40A1	SFRP4
	pan-pCAF	NUSAP1	DIAPH3	LOXL2	ADAM12	LOX	POSTN	THY1
		COL1A1	COL8A1	COL6A1	COL6A2	CD248	FN1	COL12A1
		CTHRC1	COL5A1	LOXL1				
	pan-iCAF-2	IER3	CXCL2	ICAM1	TNFAIP2	NFKBIA	NR4A1	CCL2
		GEM	NR4A3	APOD	SAT1	UAP1	OGN	GFPT2
		ABI3BP	GADD45B	DUSP1	RARRES1	CST3	ABL2	EGR1
		SOD2	FOSB	PIM1	ZFP36	CLU	C3	CYP1B1
		JUNB	THBS1	CDKN1A				
Fibroblasts_cell_marker_genes	Surface_markers	PARM1	APOC3	CSPG4	CD36	SUSD2	SDC1	TREM1
		LRRN3	THY1	BAMBI	P2RY6	CD34	PI16	EBP
		GPC3	RAMP2	CLDN1	ICAM1	MUSK	CXCR4	NCAM1
		EFNB1						
T_cell marker_genes	Co-inhibitors	ADORA2A	BTLA	BTN2A2	BTN3A1	BTN3A2	BTNL2	C10orf54
		CSF1R	HAVCR2	IDO1	IL10	IL10RB	KDR	KIR2DL1
		SLAMF7	TGFB1	TIGIT	VRCN1	VTCN1	CD247	CTLA4
		CD160	CD244	CD274	CD276	CD48	CD96	
		KIR2DL2	KIR2DL3	LAG3	LAIR1	LGALS9	PVRL2	
		PDCD1	PDCD1LG2					
	Co-stimuiations	BTNL8	CD226	CD27	CD28	CD40	CD58	CD70
		SLAMF1	TMIGD2	TNFRSF13B	TNFRSF13C	TNFRSF14	TNFRSF4	TNFRSF8
		TNFSF8	TNFSF9	ENTPD1	NT5E	ICOS	TNFSF4	TNFSF15
		CD80	CD86	EGFR	HAVCR1	TNFSF18	ICOSLG	TNFSF13B
		TNFRSF9	TNFSF13					
	T-function Genes	CD3E	CD4	CD8B	FOXP3	GZMB	PRF1	TBX21
		IL2RA	IKZF2					
	Immune_Checkpoint	CD274	CTLA4	LAG3	TIM3	TNFRSF9	TIGIT	CD226
		CD7	GZMB	PRF1	TNFRSF18	TNFRSF4	HAVCR2	NLG1

	**s**	CD4	CD8A	CD8B	FOXP3	IL2	CXCL8	PDCD1
		IFNG						
T_Function _score_gene list	**Teffectscore**	CD8A	CXCL10	CXCL9	GZMA	GZMB	IFNG	PRF1
		TBX21						
	**Cytotoxicscore**	CST7	GZMA	GZMB	IFNG	NKG7	PRF1	
	**Exhaustionscore**	CTLA4	HAVCR2	LAG3	PDCD1	TIGIT		
	**Tevasionscore**	B2M	IFNGR1	JAK2	SOCS1	TAP1	TAP2	TAPBP
		AGO2	AHSA1	ANAPC15	ARF6	ATG14	ATG7	ATG9A
		PDCD6IP	PDSS2	PI4KB	PIGK	PIGT	PKN2	PPP1CA
		PDIA3	PIGS	PIGU	PPP2R2A	PSMB8	RB1CC1	RBM15
		IFNAR1	IFNAR2	IKBKB	INO80	IPPK	IRF9	JAGN1
		GPI1	IFNGR2	IKBKG	IRF1	JAK1	OTUD5	PTPN2
		BRPF1	CAD	CALR	CEP55	CHMP5	COX6C	CREBBP
		RCE1	RGP1	RIC1	S100PBP	SARNP	SETD1A	SETDB1
		TMEM127	TNFRSF1B	UBE2G2	UBE2N	VDAC2	VPS29	VPS35
		MED16	MED23	MED24	MGAT1	MTA2	N6AMT1	NCBP1
		ATG12	ATG3	ATG5	BECN1	CHIC2	DNTTIP1	EMC8
		DPH5	EIF3H	EMC2	EMC3	EMC4	EMC6	F8A
		TBK1	TFRC	TGFBR2	TMEM208	TRADD	TRPM7	UBR5
		MAP3K7	MEN1	MOGS	NDUFA13	NXT1	OTULIN	H2-K1
		VPS16	WDR7	WDR83	WWP2	YAP1	ZC3H3	ACTB
		ADAR	ATG10	ATG101	CFLAR	FITM2	GPAA1	PCED1B
		ATP13A1	ATXN7L3	BC003331	BCL2L1	BOLA3	BRAT1	IST1
		PPP1R8	PPP2R3C	PRKCSH	PSMB9	PSMG1	PTAR1	GLS
		RIC8	RNF31	SCAF4	SMG7	TAB1	TAB2	VPS13A
		JMJD6	KAT6A	KLF16	KMT2A	LIPT2	MAPK1	HSPA13
		RBCK1	STAT1	STAT2	TNFAIP3	TNFRSF1A	TRAF2	ACAD9
		CUL3	DCP1A	DET1	DICER1	DNAJC13	DOT1L	PCBP2
		SLC25A32	SPCS1	SRRT	SRSF7	STOML2	SUSD6	HDAC1
		VPS4B	WIPI2	ZCCHC14	FAM58B	FAS	FNTB	GALE
		NDUFAF6	NPLOC4	NUP188	UFC1	UFL1	USP7	UXS1
		ERAP1	ERP44	FADD	HCFC2	HDGFRP2	HEXIM1	HIRA

e list of methylation-related genes in each NMF sub clust

Methylation-celltype	Methylation_related gene	Methylation-celltype	Methylation_related gene	Methylation-celltype	Methylation_related gene
Fibroblast_C1	HNRNPA2B1	macrophage_C1	WTAP	CD8+ T cells_C1	HNRNPA2B1
Fibroblast_C1	TUBA1B	macrophage_C1	SOD2	CD8+ T cells_C1	ACTB
Fibroblast_C1	TAGLN	macrophage_C1	FTH1	CD8+ T cells_C1	STMN1
Fibroblast_C1	STMN1	macrophage_C1	BTG1	CD8+ T cells_C1	HMGB2
Fibroblast_C1	HSP90AA1	macrophage_C1	BCL2A1	CD8+ T cells_C1	CTSD
Fibroblast_C1	HSPA1A	macrophage_C1	CD44	CD8+ T cells_C1	TPI1
Fibroblast_C1	ACTN4	macrophage_C1	EREG	CD8+ T cells_C1	TUBB
Fibroblast_C1	HSPA1B	macrophage_C1	C15orf48	CD8+ T cells_C1	RANBP1
Fibroblast_C1	CYR61	macrophage_C1	IL1B	CD8+ T cells_C1	PSME2
Fibroblast_C1	CD74	macrophage_C1	NINJ1	CD8+ T cells_C1	CORO1A
Fibroblast_C1	ACTA2	macrophage_C1	IL1RN	CD8+ T cells_C1	ANXA6
Fibroblast_C1	POSTN	macrophage_C1	G0S2	CD8+ T cells_C1	HMGN2
Fibroblast_C2	WTAP	macrophage_C1	PNRC1	CD8+ T cells_C1	HSP90AA1
Fibroblast_C2	SOD2	macrophage_C1	AQP9	CD8+ T cells_C1	PTTG1
Fibroblast_C2	C11orf96	macrophage_C1	NAMPT	CD8+ T cells_C1	DUT
Fibroblast_C2	FTH1	macrophage_C1	SERPINB9	CD8+ T cells_C1	ENO1
Fibroblast_C2	TNFAIP3	macrophage_C1	SRGN	CD8+ T cells_C1	PCNA
Fibroblast_C2	CXCL2	macrophage_C1	EIF1	CD8+ T cells_C1	PGAM1
Fibroblast_C2	B4GALT1	macrophage_C1	ATP2B1	CD8+ T cells_C1	PKM
Fibroblast_C2	NFATC2	macrophage_C1	NFKB1	CD8+ T cells_C1	HLA-DRB1
Fibroblast_C2	SAT1	macrophage_C1	TIMP1	CD8+ T cells_C1	HNRNPA3
Fibroblast_C2	SERPINE1	macrophage_C1	TNIP3	CD8+ T cells_C1	PSMB9
Fibroblast_C2	PIM3	macrophage_C1	CCL3	CD8+ T cells_C1	CXCL13
Fibroblast_C2	LIF	macrophage_C1	PLAUR	CD8+ T cells_C1	XRCC5
Fibroblast_C2	HNRNPH1	macrophage_C1	CCL20	CD8+ T cells_C1	CD53
Fibroblast_C2	NAMPT	macrophage_C1	SAT1	CD8+ T cells_C1	PDIA6
Fibroblast_C2	TNFAIP2	macrophage_C1	CXCL2	CD8+ T cells_C1	ACTG1
Fibroblast_C2	CXCL8	macrophage_C1	PTGS2	CD8+ T cells_C1	RPA3
Fibroblast_C2	KDM6B	macrophage_C1	TNFAIP6	CD8+ T cells_C1	ECH1
Fibroblast_C2	HMGA1	macrophage_C1	EHD1	CD8+ T cells_C1	HMGB1
Fibroblast_C2	FTL	macrophage_C1	MIR3945HG	CD8+ T cells_C1	RBPJ
Fibroblast_C2	IER3	macrophage_C1	NFKBIA	CD8+ T cells_C1	COX8A
Fibroblast_C2	IL6	macrophage_C1	IL1A	CD8+ T cells_C1	IDH2
Fibroblast_C2	CDC42SE1	macrophage_C1	KYNU	CD8+ T cells_C1	POLR2G
Fibroblast_C2	CXCL3	macrophage_C1	TNIP1	CD8+ T cells_C1	PTBP1
Fibroblast_C2	PTPRS	macrophage_C1	CCL3L3	CD8+ T cells_C1	PSMA4
Fibroblast_C2	IL1R1	macrophage_C1	CFLAR	CD8+ T cells_C2	HNRNPC
Fibroblast_C2	NFKBIA	macrophage_C1	LINC00936	CD8+ T cells_C3	WTAP
Fibroblast_C2	CHI3L1	macrophage_C1	CCL4	CD8+ T cells_C4	ALKBH5
Fibroblast_C2	GPRC5A	macrophage_C1	THBS1	CD8+ T cells_C4	YTHDF2
Fibroblast_C2	NEAT1	macrophage_C1	CCRL2	CD8+ T cells_C4	YTHDC1
Fibroblast_C2	ADAM17	macrophage_C1	TNFAIP8	CD8+ T cells_C4	ZFP36
Fibroblast_C2	PNRC1	macrophage_C1	CD300E	CD8+ T cells_C4	ZC3H13
Fibroblast_C2	BTG1	macrophage_C1	THAP2	CD4+ T cells_C1	HNRNPA2B1
Fibroblast_C2	MT2A	macrophage_C1	ATP13A3	CD4+ T cells_C2	HNRNPC
Fibroblast_C2	ICAM1	macrophage_C1	S100A12	CD4+ T cells_C3	WTAP
Fibroblast_C2	TSC22D1	macrophage_C1	SAMSN1	CD4+ T cells_C4	YTHDF2
Fibroblast_C2	RPL3	macrophage_C1	SLC25A37	CD4+ T cells_C4	ALKBH5
Fibroblast_C2	RPL7	macrophage_C1	CTNNB1	CD4+ T cells_C4	YTHDC1
Fibroblast_C2	RNF19B	macrophage_C1	CXCL3	CD4+ T cells_C4	ZC3H13
Fibroblast_C2	CDKN1A	macrophage_C1	ANXA5	Regulatory T cell	HNRNPA2B1
Fibroblast_C2	NFKBIZ	macrophage_C1	GK	Regulatory T cell	CD53
Fibroblast_C2	MCL1	macrophage_C1	NLRP3	Regulatory T cell	HSP90AA1

Fibroblast_C2	PTGDS	macrophage_C1	ACSL1	Regulatory T cell	HSP90AB1
Fibroblast_C2	EEF2	macrophage_C1	B4GALT1	Regulatory T cell	HNRNPC
Fibroblast_C2	CD44	macrophage_C1	DENND5A	Regulatory T cell	WTAP
Fibroblast_C2	ERRFI1	macrophage_C1	VCAN	Regulatory T cell	YTHDF2
Fibroblast_C2	SLC39A14	macrophage_C1	BASP1	Regulatory T cell	YTHDC1
Fibroblast_C2	NFKB1	macrophage_C1	CREM	Regulatory T cell	ALKBH5
Fibroblast_C2	ABL2	macrophage_C1	INHBA	Regulatory T cell	ZC3H13
Fibroblast_C2	TFPI2	macrophage_C1	DRAM1	Regulatory T cell	ELAVL1
Fibroblast_C2	GEM	macrophage_C1	CD55	Regulatory T cell	MALAT1
Fibroblast_C2	KLF6	macrophage_C1	PHLDA1	NK cells_C1	HNRNPA2B1
Fibroblast_C2	PTGS2	macrophage_C1	TNFRSF1B	NK cells_C2	HNRNPC
Fibroblast_C2	LHFPL2	macrophage_C1	VIM	NK cells_C3	WTAP
Fibroblast_C2	CCDC71L	macrophage_C1	MAP3K8	NK cells_C4	ALKBH5
Fibroblast_C2	SET	macrophage_C1	FCN1	NK cells_C4	YTHDF1
Fibroblast_C2	RYBP	macrophage_C1	TRAF1		
Fibroblast_C2	ATP13A3	macrophage_C1	EIF1B		
Fibroblast_C2	GFPT2	macrophage_C1	OLR1		
Fibroblast_C2	CTNNB1	macrophage_C1	SLC7A5	**Methylation-celltype**	**Methylation_related gene**
Fibroblast_C2	PLK3	macrophage_C1	GCH1	B cells_C1	HNRNPA2B1
Fibroblast_C2	BMP2	macrophage_C1	DUSP4	B cells_C1	TUBA1B
Fibroblast_C2	ZC3H12A	macrophage_C1	CD48	B cells_C2	WTAP
Fibroblast_C2	EMP1	macrophage_C1	FNDC3B	B cells_C2	TPT1
Fibroblast_C2	CEBPB	macrophage_C1	SERPINA1	B cells_C2	PFDN5
Fibroblast_C2	NACA	macrophage_C1	IRAK2	B cells_C2	YPEL5
Fibroblast_C2	RIPK2	macrophage_C1	PIM3	B cells_C2	H3F3B
Fibroblast_C2	PLAUR	macrophage_C1	SLC2A3	B cells_C2	EIF1
Fibroblast_C2	ZFP36L1	macrophage_C1	MARCKS	B cells_C3	HNRNPC
Fibroblast_C2	SDC4	macrophage_C1	LINC00152	B cells_C4	IER2
Fibroblast_C2	C1orf56	macrophage_C1	REL	B cells_C4	C12orf57
Fibroblast_C2	REL	macrophage_C1	CCL4L2	B cells_C4	GADD45B
Fibroblast_C2	PIM1	macrophage_C1	RNF144B	B cells_C4	MT-ND2
Fibroblast_C2	ABCA1	macrophage_C1	ISG20	B cells_C4	CITED2
Fibroblast_C2	EIF3L	macrophage_C1	PPP1CB	B cells_C4	MT-CO2
Fibroblast_C2	IGF1	macrophage_C1	ARFGAP3	B cells_C4	LINC00152
Fibroblast_C2	EPAS1	macrophage_C1	BIRC3	B cells_C4	PRDM1
Fibroblast_C2	NINJ1	macrophage_C1	METTL9	B cells_C4	MT-CO1
Fibroblast_C2	LMNA	macrophage_C1	PDE4DIP	B cells_C4	AKAP9
Fibroblast_C2	TRIB1	macrophage_C1	STX11	B cells_C4	ACADVL
Fibroblast_C2	PTPN1	macrophage_C1	SERPINB1	B cells_C4	MT-ND4
Fibroblast_C2	LRRFIP1	macrophage_C1	ELL2	B cells_C4	N4BP2L2
Fibroblast_C2	G0S2	macrophage_C1	DUSP6	B cells_C4	FOSB
Fibroblast_C2	NR4A3	macrophage_C1	MCEMP1	B cells_C4	MT-CYB
Fibroblast_C2	NPC2	macrophage_C1	ARL8B	B cells_C4	FOS
Fibroblast_C2	S100P	macrophage_C1	BACH1	B cells_C4	JUN
Fibroblast_C2	TPT1	macrophage_C1	IL6	B cells_C4	MT-CO3
Fibroblast_C2	ARID5B	macrophage_C1	PMAIP1	B cells_C4	PPP1R10
Fibroblast_C2	TOP1	macrophage_C1	SMIM3		
Fibroblast_C2	SLC16A1	macrophage_C1	GRAMD1A		
Fibroblast_C2	SDCBP	macrophage_C1	KMO		
Fibroblast_C2	HIF1A	macrophage_C1	TWISTNB		
Fibroblast_C2	MARCKSL1	macrophage_C1	NUMB		
Fibroblast_C2	ZFAS1	macrophage_C1	UPP1		
Fibroblast_C2	TM4SF1	macrophage_C1	BNIP3L		
Fibroblast_C2	CREG1	macrophage_C1	PNPLA8		
Fibroblast_C2	MEDAG	macrophage_C1	LDHA		
Fibroblast_C2	KRT8	macrophage_C1	SPHK1		

Fibroblast_C2	CTNNAL1	macrophage_C1	MAP2K1
Fibroblast_C2	TNFAIP6	macrophage_C1	B4GALT5
Fibroblast_C2	SLC39A8	macrophage_C1	MARCKSL1
Fibroblast_C2	MT1E	macrophage_C1	C4orf3
Fibroblast_C2	PLIN2	macrophage_C1	MIR155HG
Fibroblast_C2	C3	macrophage_C1	CLEC4E
Fibroblast_C2	ANGPTL4	macrophage_C1	BZW1
Fibroblast_C2	ATP1B1	macrophage_C2	HNRNPC
Fibroblast_C2	MAP3K8	macrophage_C3	HNRNPA2B1
Fibroblast_C2	SRGAP1	macrophage_C3	VAMP8
Fibroblast_C2	TWIST1	macrophage_C3	TUBA1B
Fibroblast_C2	APOE	macrophage_C3	C1QA
Fibroblast_C2	KRT18	macrophage_C3	C1QB
Fibroblast_C2	TUBB4B	macrophage_C3	CD74
Fibroblast_C2	BAZ1A	macrophage_C3	C1QC
Fibroblast_C2	PNPLA8	macrophage_C3	PRDX1
Fibroblast_C2	SLC25A37	macrophage_C3	CTSD
Fibroblast_C2	SLC12A8	macrophage_C3	GRN
Fibroblast_C2	ELL2	macrophage_C3	GPX1
Fibroblast_C2	CXCL6	macrophage_C3	HLA-DMB
Fibroblast_C2	PPP1CB	macrophage_C3	RGS1
Fibroblast_C2	CLU	macrophage_C3	HLA-DPA1
Fibroblast_C2	EIF4B	macrophage_C3	CST3
Fibroblast_C2	CCL2	macrophage_C3	CAPG
Fibroblast_C2	MYADM	macrophage_C3	FCGRT
Fibroblast_C2	SERTAD1	macrophage_C3	PPT1
Fibroblast_C2	RAB31	macrophage_C3	CTSC
Fibroblast_C2	KLF4	macrophage_C3	HLA-DRB1
Fibroblast_C2	RBBP6	macrophage_C3	LGALS9
Fibroblast_C2	MSANTD3	macrophage_C3	MGST2
Fibroblast_C2	CPXM1	macrophage_C3	YWHAH
Fibroblast_C2	KRT19	macrophage_C3	ACP5
Fibroblast_C3	HNRNPC	macrophage_C3	HLA-DMA
Fibroblast_C3	ID1	macrophage_C3	NPC2
Fibroblast_C4	NDUFA4L2	macrophage_C3	CALM2
Fibroblast_C4	RPLP1	macrophage_C3	ARHGDIB
Fibroblast_C4	CALM3	macrophage_C3	CTSB
Fibroblast_C4	RPL26	macrophage_C3	PLD3
Fibroblast_C4	SRP72	macrophage_C3	RNH1
Fibroblast_C4	N4BP2L2	macrophage_C3	BST2
Fibroblast_C4	RPLP2	macrophage_C3	TREM2
Fibroblast_C4	MTIF3	macrophage_C3	APOE
Fibroblast_C4	DEK	macrophage_C3	FCGR3A
Fibroblast_C4	CHMP5	macrophage_C3	MS4A6A
Fibroblast_C4	MT-ATP6	macrophage_C3	TMED9
Fibroblast_C4	RPS9	macrophage_C3	AKR1A1
Fibroblast_C4	HNRNPR	macrophage_C3	TYMP
Fibroblast_C4	CAST	macrophage_C3	CD81
Fibroblast_C4	VPS29	macrophage_C3	ITM2B
Fibroblast_C4	RPL21	macrophage_C3	HLA-DPB1
Fibroblast_C4	RSL24D1	macrophage_C3	SAMHD1
Fibroblast_C4	RPN2	macrophage_C3	PSAP
Fibroblast_C4	MYEOV2	macrophage_C3	CD14
Fibroblast_C4	ATP1A1	macrophage_C3	SLCO2B1
Fibroblast_C4	RPS19	macrophage_C3	MGST3
Fibroblast_C4	NTMT1	macrophage_C3	CSF1R
Fibroblast_C4	CTNNA1	macrophage_C3	ATP5G3
Fibroblast_C4	RPS17	macrophage_C3	CTSZ

Fibroblast_C4	NUDT5
Fibroblast_C4	PNISR
Fibroblast_C4	PQBP1
Fibroblast_C4	GNL1
Fibroblast_C4	ADM
Fibroblast_C4	RPS27A
Fibroblast_C4	RPS18
Fibroblast_C4	RPS16
Fibroblast_C4	DDX18
Fibroblast_C4	SUPT5H
Fibroblast_C4	ZC3H15
Fibroblast_C4	ACADVL
Fibroblast_C4	DDX17
Fibroblast_C4	TRA2A
Fibroblast_C4	MT-ND3
Fibroblast_C4	C1orf123
Fibroblast_C4	SPCS3
Fibroblast_C4	CLPP
Fibroblast_C4	IRF2BP2
Fibroblast_C4	MT-CO3
Fibroblast_C4	RPL37
Fibroblast_C4	RPS23
Fibroblast_C4	MPC2
Fibroblast_C4	ATP6AP1
Fibroblast_C4	PSMB6
Fibroblast_C4	MT-CO1
Fibroblast_C4	UQCRB

macrophage_C3	TMEM176B
macrophage_C3	TUBB
macrophage_C3	CTSA
macrophage_C3	RANBP1
macrophage_C3	TUBA1A
macrophage_C3	C1orf54
macrophage_C3	VSIG4
macrophage_C3	COX5A
macrophage_C3	PSMB8
macrophage_C3	PSME2
macrophage_C3	LY86
macrophage_C3	TIMP2
macrophage_C3	HLA-DQB1
macrophage_C3	ITGB2
macrophage_C3	LAP3
macrophage_C3	HEXA
macrophage_C3	IER2
macrophage_C3	MNDA
macrophage_C3	LGMN
macrophage_C3	FOS
macrophage_C3	VPS29
macrophage_C3	LAMP1
macrophage_C3	LAPTM5
macrophage_C3	STAB1
macrophage_C3	GSN
macrophage_C3	CD163
macrophage_C3	C1orf162
macrophage_C3	SLC16A3
macrophage_C3	FABP5
macrophage_C3	APOC1
macrophage_C3	CORO1A
macrophage_C3	TXNIP
macrophage_C3	HLA-DRA
macrophage_C3	CD9
macrophage_C3	TMEM176A
macrophage_C3	JUNB
macrophage_C3	ZFP36L2
macrophage_C3	HLA-DQA1
macrophage_C3	MS4A4A
macrophage_C3	FPR3
macrophage_C3	LIPA
macrophage_C3	NDUFA4
macrophage_C3	FAM26F
macrophage_C3	CALR
macrophage_C3	TSPO
macrophage_C3	HLA-DRB5
macrophage_C3	RNASE1
macrophage_C3	TGFBI
macrophage_C3	GPNMB
macrophage_C3	NR4A2
macrophage_C3	FGL2
macrophage_C3	HMGB1
macrophage_C3	STMN1
macrophage_C3	RHOB
macrophage_C3	SGK1
macrophage_C3	PLA2G7
macrophage_C3	HLA-DQA2
macrophage_C3	PLTP

macrophage_C3	JUN
macrophage_C3	DUSP2
macrophage_C3	SDS
macrophage_C3	EGR1
macrophage_C3	FBP1
macrophage_C3	RGS2
macrophage_C3	IGHA1
macrophage_C3	MMP12
macrophage_C3	CCL2
macrophage_C3	SPP1
macrophage_C3	IGLC2
macrophage_C3	IGHG1
macrophage_C4	YTHDC1
macrophage_C4	YTHDF3
macrophage_C4	CTSZ
macrophage_C4	CCNL1
macrophage_C5	FTH1

TableS4 Complex intercellular communication networks between the m6A-mediated immune cells clusters and tumor epithelia cells

No.	source	targe	ligand	receptor	prob	pval	pathv	anno	evidence
1	B cells_C1	Epi	MIF	CD74_CXCR4	0.042849	0	MIF	Secrete	PMID: 29637711; PMID: 24760155
2	B cells_C2	Epi	MIF	CD74_CXCR4	0.04427	0	MIF	Secrete	PMID: 29637711; PMID: 24760155
3	B cells_C3	Epi	MIF	CD74_CXCR4	0.04334	0	MIF	Secrete	PMID: 29637711; PMID: 24760155
4	B cells_C4	Epi	MIF	CD74_CXCR4	0.045032	0	MIF	Secrete	PMID: 29637711; PMID: 24760155
5	Fibroblast_C1	Epi	MIF	CD74_CXCR4	0.040189	0	MIF	Secrete	PMID: 29637711; PMID: 24760155
6	Fibroblast_C2	Epi	MIF	CD74_CXCR4	0.034738	0	MIF	Secrete	PMID: 29637711; PMID: 24760155
7	Fibroblast_C3	Epi	MIF	CD74_CXCR4	0.04057	0	MIF	Secrete	PMID: 29637711; PMID: 24760155
8	Fibroblast_C4	Epi	MIF	CD74_CXCR4	0.041133	0	MIF	Secrete	PMID: 29637711; PMID: 24760155
9	macrophage_C1	Epi	MIF	CD74_CXCR4	0.041475	0	MIF	Secrete	PMID: 29637711; PMID: 24760155
10	macrophage_C2	Epi	MIF	CD74_CXCR4	0.038475	0	MIF	Secrete	PMID: 29637711; PMID: 24760155
11	macrophage_C3	Epi	MIF	CD74_CXCR4	0.038804	0	MIF	Secrete	PMID: 29637711; PMID: 24760155
12	macrophage_C4	Epi	MIF	CD74_CXCR4	0.041384	0	MIF	Secrete	PMID: 29637711; PMID: 24760155
13	macrophage_C5	Epi	MIF	CD74_CXCR4	0.046389	0	MIF	Secrete	PMID: 29637711; PMID: 24760155
14	T cells_C1	Epi	MIF	CD74_CXCR4	0.038105	0	MIF	Secrete	PMID: 29637711; PMID: 24760155
15	T cells_C2	Epi	MIF	CD74_CXCR4	0.038286	0	MIF	Secrete	PMID: 29637711; PMID: 24760155
16	T cells_C3	Epi	MIF	CD74_CXCR4	0.038144	0	MIF	Secrete	PMID: 29637711; PMID: 24760155
17	T cells_C4	Epi	MIF	CD74_CXCR4	0.040714	0	MIF	Secrete	PMID: 29637711; PMID: 24760155
18	T cells_C5	Epi	MIF	CD74_CXCR4	0.032326	0	MIF	Secrete	PMID: 29637711; PMID: 24760155
19	B cells_C1	Epi	MIF	CD74_CD44	0.036584	0	MIF	Secrete	PMID: 29637711; PMID: 26175090
20	B cells_C2	Epi	MIF	CD74_CD44	0.037805	0	MIF	Secrete	PMID: 29637711; PMID: 26175090
21	B cells_C3	Epi	MIF	CD74_CD44	0.037006	0	MIF	Secrete	PMID: 29637711; PMID: 26175090
22	B cells_C4	Epi	MIF	CD74_CD44	0.038461	0	MIF	Secrete	PMID: 29637711; PMID: 26175090
23	Fibroblast_C1	Epi	MIF	CD74_CD44	0.034299	0	MIF	Secrete	PMID: 29637711; PMID: 26175090
24	Fibroblast_C3	Epi	MIF	CD74_CD44	0.034626	0	MIF	Secrete	PMID: 29637711; PMID: 26175090
25	macrophage_C1	Epi	MIF	CD74_CD44	0.035404	0	MIF	Secrete	PMID: 29637711; PMID: 26175090
26	macrophage_C2	Epi	MIF	CD74_CD44	0.032827	0	MIF	Secrete	PMID: 29637711; PMID: 26175090

27	macrophage_C3	Epi	MIF	CD74_CD44	0.03311	0	MIF	Secrete	PMID: 29637711; PMID: 26175090
28	macrophage_C4	Epi	MIF	CD74_CD44	0.035325	0	MIF	Secrete	PMID: 29637711; PMID: 26175090
29	macrophage_C5	Epi	MIF	CD74_CD44	0.039628	0	MIF	Secrete	PMID: 29637711; PMID: 26175090
30	T cells_C1	Epi	MIF	CD74_CD44	0.03251	0	MIF	Secrete	PMID: 29637711; PMID: 26175090
31	T cells_C2	Epi	MIF	CD74_CD44	0.032665	0	MIF	Secrete	PMID: 29637711; PMID: 26175090
32	T cells_C3	Epi	MIF	CD74_CD44	0.032543	0	MIF	Secrete	PMID: 29637711; PMID: 26175090
33	T cells_C4	Epi	MIF	CD74_CD44	0.03475	0	MIF	Secrete	PMID: 29637711; PMID: 26175090
34	B cells_C1	Epi	MDK	NCL	0.00522	0	MK	Secrete	PMID: 28356350
35	B cells_C2	Epi	MDK	NCL	0.004636	0	MK	Secrete	PMID: 28356350
36	B cells_C3	Epi	MDK	NCL	0.005028	0	MK	Secrete	PMID: 28356350
37	B cells_C4	Epi	MDK	NCL	0.005005	0	MK	Secrete	PMID: 28356350
38	Fibroblast_C1	Epi	MDK	NCL	0.004081	0	MK	Secrete	PMID: 28356350
39	Fibroblast_C3	Epi	MDK	NCL	0.003809	0	MK	Secrete	PMID: 28356350
40	macrophage_C1	Epi	MDK	NCL	0.003045	0.05	MK	Secrete	PMID: 28356350
41	macrophage_C2	Epi	MDK	NCL	0.003219	0.02	MK	Secrete	PMID: 28356350
42	macrophage_C4	Epi	MDK	NCL	0.003857	0	MK	Secrete	PMID: 28356350
43	macrophage_C5	Epi	MDK	NCL	0.004204	0.03	MK	Secrete	PMID: 28356350
44	T cells_C1	Epi	MDK	NCL	0.002749	0	MK	Secrete	PMID: 28356350
45	T cells_C4	Epi	MDK	NCL	0.003613	0	MK	Secrete	PMID: 28356350
46	B cells_C1	Epi	LGALS9	PTPRC	0.001366	0	GALEC	Secrete	PMID: 30120235
47	B cells_C2	Epi	LGALS9	PTPRC	0.001356	0	GALEC	Secrete	PMID: 30120235
48	B cells_C3	Epi	LGALS9	PTPRC	0.001336	0	GALEC	Secrete	PMID: 30120235
49	B cells_C4	Epi	LGALS9	PTPRC	0.00122	0	GALEC	Secrete	PMID: 30120235
50	Fibroblast_C1	Epi	LGALS9	PTPRC	0.000853	0	GALEC	Secrete	PMID: 30120235
51	Fibroblast_C3	Epi	LGALS9	PTPRC	0.000799	0.01	GALEC	Secrete	PMID: 30120235
52	Fibroblast_C4	Epi	LGALS9	PTPRC	0.001613	0	GALEC	Secrete	PMID: 30120235
53	macrophage_C4	Epi	LGALS9	PTPRC	0.001111	0	GALEC	Secrete	PMID: 30120235
54	macrophage_C5	Epi	LGALS9	PTPRC	0.001138	0.01	GALEC	Secrete	PMID: 30120235
55	T cells_C1	Epi	LGALS9	PTPRC	0.000905	0	GALEC	Secrete	PMID: 30120235
56	T cells_C2	Epi	LGALS9	PTPRC	0.000719	0	GALEC	Secrete	PMID: 30120235
57	T cells_C4	Epi	LGALS9	PTPRC	0.001105	0	GALEC	Secrete	PMID: 30120235
58	Fibroblast_C2	Epi	ADGRE5	CD55	0.001687	0.04	ADGRE	Cell-Ce	PMID: 11297558
59	macrophage_C1	Epi	ADGRE5	CD55	0.001544	0	ADGRE	Cell-Ce	PMID: 11297558
60	macrophage_C2	Epi	ADGRE5	CD55	0.001437	0.03	ADGRE	Cell-Ce	PMID: 11297558
61	B cells_C1	Epi	APP	CD74	0.011216	0	APP	Cell-Ce	PMID: 19849849
62	B cells_C2	Epi	APP	CD74	0.01085	0	APP	Cell-Ce	PMID: 19849849
63	B cells_C3	Epi	APP	CD74	0.010723	0	APP	Cell-Ce	PMID: 19849849
64	B cells_C4	Epi	APP	CD74	0.010317	0	APP	Cell-Ce	PMID: 19849849
65	Fibroblast_C1	Epi	APP	CD74	0.009888	0	APP	Cell-Ce	PMID: 19849849
66	Fibroblast_C3	Epi	APP	CD74	0.009524	0	APP	Cell-Ce	PMID: 19849849
67	macrophage_C5	Epi	APP	CD74	0.009111	0.01	APP	Cell-Ce	PMID: 19849849
68	T cells_C1	Epi	APP	CD74	0.008661	0	APP	Cell-Ce	PMID: 19849849
69	T cells_C2	Epi	APP	CD74	0.008406	0	APP	Cell-Ce	PMID: 19849849
70	T cells_C3	Epi	APP	CD74	0.007899	0	APP	Cell-Ce	PMID: 19849849
71	T cells_C4	Epi	APP	CD74	0.009232	0	APP	Cell-Ce	PMID: 19849849
72	Fibroblast_C1	Epi	CD99	CD99	0.016443	0.02	CD99	Cell-Ce	KEGG: hsa04514
73	Fibroblast_C3	Epi	CD99	CD99	0.016498	0.04	CD99	Cell-Ce	KEGG: hsa04514
74	Fibroblast_C2	Epi	CLEC2D	KLRB1	0.002957	0	CLEC	Cell-Ce	PMID: 24223577
75	macrophage_C1	Epi	CLEC2D	KLRB1	0.002651	0	CLEC	Cell-Ce	PMID: 24223577
76	B cells_C1	Epi	CLEC2B	KLRB1	0.000529	0	CLEC	Cell-Ce	PMID: 24223577
77	Fibroblast_C1	Epi	CLEC2B	KLRB1	0.00125	0	CLEC	Cell-Ce	PMID: 24223577

78	Fibroblast_C3	Epi	CLEC2B	KLRB1	0.001243	0	CLEC	Cell-C	PMID: 24223577
79	Fibroblast_C4	Epi	CLEC2B	KLRB1	0.002367	0	CLEC	Cell-C	PMID: 24223577
80	macrophage_C1	Epi	CLEC2B	KLRB1	0.001268	0	CLEC	Cell-C	PMID: 24223577
81	macrophage_C2	Epi	CLEC2B	KLRB1	0.000984	0	CLEC	Cell-C	PMID: 24223577
82	macrophage_C3	Epi	CLEC2B	KLRB1	0.000907	0	CLEC	Cell-C	PMID: 24223577
83	macrophage_C4	Epi	CLEC2B	KLRB1	0.000766	0	CLEC	Cell-C	PMID: 24223577
84	T cells_C1	Epi	CLEC2B	KLRB1	0.001103	0	CLEC	Cell-C	PMID: 24223577
85	T cells_C2	Epi	CLEC2B	KLRB1	0.001239	0	CLEC	Cell-C	PMID: 24223577
86	T cells_C3	Epi	CLEC2B	KLRB1	0.000924	0	CLEC	Cell-C	PMID: 24223577
87	T cells_C4	Epi	CLEC2B	KLRB1	0.001117	0	CLEC	Cell-C	PMID: 24223577
88	T cells_C5	Epi	CLEC2B	KLRB1	0.001558	0	CLEC	Cell-C	PMID: 24223577
89	Fibroblast_C1	Epi	CD69	KLRB1	0.000632	0	CLEC	Cell-C	PMID: 24223577
90	Fibroblast_C2	Epi	CD69	KLRB1	0.003227	0	CLEC	Cell-C	PMID: 24223577
91	Fibroblast_C3	Epi	CD69	KLRB1	0.001116	0	CLEC	Cell-C	PMID: 24223577
92	macrophage_C1	Epi	CD69	KLRB1	0.003321	0	CLEC	Cell-C	PMID: 24223577
93	macrophage_C2	Epi	CD69	KLRB1	0.003034	0	CLEC	Cell-C	PMID: 24223577
94	macrophage_C3	Epi	CD69	KLRB1	0.003053	0	CLEC	Cell-C	PMID: 24223577
95	macrophage_C4	Epi	CD69	KLRB1	0.002804	0	CLEC	Cell-C	PMID: 24223577
96	macrophage_C5	Epi	CD69	KLRB1	0.000559	0	CLEC	Cell-C	PMID: 24223577
97	T cells_C1	Epi	CD69	KLRB1	0.001869	0	CLEC	Cell-C	PMID: 24223577
98	T cells_C2	Epi	CD69	KLRB1	0.001967	0	CLEC	Cell-C	PMID: 24223577
99	T cells_C3	Epi	CD69	KLRB1	0.002253	0	CLEC	Cell-C	PMID: 24223577
100	T cells_C4	Epi	CD69	KLRB1	0.001094	0	CLEC	Cell-C	PMID: 24223577
101	T cells_C5	Epi	CD69	KLRB1	0.002728	0	CLEC	Cell-C	PMID: 24223577

TableS5: REAC analysis for Each m6A-mediated subClusters

subtype	p_value	term_size	query_size	intersection_size	precision	recall	term_id	term_name	effective_domain_size	source_order	FDR
mac_C1	0.000	45	86	12	0.14	0.27	REAC:R-HSA-67837	Interleukin-10 signal	10622	1130	0.000
mac_C1	0.000	459	86	22	0.256	0.05	REAC:R-HSA-44914	Signaling by Interleu	10622	2054	0.000
mac_C1	0.000	696	86	25	0.291	0.04	REAC:R-HSA-12802	Cytokine Signaling in	10622	393	0.000
mac_C1	0.000	92	86	9	0.105	0.1	REAC:R-HSA-16816	Toll Like Receptor 3 (	10622	2297	0.000
mac_C1	0.000	96	86	9	0.105	0.09	REAC:R-HSA-16616	MyD88-independent	10622	1372	0.000
mac_C1	0.000	96	86	9	0.105	0.09	REAC:R-HSA-93706	TRIF(TICAM1)-media	10622	2251	0.000
mac_C1	0.000	###	86	27	0.314	0.02	REAC:R-HSA-16824	Innate Immune Syste	10622	1100	0.000
mac_C1	0.000	101	86	9	0.105	0.09	REAC:R-HSA-90207	Interleukin-1 signalin	10622	1129	0.000
mac_C1	0.000	84	86	8	0.093	0.1	REAC:R-HSA-16817	Toll Like Receptor 5 (	10622	2299	0.000
mac_C1	0.000	57	86	7	0.081	0.12	REAC:R-HSA-38010	Chemokine receptors	10622	323	0.000
mac_C1	0.000	84	86	8	0.093	0.1	REAC:R-HSA-16814	Toll Like Receptor 10	10622	2295	0.000
mac_C1	0.000	84	86	8	0.093	0.1	REAC:R-HSA-97587	MyD88 cascade initia	10622	1368	0.000
mac_C1	0.000	89	86	8	0.093	0.09	REAC:R-HSA-97513	TRAF6 mediated indu	10622	2248	0.000
mac_C1	0.000	90	86	8	0.093	0.09	REAC:R-HSA-97515	MyD88 dependent ca	10622	1371	0.000
mac_C1	0.000	91	86	8	0.093	0.09	REAC:R-HSA-16818	Toll Like Receptor 7/	10622	2300	0.000
mac_C1	0.000	94	86	8	0.093	0.09	REAC:R-HSA-16813	Toll Like Receptor 9 (	10622	2301	0.000
mac_C1	0.000	126	86	9	0.105	0.07	REAC:R-HSA-16601	Toll Like Receptor 4 (	10622	2298	0.000
mac_C1	0.000	94	86	8	0.093	0.09	REAC:R-HSA-16605	MyD88:MAL(TIRAP) c	10622	1373	0.000
mac_C1	0.000	94	86	8	0.093	0.09	REAC:R-HSA-16818	Toll Like Receptor TL	10622	2303	0.000
mac_C1	0.000	97	86	8	0.093	0.08	REAC:R-HSA-18143	Toll Like Receptor 2 (	10622	2296	0.000
mac_C1	0.000	97	86	8	0.093	0.08	REAC:R-HSA-16817	Toll Like Receptor TL	10622	2302	0.000
mac_C1	0.000	138	86	9	0.105	0.07	REAC:R-HSA-44665	Interleukin-1 family s	10622	1127	0.000
mac_C1	0.000	152	86	9	0.105	0.06	REAC:R-HSA-16889	Toll-like Receptor Ca	10622	2304	0.000
mac_C1	0.000	476	86	15	0.174	0.03	REAC:R-HSA-67986	Neutrophil degranula	10622	1458	0.000
mac_C1	0.000	63	86	6	0.07	0.1	REAC:R-HSA-45029	MAP kinase activatio	10622	1231	0.000
mac_C1	0.000	71	86	6	0.07	0.08	REAC:R-HSA-44842	Interleukin-17 signal	10622	1134	0.001
mac_C1	0.000	22	86	4	0.047	0.18	REAC:R-HSA-11240	RAF-independent M	10622	1689	0.001
mac_C1	0.000	8	86	3	0.035	0.38	REAC:R-HSA-44870	Interleukin-1 process	10622	1128	0.001
mac_C1	0.000	110	86	7	0.081	0.06	REAC:R-HSA-67858	Interleukin-4 and Int	10622	1148	0.001
mac_C1	0.001	24	86	4	0.047	0.17	REAC:R-HSA-96608	Purinergic signaling i	10622	1674	0.001
mac_C1	0.001	24	86	4	0.047	0.17	REAC:R-HSA-96644	Cell recruitment (pro	10622	304	0.001
mac_C1	0.002	32	86	4	0.047	0.13	REAC:R-HSA-44598	TAK1 activates NFkB	10622	2202	0.002
mac_C1	0.002	14	86	3	0.035	0.21	REAC:R-HSA-71240	Tryptophan catabolis	10622	2385	0.003
mac_C1	0.004	16	86	3	0.035	0.19	REAC:R-HSA-56842	MAP3K8 (TPL2)-depe	10622	1233	0.005
mac_C1	0.012	24	86	3	0.035	0.13	REAC:R-HSA-93354	TRAF6 mediated NF-	10622	2247	0.014
mac_C1	0.012	55	86	4	0.047	0.07	REAC:R-HSA-16864	Nucleotide-binding d	10622	1485	0.014
mac_C1	0.012	195	86	7	0.081	0.04	REAC:R-HSA-37527	Peptide ligand-bindi	10622	1578	0.014
mac_C1	0.012	6	86	2	0.023	0.33	REAC:R-HSA-56606	CLEC7A/inflammasor	10622	259	0.014
mac_C1	0.015	7	86	2	0.023	0.29	REAC:R-HSA-56030	IkBA variant leads to	10622	1068	0.018
mac_C1	0.026	9	86	2	0.023	0.22	REAC:R-HSA-11005	MAPK3 (ERK1) activa	10622	1238	0.029
mac_C1	0.038	125	86	5	0.058	0.04	REAC:R-HSA-11460	Platelet degranulatio	10622	1616	0.042
mac_C1	0.041	39	86	3	0.035	0.08	REAC:R-HSA-88538	Transcriptional Regul	10622	2328	0.045
mac_C1	0.043	130	86	5	0.058	0.04	REAC:R-HSA-76005	Response to elevated	10622	1905	0.045
mac_C1	0.047	13	86	2	0.023	0.15	REAC:R-HSA-20267	ERKs are inactivated	10622	763	0.047
mac_C1	0.047	13	86	2	0.023	0.15	REAC:R-HSA-20956	NF-kB is activated ar	10622	1387	0.047
mac_C1	0.047	42	86	3	0.035	0.07	REAC:R-HSA-56752	Negative regulation o	10622	1435	0.047
mac_C3	0.000	123	98	15	0.153	0.12	REAC:R-HSA-21322	MHC class II antigen	10622	1256	0.000
mac_C3	0.000	###	98	36	0.367	0.03	REAC:R-HSA-16824	Innate Immune Syste	10622	1100	0.000
mac_C3	0.000	476	98	21	0.214	0.04	REAC:R-HSA-67986	Neutrophil degranula	10622	1458	0.000
mac_C3	0.000	799	98	25	0.255	0.03	REAC:R-HSA-12802	Adaptive Immune Sy	10622	111	0.000
mac_C3	0.000	20	98	5	0.051	0.25	REAC:R-HSA-20243	Translocation of ZAP	10622	2355	0.000

mac_C3	0.000	23	98	5	0.051	0.22	REAC:R-HSA-20242	Phosphorylation of C	10622	1601	0.000
mac_C3	0.000	24	98	5	0.051	0.21	REAC:R-HSA-38994	PD-1 signaling	10622	1515	0.000
mac_C3	0.000	696	98	20	0.204	0.03	REAC:R-HSA-12802	Cytokine Signaling in	10622	393	0.001
mac_C3	0.001	35	98	5	0.051	0.14	REAC:R-HSA-20243	Generation of second	10622	952	0.002
mac_C3	0.002	98	98	7	0.071	0.07	REAC:R-HSA-20242	Downstream TCR sig	10622	728	0.004
mac_C3	0.003	193	98	9	0.092	0.05	REAC:R-HSA-91353	Interferon Signaling	10622	1123	0.006
mac_C3	0.003	152	98	8	0.082	0.05	REAC:R-HSA-16889	Toll-like Receptor Ca	10622	2304	0.006
mac_C3	0.004	120	98	7	0.071	0.06	REAC:R-HSA-20240	TCR signaling	10622	2207	0.008
mac_C3	0.007	32	98	4	0.041	0.13	REAC:R-HSA-89640	Plasma lipoprotein cl	10622	1609	0.012
mac_C3	0.009	97	98	6	0.061	0.06	REAC:R-HSA-21737	Binding and Uptake o	10622	207	0.014
mac_C3	0.011	67	98	5	0.051	0.07	REAC:R-HSA-38884	Costimulation by the	10622	379	0.017
mac_C3	0.013	39	98	4	0.041	0.1	REAC:R-HSA-90316	NGF-stimulated trans	10622	1391	0.018
mac_C3	0.027	126	98	6	0.061	0.05	REAC:R-HSA-16601	Toll Like Receptor 4 (	10622	2298	0.033
mac_C3	0.027	83	98	5	0.051	0.06	REAC:R-HSA-12369	ER-Phagosome path	10622	758	0.033
mac_C3	0.027	459	98	12	0.122	0.03	REAC:R-HSA-44914	Signaling by Interleu	10622	2054	0.033
mac_C3	0.029	87	98	5	0.051	0.06	REAC:R-HSA-87730	Interferon gamma sig	10622	1125	0.034
mac_C3	0.048	61	98	4	0.041	0.07	REAC:R-HSA-17362	Classical antibody-m	10622	346	0.048
mac_C3	0.048	61	98	4	0.041	0.07	REAC:R-HSA-19872	Nuclear Events (kinas	10622	1475	0.048
mac_C3	0.048	99	98	5	0.051	0.05	REAC:R-HSA-12369	Antigen processing-C	10622	144	0.048
mac_C3	0.048	101	98	5	0.051	0.05	REAC:R-HSA-97760	Regulation of Compl	10622	1815	0.048
mac_C4	0.019	33	1	1	1	0.03	REAC:R-HSA-56945	Cargo concentration	10622	287	0.032
mac_C4	0.019	35	1	1	1	0.03	REAC:R-HSA-43272	Lysosome Vesicle Bio	10622	1228	0.032
mac_C4	0.019	18	1	1	1	0.06	REAC:R-HSA-20223	Metabolism of Angio	10622	1294	0.032
mac_C4	0.023	68	1	1	1	0.01	REAC:R-HSA-20400	COPII-mediated vesi	10622	263	0.032
mac_C4	0.023	72	1	1	1	0.01	REAC:R-HSA-19999	trans-Golgi Network	10622	2514	0.032
mac_C4	0.024	89	1	1	1	0.01	REAC:R-HSA-29807	Peptide hormone me	10622	1577	0.032
mac_C4	0.035	155	1	1	1	0.01	REAC:R-HSA-19997	ER to Golgi Anterogr	10622	757	0.037
mac_C4	0.037	186	1	1	1	0.01	REAC:R-HSA-94802	Transport to the Golg	10622	2379	0.037
CAF_C1	0.000	57	10	4	0.4	0.07	REAC:R-HSA-33714	HSP90 chaperone cy	10622	1014	0.000
CAF_C1	0.000	14	10	3	0.3	0.21	REAC:R-HSA-33715	Attenuation phase	10622	177	0.000
CAF_C1	0.000	24	10	3	0.3	0.13	REAC:R-HSA-33715	HSF1-dependent tra	10622	1013	0.001
CAF_C1	0.003	87	10	3	0.3	0.03	REAC:R-HSA-33715	Cellular response to	10622	313	0.025
CAF_C1	0.027	44	10	2	0.2	0.05	REAC:R-HSA-96463	Aggrephagy	10622	121	0.042
CAF_C1	0.029	4	10	1	0.1	0.25	REAC:R-HSA-96652	Resistance of ERBB2	10622	1888	0.042
CAF_C1	0.029	80	10	2	0.2	0.03	REAC:R-HSA-96638	Selective autophagy	10622	1986	0.042
CAF_C1	0.029	4	10	1	0.1	0.25	REAC:R-HSA-96652	Resistance of ERBB2	10622	1892	0.042
CAF_C1	0.029	4	10	1	0.1	0.25	REAC:R-HSA-96652	Resistance of ERBB2	10622	1891	0.042
CAF_C1	0.029	4	10	1	0.1	0.25	REAC:R-HSA-96652	Resistance of ERBB2	10622	1890	0.042
CAF_C1	0.029	4	10	1	0.1	0.25	REAC:R-HSA-96652	Resistance of ERBB2	10622	1889	0.042
CAF_C1	0.029	2	10	1	0.1	0.5	REAC:R-HSA-19290	vRNP Assembly	10622	2516	0.042
CAF_C1	0.029	4	10	1	0.1	0.25	REAC:R-HSA-96652	Resistance of ERBB2	10622	1887	0.042
CAF_C1	0.029	79	10	2	0.2	0.03	REAC:R-HSA-88522	The role of GTSE1 in	10622	2285	0.042
CAF_C1	0.029	4	10	1	0.1	0.25	REAC:R-HSA-96652	Resistance of ERBB2	10622	1886	0.042
CAF_C1	0.029	67	10	2	0.2	0.03	REAC:R-HSA-33714	Regulation of HSF1-r	10622	1820	0.042
CAF_C1	0.029	94	10	2	0.2	0.02	REAC:R-HSA-38032	Recruitment of NuMA	10622	1799	0.042
CAF_C1	0.029	4	10	1	0.1	0.25	REAC:R-HSA-96522	Drug-mediated inhib	10622	740	0.042
CAF_C1	0.029	4	10	1	0.1	0.25	REAC:R-HSA-96657	Drug resistance in ER	10622	736	0.042
CAF_C1	0.029	4	10	1	0.1	0.25	REAC:R-HSA-96652	Drug resistance in ER	10622	735	0.042
CAF_C1	0.029	582	10	4	0.4	0.01	REAC:R-HSA-22627	Cellular responses to	10622	317	0.042
CAF_C1	0.029	4	10	1	0.1	0.25	REAC:R-HSA-96652	Resistance of ERBB2	10622	1885	0.042
CAF_C1	0.029	1	10	1	0.1	1	REAC:R-HSA-16833	Viral RNP Complexes	10622	2421	0.042
CAF_C1	0.035	5	10	1	0.1	0.2	REAC:R-HSA-90134	RHOBTB2 GTPase cyc	10622	1712	0.043
CAF_C1	0.036	123	10	2	0.2	0.02	REAC:R-HSA-21322	MHC class II antigen	10622	1256	0.043
CAF_C1	0.036	6	10	1	0.1	0.17	REAC:R-HSA-30004	Scavenging by Class	10622	1982	0.043
CAF_C1	0.036	6	10	1	0.1	0.17	REAC:R-HSA-53364	Uptake and function	10622	2402	0.043
CAF_C1	0.041	134	10	2	0.2	0.01	REAC:R-HSA-16328	Macroautophagy	10622	1270	0.048
CAF_C1	0.046	476	10	3	0.3	0.01	REAC:R-HSA-67986	Neutrophil degranula	10622	1458	0.048
CAF_C1	0.046	152	10	2	0.2	0.01	REAC:R-HSA-16825	Influenza Infection	10622	1081	0.048

CAF_C1	0.046	9	10	1	0.1	0.11	REAC:R-HSA-96342	Constitutive Signaling	10622	375	0.048
CAF_C1	0.050	10	10	1	0.1	0.1	REAC:R-HSA-14741	Tetrahydrobiopterin	10622	2273	0.050
CAF_C2	0.000	110	96	11	0.115	0.1	REAC:R-HSA-67858	Interleukin-4 and Int	10622	1148	0.000
CAF_C2	0.000	45	96	8	0.083	0.18	REAC:R-HSA-67837	Interleukin-10 signal	10622	1130	0.000
CAF_C2	0.000	696	96	23	0.24	0.03	REAC:R-HSA-12802	Cytokine Signaling in	10622	393	0.000
CAF_C2	0.000	459	96	18	0.188	0.04	REAC:R-HSA-44914	Signaling by Interleu	10622	2054	0.000
CAF_C2	0.000	476	96	16	0.167	0.03	REAC:R-HSA-67986	Neutrophil degranula	10622	1458	0.001
CAF_C2	0.002	###	96	24	0.25	0.02	REAC:R-HSA-16824	Innate Immune Syste	10622	1100	0.005
CAF_C2	0.012	57	96	5	0.052	0.09	REAC:R-HSA-38010	Chemokine receptors	10622	323	0.022
CAF_C2	0.022	16	96	3	0.031	0.19	REAC:R-HSA-19363	p75NTR signals via N	10622	2490	0.028
CAF_C2	0.022	39	96	4	0.042	0.1	REAC:R-HSA-88538	Transcriptional Regul	10622	2328	0.028
CAF_C2	0.022	17	96	3	0.031	0.18	REAC:R-HSA-96178	FOXO-mediated tran	10622	847	0.028
CAF_C2	0.029	19	96	3	0.031	0.16	REAC:R-HSA-30004	Scavenging by Class	10622	1980	0.032
CAF_C2	0.032	5	96	2	0.021	0.4	REAC:R-HSA-88494	PTK6 Expression	10622	1560	0.032
CAF_C2	0.032	5	96	2	0.021	0.4	REAC:R-HSA-31349	LRR FLII-interacting p	10622	1192	0.032
CAF_C4	0.000	113	37	15	0.405	0.13	REAC:R-HSA-17993	SRP-dependent cotra	10622	1959	0.000
CAF_C4	0.000	94	37	12	0.324	0.13	REAC:R-HSA-24085	Selenocysteine synth	10622	1988	0.000
CAF_C4	0.000	90	37	12	0.324	0.13	REAC:R-HSA-19282	Viral mRNA Translati	10622	2422	0.000
CAF_C4	0.000	94	37	12	0.324	0.13	REAC:R-HSA-15684	Eukaryotic Translatio	10622	797	0.000
CAF_C4	0.000	94	37	12	0.324	0.13	REAC:R-HSA-72764	Eukaryotic Translatio	10622	799	0.000
CAF_C4	0.000	90	37	12	0.324	0.13	REAC:R-HSA-15690	Peptide chain elonga	10622	1575	0.000
CAF_C4	0.000	96	37	12	0.324	0.13	REAC:R-HSA-97595	Nonsense Mediated	10622	1468	0.000
CAF_C4	0.000	102	37	12	0.324	0.12	REAC:R-HSA-96330	Response of EIF2AK4	10622	1903	0.000
CAF_C4	0.000	102	37	12	0.324	0.12	REAC:R-HSA-72689	Formation of a pool	10622	875	0.000
CAF_C4	0.000	292	37	16	0.432	0.05	REAC:R-HSA-72766	Translation	10622	2343	0.000
CAF_C4	0.000	112	37	12	0.324	0.11	REAC:R-HSA-15682	L13a-mediated trans	10622	1186	0.000
CAF_C4	0.000	113	37	12	0.324	0.11	REAC:R-HSA-72706	GTP hydrolysis and jo	10622	937	0.000
CAF_C4	0.000	116	37	12	0.324	0.1	REAC:R-HSA-92780	Nonsense-Mediated	10622	1469	0.000
CAF_C4	0.000	116	37	12	0.324	0.1	REAC:R-HSA-97595	Nonsense Mediated	10622	1467	0.000
CAF_C4	0.000	117	37	12	0.324	0.1	REAC:R-HSA-24085	Selenoamino acid me	10622	1987	0.000
CAF_C4	0.000	120	37	12	0.324	0.1	REAC:R-HSA-72737	Cap-dependent Tran	10622	284	0.000
CAF_C4	0.000	120	37	12	0.324	0.1	REAC:R-HSA-72613	Eukaryotic Translatio	10622	798	0.000
CAF_C4	0.000	171	37	13	0.351	0.08	REAC:R-HSA-90105	Regulation of expres	10622	1853	0.000
CAF_C4	0.000	133	37	12	0.324	0.09	REAC:R-HSA-16827	Influenza Viral RNA T	10622	1082	0.000
CAF_C4	0.000	152	37	12	0.324	0.08	REAC:R-HSA-16825	Influenza Infection	10622	1081	0.000
CAF_C4	0.000	157	37	12	0.324	0.08	REAC:R-HSA-97110	Cellular response to s	10622	315	0.000
CAF_C4	0.000	217	37	13	0.351	0.06	REAC:R-HSA-37617	Signaling by ROBO re	10622	2090	0.000
CAF_C4	0.000	183	37	12	0.324	0.07	REAC:R-HSA-67912	Major pathway of rRN	10622	1271	0.000
CAF_C4	0.000	193	37	12	0.324	0.06	REAC:R-HSA-88687	rRNA processing in th	10622	2499	0.000
CAF_C4	0.000	203	37	12	0.324	0.06	REAC:R-HSA-72312	rRNA processing	10622	2497	0.000
CAF_C4	0.000	370	37	13	0.351	0.04	REAC:R-HSA-71291	Metabolism of amino	10622	1297	0.000
CAF_C4	0.000	909	37	18	0.486	0.02	REAC:R-HSA-56632	Infectious disease	10622	1079	0.000
CAF_C4	0.000	52	37	7	0.189	0.13	REAC:R-HSA-72695	Formation of the tern	10622	886	0.000
CAF_C4	0.000	59	37	7	0.189	0.12	REAC:R-HSA-72702	Ribosomal scanning	10622	1919	0.000
CAF_C4	0.000	59	37	7	0.189	0.12	REAC:R-HSA-72649	Translation initiation	10622	2344	0.000
CAF_C4	0.000	60	37	7	0.189	0.12	REAC:R-HSA-72662	Activation of the mRN	10622	99	0.000
CAF_C4	0.000	549	37	13	0.351	0.02	REAC:R-HSA-42247	Axon guidance	10622	182	0.000
CAF_C4	0.000	574	37	13	0.351	0.02	REAC:R-HSA-96751	Nervous system deve	10622	1445	0.000
CAF_C4	0.000	582	37	13	0.351	0.02	REAC:R-HSA-22627	Cellular responses to	10622	317	0.000
CAF_C4	0.000	175	37	6	0.162	0.03	REAC:R-HSA-14285	The citric acid (TCA)	10622	2280	0.000
CAF_C4	0.001	125	37	5	0.135	0.04	REAC:R-HSA-16320	Respiratory electron	10622	1901	0.001
CAF_C4	0.005	101	37	4	0.108	0.04	REAC:R-HSA-61110	Respiratory electron	10622	1900	0.005
CAF_C4	0.047	28	37	2	0.054	0.07	REAC:R-HSA-16258	Budding and maturat	10622	240	0.047
CD8+ T c	0.003	909	34	12	0.353	0.01	REAC:R-HSA-56632	Infectious disease	10622	1079	0.017
CD8+ T c	0.003	380	34	8	0.235	0.02	REAC:R-HSA-56632	Diseases of signal tra	10622	712	0.017
CD8+ T c	0.003	###	34	13	0.382	0.01	REAC:R-HSA-16824	Innate Immune Syste	10622	1100	0.017
CD8+ T c	0.004	476	34	8	0.235	0.02	REAC:R-HSA-67986	Neutrophil degranula	10622	1458	0.017
CD8+ T c	0.004	226	34	6	0.176	0.03	REAC:R-HSA-16290	HIV Infection	10622	1005	0.017

CD8+ T c	0.004	148	34	5	0.147	0.03	REAC:R-HSA-45327	Mitotic G1 phase and	10622	1352	0.017
CD8+ T c	0.004	131	34	5	0.147	0.04	REAC:R-HSA-69206	G1/S Transition	10622	914	0.017
CD8+ T c	0.004	74	34	4	0.118	0.05	REAC:R-HSA-17417	APC/C:Cdh1 mediate	10622	24	0.017
CD8+ T c	0.004	77	34	4	0.118	0.05	REAC:R-HSA-17681	Activation of APC/C a	10622	68	0.017
CD8+ T c	0.004	76	34	4	0.118	0.05	REAC:R-HSA-17640	APC/C:Cdc20 mediat	10622	23	0.017
CD8+ T c	0.004	68	34	4	0.118	0.06	REAC:R-HSA-17415	APC/C:Cdc20 mediat	10622	22	0.017
CD8+ T c	0.004	120	34	5	0.147	0.04	REAC:R-HSA-69239	Synthesis of DNA	10622	2159	0.017
CD8+ T c	0.004	79	34	4	0.118	0.05	REAC:R-HSA-88522	The role of GTSE1 in	10622	2285	0.017
CD8+ T c	0.004	81	34	4	0.118	0.05	REAC:R-HSA-90136	Signaling by NOTCH	10622	2074	0.017
CD8+ T c	0.004	32	34	3	0.088	0.09	REAC:R-HSA-70263	Gluconeogenesis	10622	959	0.017
CD8+ T c	0.004	162	34	5	0.147	0.03	REAC:R-HSA-69242	S Phase	10622	1924	0.017
CD8+ T c	0.004	85	34	4	0.118	0.05	REAC:R-HSA-69002	DNA Replication Pre-	10622	414	0.017
CD8+ T c	0.004	88	34	4	0.118	0.05	REAC:R-HSA-45327	Regulation of mitotic	10622	1866	0.017
CD8+ T c	0.004	88	34	4	0.118	0.05	REAC:R-HSA-17414	APC/C-mediated deg	10622	20	0.017
CD8+ T c	0.004	174	34	5	0.147	0.03	REAC:R-HSA-10958	Apoptosis	10622	149	0.017
CD8+ T c	0.004	94	34	4	0.118	0.04	REAC:R-HSA-88781	Transcriptional regula	10622	2334	0.017
CD8+ T c	0.005	98	34	4	0.118	0.04	REAC:R-HSA-20242	Downstream TCR sig	10622	728	0.017
CD8+ T c	0.005	549	34	8	0.235	0.01	REAC:R-HSA-69278	Cell Cycle, Mitotic	10622	301	0.017
CD8+ T c	0.005	101	34	4	0.118	0.04	REAC:R-HSA-90207	Interleukin-1 signalin	10622	1129	0.017
CD8+ T c	0.005	101	34	4	0.118	0.04	REAC:R-HSA-56896	UCH proteinases	10622	2389	0.017
CD8+ T c	0.006	198	34	5	0.147	0.03	REAC:R-HSA-45327	Mitotic G2-G2/M pha	10622	1353	0.017
CD8+ T c	0.006	109	34	4	0.118	0.04	REAC:R-HSA-56963	Nucleotide Excision R	10622	1483	0.017
CD8+ T c	0.006	196	34	5	0.147	0.03	REAC:R-HSA-69275	G2/M Transition	10622	920	0.017
CD8+ T c	0.006	14	34	2	0.059	0.14	REAC:R-HSA-53585	Mismatch repair (MM	10622	1335	0.017
CD8+ T c	0.006	11	34	2	0.059	0.18	REAC:R-HSA-19602	Formation of annular	10622	876	0.017
CD8+ T c	0.006	53	34	3	0.088	0.06	REAC:R-HSA-16991	Regulation of Apopto	10622	1813	0.017
CD8+ T c	0.006	52	34	3	0.088	0.06	REAC:R-HSA-69601	Ubiquitin Mediated D	10622	2393	0.017
CD8+ T c	0.006	52	34	3	0.088	0.06	REAC:R-HSA-75815	Ubiquitin-dependent	10622	2394	0.017
CD8+ T c	0.006	60	34	3	0.088	0.05	REAC:R-HSA-56107	GLI3 is processed to	10622	928	0.017
CD8+ T c	0.006	55	34	3	0.088	0.05	REAC:R-HSA-88540	FBXL7 down-regulate	10622	815	0.017
CD8+ T c	0.006	52	34	3	0.088	0.06	REAC:R-HSA-18053	Vpu mediated degra	10622	2438	0.017
CD8+ T c	0.006	12	34	2	0.059	0.17	REAC:R-HSA-19087	Gap junction degrada	10622	943	0.017
CD8+ T c	0.006	12	34	2	0.059	0.17	REAC:R-HSA-33715	HSF1 activation	10622	1012	0.017
CD8+ T c	0.006	52	34	3	0.088	0.06	REAC:R-HSA-69610	p53-Independent DN	10622	2484	0.017
CD8+ T c	0.006	53	34	3	0.088	0.06	REAC:R-HSA-18058	Vif-mediated degrad	10622	2419	0.017
CD8+ T c	0.006	54	34	3	0.088	0.06	REAC:R-HSA-89418	Regulation of RUNX3	10622	1835	0.017
CD8+ T c	0.006	50	34	3	0.088	0.06	REAC:R-HSA-21173	Regulation of activat	10622	1847	0.017
CD8+ T c	0.006	55	34	3	0.088	0.05	REAC:R-HSA-17411	SCF-beta-TrCP medi	10622	1934	0.017
CD8+ T c	0.006	60	34	3	0.088	0.05	REAC:R-HSA-18757	SCF(Skp2)-mediated	10622	1933	0.017
CD8+ T c	0.006	52	34	3	0.088	0.06	REAC:R-HSA-34942	Autodegradation of t	10622	179	0.017
CD8+ T c	0.006	50	34	3	0.088	0.06	REAC:R-HSA-12369	Cross-presentation o	10622	384	0.017
CD8+ T c	0.006	13	34	2	0.059	0.15	REAC:R-HSA-14034	Apoptosis induced D	10622	150	0.017
CD8+ T c	0.006	57	34	3	0.088	0.05	REAC:R-HSA-69541	Stabilization of p53	10622	2135	0.017
CD8+ T c	0.006	14	34	2	0.059	0.14	REAC:R-HSA-69166	Removal of the Flap	10622	1879	0.017
CD8+ T c	0.006	120	34	4	0.118	0.03	REAC:R-HSA-20240	TCR signaling	10622	2207	0.017
CD8+ T c	0.006	60	34	3	0.088	0.05	REAC:R-HSA-56077	Dectin-1 mediated n	10622	424	0.017
CD8+ T c	0.006	55	34	3	0.088	0.05	REAC:R-HSA-46412	Degradation of AXIN	10622	665	0.017
CD8+ T c	0.006	51	34	3	0.088	0.06	REAC:R-HSA-35056	Regulation of ornithi	10622	1868	0.017
CD8+ T c	0.006	57	34	3	0.088	0.05	REAC:R-HSA-46412	Degradation of DVL	10622	666	0.017
CD8+ T c	0.006	60	34	3	0.088	0.05	REAC:R-HSA-56107	Degradation of GLI1	10622	668	0.017
CD8+ T c	0.006	54	34	3	0.088	0.06	REAC:R-HSA-45040	AUF1 (hnRNP D0) bin	10622	34	0.017
CD8+ T c	0.006	60	34	3	0.088	0.05	REAC:R-HSA-56107	Degradation of GLI2	10622	669	0.017
CD8+ T c	0.006	52	34	3	0.088	0.06	REAC:R-HSA-69613	p53-Independent G1	10622	2485	0.017
CD8+ T c	0.006	59	34	3	0.088	0.05	REAC:R-HSA-53873	Hh mutants abrogate	10622	1024	0.017
CD8+ T c	0.006	59	34	3	0.088	0.05	REAC:R-HSA-68827	CDT1 association wit	10622	255	0.017
CD8+ T c	0.006	58	34	3	0.088	0.05	REAC:R-HSA-35120	Metabolism of polya	10622	1309	0.017
CD8+ T c	0.006	14	34	2	0.059	0.14	REAC:R-HSA-53586	Mismatch repair (MM	10622	1334	0.017
CD8+ T c	0.006	53	34	3	0.088	0.06	REAC:R-HSA-96043	Negative regulation o	10622	1438	0.017

CD8+ T c	0.006	125	34	4	0.118	0.03	REAC:R-HSA-16290	Host Interactions of H	10622	1034	0.017
CD8+ T c	0.006	59	34	3	0.088	0.05	REAC:R-HSA-56765	NIK-->noncanonical	10622	1392	0.017
CD8+ T c	0.006	56	34	3	0.088	0.05	REAC:R-HSA-53627	Hh mutants are degr	10622	1025	0.017
CD8+ T c	0.006	138	34	4	0.118	0.03	REAC:R-HSA-44665	Interleukin-1 family s	10622	1127	0.017
CD8+ T c	0.006	61	34	3	0.088	0.05	REAC:R-HSA-56788	Defective CFTR cause	10622	470	0.017
CD8+ T c	0.007	15	34	2	0.059	0.13	REAC:R-HSA-53585	Mismatch Repair	10622	1333	0.017
CD8+ T c	0.007	15	34	2	0.059	0.13	REAC:R-HSA-69183	Processive synthesis	10622	1653	0.017
CD8+ T c	0.007	63	34	3	0.088	0.05	REAC:R-HSA-67822	Gap-filling DNA repa	10622	947	0.017
CD8+ T c	0.007	64	34	3	0.088	0.05	REAC:R-HSA-46088	Asymmetric localizati	10622	173	0.017
CD8+ T c	0.007	64	34	3	0.088	0.05	REAC:R-HSA-67821	Dual incision in TC-N	10622	742	0.017
CD8+ T c	0.007	64	34	3	0.088	0.05	REAC:R-HSA-17408	Autodegradation of	10622	178	0.017
CD8+ T c	0.007	16	34	2	0.059	0.13	REAC:R-HSA-11031	Translesion synthesis	10622	2352	0.017
CD8+ T c	0.007	66	34	3	0.088	0.05	REAC:R-HSA-69580	p53-Dependent G1/S	10622	2483	0.017
CD8+ T c	0.007	66	34	3	0.088	0.05	REAC:R-HSA-69563	p53-Dependent G1 D	10622	2482	0.017
CD8+ T c	0.007	65	34	3	0.088	0.05	REAC:R-HSA-53583	Hedgehog ligand bio	10622	1017	0.017
CD8+ T c	0.007	66	34	3	0.088	0.05	REAC:R-HSA-12341	Oxygen-dependent	10622	1510	0.017
CD8+ T c	0.007	68	34	3	0.088	0.04	REAC:R-HSA-69615	G1/S DNA Damage C	10622	913	0.017
CD8+ T c	0.007	17	34	2	0.059	0.12	REAC:R-HSA-17443	Removal of the Flap	10622	1880	0.017
CD8+ T c	0.007	67	34	3	0.088	0.04	REAC:R-HSA-70171	Glycolysis	10622	981	0.017
CD8+ T c	0.007	68	34	3	0.088	0.04	REAC:R-HSA-68867	Assembly of the pre-	10622	170	0.017
CD8+ T c	0.007	67	34	3	0.088	0.04	REAC:R-HSA-56584	Regulation of RAS by	10622	1832	0.017
CD8+ T c	0.007	67	34	3	0.088	0.04	REAC:R-HSA-11690	Activation of NF-kap	10622	81	0.017
CD8+ T c	0.007	17	34	2	0.059	0.12	REAC:R-HSA-56561	Translesion synthesis	10622	2350	0.017
CD8+ T c	0.007	17	34	2	0.059	0.12	REAC:R-HSA-56558	Translesion synthesis	10622	2351	0.017
CD8+ T c	0.007	69	34	3	0.088	0.04	REAC:R-HSA-89487	Regulation of PTEN s	10622	1831	0.017
CD8+ T c	0.007	264	34	5	0.147	0.02	REAC:R-HSA-56730	RAF/MAP kinase casc	10622	1690	0.017
CD8+ T c	0.008	18	34	2	0.059	0.11	REAC:R-HSA-44635	Cell-extracellular ma	10622	309	0.017
CD8+ T c	0.008	71	34	3	0.088	0.04	REAC:R-HSA-68949	Orc1 removal from c	10622	1499	0.017
CD8+ T c	0.008	71	34	3	0.088	0.04	REAC:R-HSA-89399	Regulation of RUNX2	10622	1834	0.017
CD8+ T c	0.008	269	34	5	0.147	0.02	REAC:R-HSA-56849	MAPK1/MAPK3 signa	10622	1237	0.017
CD8+ T c	0.008	74	34	3	0.088	0.04	REAC:R-HSA-17941	APC:Cdc20 mediated	10622	25	0.017
CD8+ T c	0.008	19	34	2	0.059	0.11	REAC:R-HSA-11032	Translesion Synthesis	10622	2349	0.017
CD8+ T c	0.008	73	34	3	0.088	0.04	REAC:R-HSA-69017	CDK-mediated phos	10622	254	0.017
CD8+ T c	0.008	407	34	6	0.176	0.01	REAC:R-HSA-68886	M Phase	10622	1230	0.017
CD8+ T c	0.008	73	34	3	0.088	0.04	REAC:R-HSA-17418	Cdc20:Phospho-APC	10622	298	0.017
CD8+ T c	0.008	19	34	2	0.059	0.11	REAC:R-HSA-17441	Processive synthesis	10622	1652	0.017
CD8+ T c	0.008	75	34	3	0.088	0.04	REAC:R-HSA-12341	Cellular response to	10622	314	0.018
CD8+ T c	0.008	20	34	2	0.059	0.1	REAC:R-HSA-69186	Lagging Strand Synth	10622	1195	0.018
CD8+ T c	0.009	77	34	3	0.088	0.04	REAC:R-HSA-67818	Transcription-Couple	10622	2323	0.018
CD8+ T c	0.009	77	34	3	0.088	0.04	REAC:R-HSA-56190	ABC transporter diso	10622	4	0.018
CD8+ T c	0.009	168	34	4	0.118	0.02	REAC:R-HSA-69481	G2/M Checkpoints	10622	917	0.018
CD8+ T c	0.009	21	34	2	0.059	0.1	REAC:R-HSA-56518	PCNA-Dependent Lo	10622	1513	0.019
CD8+ T c	0.009	80	34	3	0.088	0.04	REAC:R-HSA-11683	Downstream signalin	10622	730	0.019
CD8+ T c	0.009	81	34	3	0.088	0.04	REAC:R-HSA-17640	Regulation of APC/C	10622	1812	0.019
CD8+ T c	0.010	83	34	3	0.088	0.04	REAC:R-HSA-12369	ER-Phagosome path	10622	758	0.019
CD8+ T c	0.010	83	34	3	0.088	0.04	REAC:R-HSA-19525	Degradation of beta-	10622	670	0.019
CD8+ T c	0.010	83	34	3	0.088	0.04	REAC:R-HSA-69202	Cyclin E associated e	10622	389	0.019
CD8+ T c	0.010	178	34	4	0.118	0.02	REAC:R-HSA-72163	mRNA Splicing - Ma	10622	2472	0.019
CD8+ T c	0.010	84	34	3	0.088	0.04	REAC:R-HSA-56963	Global Genome Nucl	10622	954	0.019
CD8+ T c	0.010	23	34	2	0.059	0.09	REAC:R-HSA-96376	Response of Mtb to	10622	1904	0.019
CD8+ T c	0.010	85	34	3	0.088	0.04	REAC:R-HSA-70326	Glucose metabolism	10622	960	0.019
CD8+ T c	0.010	85	34	3	0.088	0.04	REAC:R-HSA-56326	Hedgehog 'on' state	10622	1016	0.019
CD8+ T c	0.010	85	34	3	0.088	0.04	REAC:R-HSA-69656	Cyclin A:Cdk2-associ	10622	387	0.019
CD8+ T c	0.010	86	34	3	0.088	0.03	REAC:R-HSA-45053	Regulation of mRNA	10622	1865	0.019
CD8+ T c	0.011	25	34	2	0.059	0.08	REAC:R-HSA-56963	Gap-filling DNA repa	10622	946	0.019
CD8+ T c	0.011	186	34	4	0.118	0.02	REAC:R-HSA-72172	mRNA Splicing	10622	2471	0.019
CD8+ T c	0.011	25	34	2	0.059	0.08	REAC:R-HSA-11037	Resolution of AP site	10622	1893	0.019
CD8+ T c	0.011	89	34	3	0.088	0.03	REAC:R-HSA-56871	MAPK6/MAPK4 signa	10622	1239	0.019

CD8+ T c	0.011	308	34	5	0.147	0.02	REAC:R-HSA-56830	MAPK family signalin	10622	1234	0.019
CD8+ T c	0.011	25	34	2	0.059	0.08	REAC:R-HSA-68035	FGFR2 alternative spl	10622	828	0.019
CD8+ T c	0.011	188	34	4	0.118	0.02	REAC:R-HSA-24678	Separation of Sister C	10622	2000	0.020
CD8+ T c	0.011	91	34	3	0.088	0.03	REAC:R-HSA-69052	Switching of origins t	10622	2148	0.020
CD8+ T c	0.011	459	34	6	0.176	0.01	REAC:R-HSA-44914	Signaling by Interleu	10622	2054	0.020
CD8+ T c	0.012	92	34	3	0.088	0.03	REAC:R-HSA-40864	PCP/CE pathway	10622	1514	0.020
CD8+ T c	0.012	94	34	3	0.088	0.03	REAC:R-HSA-44200	VEGFA-VEGFR2 Path	10622	2407	0.021
CD8+ T c	0.012	27	34	2	0.059	0.07	REAC:R-HSA-96354	Infection with Mycob	10622	1078	0.021
CD8+ T c	0.013	28	34	2	0.059	0.07	REAC:R-HSA-56018	PIWI-interacting RNA	10622	1540	0.022
CD8+ T c	0.013	97	34	3	0.088	0.03	REAC:R-HSA-56077	CLEC7A (Dectin-1) si	10622	258	0.022
CD8+ T c	0.014	99	34	3	0.088	0.03	REAC:R-HSA-12369	Antigen processing-(	10622	144	0.022
CD8+ T c	0.014	100	34	3	0.088	0.03	REAC:R-HSA-56685	TNFR2 non-canonica	10622	2230	0.023
CD8+ T c	0.014	486	34	6	0.176	0.01	REAC:R-HSA-90069	Signaling by Recepto	10622	2091	0.023
CD8+ T c	0.014	30	34	2	0.059	0.07	REAC:R-HSA-11031	Recognition of DNA	10622	1797	0.023
CD8+ T c	0.015	103	34	3	0.088	0.03	REAC:R-HSA-38255	ABC-family proteins	10622	6	0.023
CD8+ T c	0.015	104	34	3	0.088	0.03	REAC:R-HSA-19413	Signaling by VEGF	10622	2101	0.023
CD8+ T c	0.015	31	34	2	0.059	0.06	REAC:R-HSA-44509	Interaction between	10622	1115	0.023
CD8+ T c	0.015	32	34	2	0.059	0.06	REAC:R-HSA-56264	RHO GTPases activat	10622	1703	0.023
CD8+ T c	0.015	32	34	2	0.059	0.06	REAC:R-HSA-41899	Adherens junctions ir	10622	115	0.023
CD8+ T c	0.015	32	34	2	0.059	0.06	REAC:R-HSA-56561	Termination of transl	10622	2272	0.023
CD8+ T c	0.015	32	34	2	0.059	0.06	REAC:R-HSA-69190	DNA strand elongati	10622	417	0.023
CD8+ T c	0.017	34	34	2	0.059	0.06	REAC:R-HSA-17441	Telomere C-strand (L	10622	2266	0.026
CD8+ T c	0.018	112	34	3	0.088	0.03	REAC:R-HSA-15757	Telomere Maintenan	10622	2269	0.026
CD8+ T c	0.018	113	34	3	0.088	0.03	REAC:R-HSA-56107	Hedgehog 'off' state	10622	1015	0.026
CD8+ T c	0.018	35	34	2	0.059	0.06	REAC:R-HSA-56632	RHO GTPases Activat	10622	1701	0.026
CD8+ T c	0.018	35	34	2	0.059	0.06	REAC:R-HSA-68029	Signaling by high-kir	10622	2109	0.026
CD8+ T c	0.018	696	34	7	0.206	0.01	REAC:R-HSA-12802	Cytokine Signaling in	10622	393	0.026
CD8+ T c	0.018	228	34	4	0.118	0.02	REAC:R-HSA-68882	Mitotic Anaphase	10622	1351	0.026
CD8+ T c	0.018	229	34	4	0.118	0.02	REAC:R-HSA-25553	Mitotic Metaphase ar	10622	1354	0.026
CD8+ T c	0.018	36	34	2	0.059	0.06	REAC:R-HSA-73933	Resolution of Abasic	10622	1895	0.026
CD8+ T c	0.018	116	34	3	0.088	0.03	REAC:R-HSA-20294	Regulation of actin d	10622	1846	0.026
CD8+ T c	0.019	233	34	4	0.118	0.02	REAC:R-HSA-15711	Signaling by NOTCH	10622	2065	0.027
CD8+ T c	0.019	118	34	3	0.088	0.03	REAC:R-HSA-88781	Transcriptional regula	10622	2333	0.027
CD8+ T c	0.019	2	34	1	0.029	0.5	REAC:R-HSA-19290	vRNP Assembly	10622	2516	0.027
CD8+ T c	0.019	2	34	1	0.029	0.5	REAC:R-HSA-96366	Manipulation of host	10622	1272	0.027
CD8+ T c	0.020	38	34	2	0.059	0.05	REAC:R-HSA-89505	Gene and protein exp	10622	950	0.027
CD8+ T c	0.020	237	34	4	0.118	0.02	REAC:R-HSA-72203	Processing of Cappe	10622	1647	0.027
CD8+ T c	0.020	39	34	2	0.059	0.05	REAC:R-HSA-56741	MAP2K and MAPK ac	10622	1232	0.027
CD8+ T c	0.020	39	34	2	0.059	0.05	REAC:R-HSA-11031	Translesion synthesis	10622	2353	0.027
CD8+ T c	0.021	40	34	2	0.059	0.05	REAC:R-HSA-96562	Signaling by RAF1 m	10622	2087	0.028
CD8+ T c	0.021	40	34	2	0.059	0.05	REAC:R-HSA-39286	EPHB-mediated forw	10622	755	0.028
CD8+ T c	0.021	549	34	6	0.176	0.01	REAC:R-HSA-42247	Axon guidance	10622	182	0.028
CD8+ T c	0.022	41	34	2	0.059	0.05	REAC:R-HSA-56964	Dual Incision in GG-N	10622	741	0.029
CD8+ T c	0.023	130	34	3	0.088	0.02	REAC:R-HSA-89392	RUNX1 regulates trar	10622	1771	0.031
CD8+ T c	0.025	45	34	2	0.059	0.04	REAC:R-HSA-96499	Signaling downstrear	10622	2117	0.032
CD8+ T c	0.025	45	34	2	0.059	0.04	REAC:R-HSA-68029	Signaling by modera	10622	2113	0.032
CD8+ T c	0.025	45	34	2	0.059	0.04	REAC:R-HSA-68029	Signaling by RAS mu	10622	2088	0.032
CD8+ T c	0.025	574	34	6	0.176	0.01	REAC:R-HSA-96751	Nervous system deve	10622	1445	0.032
CD8+ T c	0.025	45	34	2	0.059	0.04	REAC:R-HSA-68029	Paradoxical activatio	10622	1568	0.032
CD8+ T c	0.025	136	34	3	0.088	0.02	REAC:R-HSA-56214	C-type lectin recepto	10622	243	0.032
CD8+ T c	0.025	136	34	3	0.088	0.02	REAC:R-HSA-28718	FCERI mediated NF-k	10622	819	0.032
CD8+ T c	0.026	138	34	3	0.088	0.02	REAC:R-HSA-68070	PTEN Regulation	10622	1557	0.033
CD8+ T c	0.027	139	34	3	0.088	0.02	REAC:R-HSA-73886	Chromosome Mainte	10622	333	0.033
CD8+ T c	0.027	47	34	2	0.059	0.04	REAC:R-HSA-90205	Interleukin-12 signal	10622	1132	0.033
CD8+ T c	0.027	140	34	3	0.088	0.02	REAC:R-HSA-20294	Fcgamma receptor (F	10622	862	0.033
CD8+ T c	0.027	48	34	2	0.059	0.04	REAC:R-HSA-43723	Recycling pathway of	10622	1803	0.033
CD8+ T c	0.027	48	34	2	0.059	0.04	REAC:R-HSA-73893	DNA Damage Bypass	10622	406	0.033
CD8+ T c	0.028	49	34	2	0.059	0.04	REAC:R-HSA-19082	Gap junction trafficki	10622	944	0.034

CD8+ T c	0.028	143	34	3	0.088	0.02	REAC:R-HSA-38584	Beta-catenin indeper	10622	201	0.034
CD8+ T c	0.028	49	34	2	0.059	0.04	REAC:R-HSA-39286	EPH-ephrin mediatec	10622	753	0.034
CD8+ T c	0.029	50	34	2	0.059	0.04	REAC:R-HSA-15785	Gap junction trafficki	10622	945	0.034
CD8+ T c	0.029	147	34	3	0.088	0.02	REAC:R-HSA-16258	HIV Life Cycle	10622	1006	0.034
CD8+ T c	0.030	51	34	2	0.059	0.04	REAC:R-HSA-18078	Extension of Telomer	10622	811	0.034
CD8+ T c	0.030	51	34	2	0.059	0.04	REAC:R-HSA-75153	Apoptotic execution	10622	153	0.034
CD8+ T c	0.030	148	34	3	0.088	0.02	REAC:R-HSA-90185	Estrogen-dependent	10622	793	0.034
CD8+ T c	0.030	149	34	3	0.088	0.02	REAC:R-HSA-53583	Signaling by Hedgeh	10622	2051	0.034
CD8+ T c	0.031	4	34	1	0.029	0.25	REAC:R-HSA-96652	Resistance of ERBB2	10622	1888	0.034
CD8+ T c	0.031	4	34	1	0.029	0.25	REAC:R-HSA-96652	Resistance of ERBB2	10622	1892	0.034
CD8+ T c	0.031	4	34	1	0.029	0.25	REAC:R-HSA-96652	Resistance of ERBB2	10622	1891	0.034
CD8+ T c	0.031	4	34	1	0.029	0.25	REAC:R-HSA-96522	Drug-mediated inhib	10622	740	0.034
CD8+ T c	0.031	4	34	1	0.029	0.25	REAC:R-HSA-96652	Resistance of ERBB2	10622	1889	0.034
CD8+ T c	0.031	4	34	1	0.029	0.25	REAC:R-HSA-96652	Drug resistance in ER	10622	735	0.034
CD8+ T c	0.031	4	34	1	0.029	0.25	REAC:R-HSA-96657	Drug resistance in ER	10622	736	0.034
CD8+ T c	0.031	4	34	1	0.029	0.25	REAC:R-HSA-96652	Resistance of ERBB2	10622	1885	0.034
CD8+ T c	0.031	4	34	1	0.029	0.25	REAC:R-HSA-96652	Resistance of ERBB2	10622	1886	0.034
CD8+ T c	0.031	4	34	1	0.029	0.25	REAC:R-HSA-96652	Resistance of ERBB2	10622	1887	0.034
CD8+ T c	0.031	4	34	1	0.029	0.25	REAC:R-HSA-96652	Resistance of ERBB2	10622	1890	0.034
CD8+ T c	0.032	290	34	4	0.118	0.01	REAC:R-HSA-69620	Cell Cycle Checkpoin	10622	300	0.035
CD8+ T c	0.034	57	34	2	0.059	0.04	REAC:R-HSA-44711	Interleukin-12 family	10622	1131	0.037
CD8+ T c	0.034	297	34	4	0.118	0.01	REAC:R-HSA-56884	Deubiquitination	10622	682	0.037
CD8+ T c	0.035	163	34	3	0.088	0.02	REAC:R-HSA-98370	Signaling by the B Ce	10622	2116	0.038
CD8+ T c	0.037	5	34	1	0.029	0.2	REAC:R-HSA-90134	RHOBTB2 GTPase cyc	10622	1712	0.040
CD8+ T c	0.037	167	34	3	0.088	0.02	REAC:R-HSA-56935	DNA Double-Strand	10622	411	0.040
CD8+ T c	0.039	171	34	3	0.088	0.02	REAC:R-HSA-90105	Regulation of express	10622	1853	0.042
CD8+ T c	0.040	173	34	3	0.088	0.02	REAC:R-HSA-56191	Disorders of transme	10622	717	0.042
CD8+ T c	0.040	64	34	2	0.059	0.03	REAC:R-HSA-56547	Signaling by FGFR2	10622	2036	0.042
CD8+ T c	0.040	64	34	2	0.059	0.03	REAC:R-HSA-42127	Cell-cell junction org	10622	308	0.042
CD8+ T c	0.040	64	34	2	0.059	0.03	REAC:R-HSA-68029	Signaling by BRAF ar	10622	2018	0.042
CD8+ T c	0.043	6	34	1	0.029	0.17	REAC:R-HSA-53364	Uptake and function	10622	2402	0.044
CD8+ T c	0.043	6	34	1	0.029	0.17	REAC:R-HSA-30004	Scavenging by Class	10622	1982	0.044
CD8+ T c	0.043	67	34	2	0.059	0.03	REAC:R-HSA-56859	HDR through Homol	10622	1000	0.045
CD8+ T c	0.044	68	34	2	0.059	0.03	REAC:R-HSA-38025	Loss of Nlp from mit	10622	1222	0.045
CD8+ T c	0.044	68	34	2	0.059	0.03	REAC:R-HSA-38028	Loss of proteins requ	10622	1225	0.045
CD8+ T c	0.044	182	34	3	0.088	0.02	REAC:R-HSA-24542	Fc epsilon receptor (	10622	861	0.045
CD8+ T c	0.047	71	34	2	0.059	0.03	REAC:R-HSA-88545	AURKA Activation by	10622	35	0.048
CD8+ T c	0.048	7	34	1	0.029	0.14	REAC:R-HSA-16484	2-LTR circle formatio	10622	1	0.048
CD8+ T c	0.048	72	34	2	0.059	0.03	REAC:R-HSA-14451	Translocation of SLC	10622	2354	0.048
CD8+ T c	0.005	8	2	1	0.5	0.13	REAC:R-HSA-73942	DNA Damage Revers	10622	408	0.010
CD8+ T c	0.005	7	2	1	0.5	0.14	REAC:R-HSA-73943	Reversal of alkylation	10622	1914	0.010
CD8+ T c	0.007	17	2	1	0.5	0.06	REAC:R-HSA-45051	Tristetraprolin (TTP, Z	10622	2383	0.010
CD8+ T c	0.028	86	2	1	0.5	0.01	REAC:R-HSA-45053	Regulation of mRNA	10622	1865	0.028
NK cells_(	0.002	7	1	1	1	0.14	REAC:R-HSA-73943	Reversal of alkylation	10622	1914	0.002
NK cells_(	0.002	8	1	1	1	0.13	REAC:R-HSA-73942	DNA Damage Revers	10622	408	0.002
Regulator	0.000	5	4	2	0.5	0.4	REAC:R-HSA-90134	RHOBTB2 GTPase cyc	10622	1712	0.003
Regulator	0.000	6	4	2	0.5	0.33	REAC:R-HSA-53364	Uptake and function	10622	2402	0.003
Regulator	0.000	16	4	2	0.5	0.13	REAC:R-HSA-39995	Sema3A PAK depend	10622	1989	0.003
Regulator	0.000	16	4	2	0.5	0.13	REAC:R-HSA-97065	RHOBTB GTPase Cyc	10622	1710	0.003
Regulator	0.000	16	4	2	0.5	0.13	REAC:R-HSA-90129	RHO GTPase cycle	10622	1696	0.003
Regulator	0.000	14	4	2	0.5	0.14	REAC:R-HSA-33715	Attenuation phase	10622	177	0.003
Regulator	0.000	12	4	2	0.5	0.17	REAC:R-HSA-33715	HSF1 activation	10622	1012	0.003
Regulator	0.000	22	4	2	0.5	0.09	REAC:R-HSA-96138	Chaperone Mediated	10622	321	0.004
Regulator	0.000	24	4	2	0.5	0.08	REAC:R-HSA-33715	HSF1-dependent tra	10622	1013	0.004
Regulator	0.000	25	4	2	0.5	0.08	REAC:R-HSA-53395	Uptake and actions c	10622	2400	0.004
Regulator	0.002	57	4	2	0.5	0.04	REAC:R-HSA-33714	HSP90 chaperone cy	10622	1014	0.015
Regulator	0.002	64	4	2	0.5	0.03	REAC:R-HSA-37375	Semaphorin interacti	10622	1993	0.015
Regulator	0.003	476	4	3	0.75	0.01	REAC:R-HSA-67986	Neutrophil degranula	10622	1458	0.015

Regulator	0.003	79	4	2	0.5	0.03	REAC:R-HSA-96791	Potential therapeutic	10622	1634	0.015
Regulator	0.003	79	4	2	0.5	0.03	REAC:R-HSA-88522	The role of GTSE1 in	10622	2285	0.015
Regulator	0.003	87	4	2	0.5	0.02	REAC:R-HSA-33715	Cellular response to	10622	313	0.015
Regulator	0.005	116	4	2	0.5	0.02	REAC:R-HSA-20294	Regulation of actin d	10622	1846	0.015
Regulator	0.005	2	4	1	0.25	0.5	REAC:R-HSA-19290	vRNP Assembly	10622	2516	0.015
Regulator	0.005	4	4	1	0.25	0.25	REAC:R-HSA-96652	Resistance of ERBB2	10622	1892	0.015
Regulator	0.005	4	4	1	0.25	0.25	REAC:R-HSA-96652	Resistance of ERBB2	10622	1888	0.015
Regulator	0.005	4	4	1	0.25	0.25	REAC:R-HSA-96652	Resistance of ERBB2	10622	1891	0.015
Regulator	0.005	4	4	1	0.25	0.25	REAC:R-HSA-96652	Resistance of ERBB2	10622	1889	0.015
Regulator	0.005	4	4	1	0.25	0.25	REAC:R-HSA-96652	Resistance of ERBB2	10622	1887	0.015
Regulator	0.005	148	4	2	0.5	0.01	REAC:R-HSA-90185	Estrogen-dependent	10622	793	0.015
Regulator	0.005	4	4	1	0.25	0.25	REAC:R-HSA-96652	Resistance of ERBB2	10622	1890	0.015
Regulator	0.005	140	4	2	0.5	0.01	REAC:R-HSA-20294	Fcgamma receptor (F	10622	862	0.015
Regulator	0.005	4	4	1	0.25	0.25	REAC:R-HSA-96652	Resistance of ERBB2	10622	1886	0.015
Regulator	0.005	4	4	1	0.25	0.25	REAC:R-HSA-96657	Drug resistance in ER	10622	736	0.015
Regulator	0.005	4	4	1	0.25	0.25	REAC:R-HSA-96522	Drug-mediated inhib	10622	740	0.015
Regulator	0.005	144	4	2	0.5	0.01	REAC:R-HSA-96795	SARS-CoV Infections	10622	1928	0.015
Regulator	0.005	4	4	1	0.25	0.25	REAC:R-HSA-96652	Resistance of ERBB2	10622	1885	0.015
Regulator	0.005	4	4	1	0.25	0.25	REAC:R-HSA-96652	Drug resistance in ER	10622	735	0.015
Regulator	0.007	196	4	2	0.5	0.01	REAC:R-HSA-69275	G2/M Transition	10622	920	0.018
Regulator	0.007	198	4	2	0.5	0.01	REAC:R-HSA-45327	Mitotic G2-G2/M pha	10622	1353	0.018
Regulator	0.007	6	4	1	0.25	0.17	REAC:R-HSA-30004	Scavenging by Class	10622	1982	0.019
Regulator	0.008	217	4	2	0.5	0.01	REAC:R-HSA-89392	ESR-mediated signal	10622	764	0.020
Regulator	0.009	8	4	1	0.25	0.13	REAC:R-HSA-89371	Aryl hydrocarbon rec	10622	159	0.023
Regulator	0.010	9	4	1	0.25	0.11	REAC:R-HSA-96342	Constitutive Signaling	10622	375	0.025
Regulator	0.011	10	4	1	0.25	0.1	REAC:R-HSA-14741	Tetrahydrobiopterin	10622	2273	0.026
Regulator	0.012	###	4	3	0.75	0	REAC:R-HSA-16824	Innate Immune Syste	10622	1100	0.026
Regulator	0.012	11	4	1	0.25	0.09	REAC:R-HSA-20361	eNOS activation	10622	2462	0.026
Regulator	0.012	291	4	2	0.5	0.01	REAC:R-HSA-90069	Signaling by Nuclear	10622	2080	0.026
Regulator	0.013	13	4	1	0.25	0.08	REAC:R-HSA-56378	Signaling by EGFRvIII	10622	2023	0.027
Regulator	0.013	13	4	1	0.25	0.08	REAC:R-HSA-56378	Constitutive Signaling	10622	369	0.027
Regulator	0.014	14	4	1	0.25	0.07	REAC:R-HSA-96653	Signaling by ERBB2 E	10622	2025	0.027
Regulator	0.014	15	4	1	0.25	0.07	REAC:R-HSA-84445	The NLRP3 inflamma	10622	2277	0.027
Regulator	0.014	15	4	1	0.25	0.07	REAC:R-HSA-20213	Metabolism of nitric	10622	1306	0.027
Regulator	0.015	17	4	1	0.25	0.06	REAC:R-HSA-56378	Signaling by Ligand-	10622	2058	0.027
Regulator	0.015	17	4	1	0.25	0.06	REAC:R-HSA-12363	Constitutive Signaling	10622	370	0.027
Regulator	0.016	18	4	1	0.25	0.06	REAC:R-HSA-96656	Signaling by ERBB2 T	10622	2027	0.027
Regulator	0.016	19	4	1	0.25	0.05	REAC:R-HSA-89789	Metabolism of cofact	10622	1299	0.027
Regulator	0.017	20	4	1	0.25	0.05	REAC:R-HSA-62231	Inflammasomes	10622	1080	0.027
Regulator	0.017	21	4	1	0.25	0.05	REAC:R-HSA-96645	Signaling by ERBB2 K	10622	2026	0.027
Regulator	0.018	22	4	1	0.25	0.05	REAC:R-HSA-12279	Signaling by ERBB2 in	10622	2028	0.027
Regulator	0.018	23	4	1	0.25	0.04	REAC:R-HSA-16437	Signaling by EGFR in	10622	2022	0.027
Regulator	0.019	24	4	1	0.25	0.04	REAC:R-HSA-96608	Purinergic signaling i	10622	1674	0.027
Regulator	0.019	24	4	1	0.25	0.04	REAC:R-HSA-96644	Cell recruitment (pro	10622	304	0.027
Regulator	0.019	27	4	1	0.25	0.04	REAC:R-HSA-52189	VEGFR2 mediated va	10622	2409	0.027
Regulator	0.019	27	4	1	0.25	0.04	REAC:R-HSA-88637	Downregulation of E	10622	723	0.027
Regulator	0.019	28	4	1	0.25	0.04	REAC:R-HSA-52188	Regulated Necrosis	10622	1808	0.027
Regulator	0.019	28	4	1	0.25	0.04	REAC:R-HSA-56754	Regulation of necrop	10622	1867	0.027
Regulator	0.019	459	4	2	0.5	0	REAC:R-HSA-97165	Signaling by Rho GTI	10622	2094	0.027
Regulator	0.019	28	4	1	0.25	0.04	REAC:R-HSA-52134	RIPK1-mediated regu	10622	1726	0.027
Regulator	0.019	459	4	2	0.5	0	REAC:R-HSA-44914	Signaling by Interleu	10622	2054	0.027
Regulator	0.019	459	4	2	0.5	0	REAC:R-HSA-19431	Signaling by Rho GTI	10622	2093	0.027
Regulator	0.019	28	4	1	0.25	0.04	REAC:R-HSA-56018	PIWI-interacting RNA	10622	1540	0.027
Regulator	0.025	38	4	1	0.25	0.03	REAC:R-HSA-89505	Gene and protein exp	10622	950	0.035
Regulator	0.026	549	4	2	0.5	0	REAC:R-HSA-42247	Axon guidance	10622	182	0.035
Regulator	0.026	549	4	2	0.5	0	REAC:R-HSA-69278	Cell Cycle, Mitotic	10622	301	0.035
Regulator	0.028	574	4	2	0.5	0	REAC:R-HSA-96751	Nervous system deve	10622	1445	0.035
Regulator	0.028	44	4	1	0.25	0.02	REAC:R-HSA-96463	Aggrephagy	10622	121	0.035

Regulator	0.028	582	4	2	0.5	0	REAC:R-HSA-22627	Cellular responses to	10622	317	0.035
Regulator	0.028	47	4	1	0.25	0.02	REAC:R-HSA-90205	Interleukin-12 signal	10622	1132	0.035
Regulator	0.028	46	4	1	0.25	0.02	REAC:R-HSA-12279	Signaling by ERBB2	10622	2024	0.035
Regulator	0.032	55	4	1	0.25	0.02	REAC:R-HSA-16864	Nucleotide-binding d	10622	1485	0.040
Regulator	0.033	57	4	1	0.25	0.02	REAC:R-HSA-44711	Interleukin-12 family	10622	1131	0.040
Regulator	0.035	696	4	2	0.5	0	REAC:R-HSA-12802	Cytokine Signaling in	10622	393	0.042
Regulator	0.037	68	4	1	0.25	0.01	REAC:R-HSA-38028	Loss of proteins requ	10622	1225	0.043
Regulator	0.037	68	4	1	0.25	0.01	REAC:R-HSA-38025	Loss of Nlp from mit	10622	1222	0.043
Regulator	0.038	71	4	1	0.25	0.01	REAC:R-HSA-88545	AURKA Activation by	10622	35	0.044
Regulator	0.039	73	4	1	0.25	0.01	REAC:R-HSA-90093	Extra-nuclear estroge	10622	812	0.044
Regulator	0.041	80	4	1	0.25	0.01	REAC:R-HSA-38027	Recruitment of mitot	10622	1800	0.045
Regulator	0.041	80	4	1	0.25	0.01	REAC:R-HSA-38028	Centrosome maturat	10622	318	0.045
Regulator	0.041	80	4	1	0.25	0.01	REAC:R-HSA-96638	Selective autophagy	10622	1986	0.045
Regulator	0.043	85	4	1	0.25	0.01	REAC:R-HSA-25659	Regulation of PLK1 A	10622	1827	0.047
Regulator	0.047	94	4	1	0.25	0.01	REAC:R-HSA-44200	VEGFA-VEGFR2 Path	10622	2407	0.049
Regulator	0.047	94	4	1	0.25	0.01	REAC:R-HSA-38032	Recruitment of NuM/	10622	1799	0.049
Regulator	0.047	96	4	1	0.25	0.01	REAC:R-HSA-56209	Anchoring of the bas	10622	138	0.049
Regulator	0.047	97	4	1	0.25	0.01	REAC:R-HSA-21737	Binding and Uptake o	10622	207	0.049
Regulator	0.050	105	4	1	0.25	0.01	REAC:R-HSA-21194	Phase I - Functionaliz	10622	1589	0.050
Regulator	0.050	104	4	1	0.25	0.01	REAC:R-HSA-19413	Signaling by VEGF	10622	2101	0.050
Regulator	0.050	909	4	2	0.5	0	REAC:R-HSA-56632	Infectious disease	10622	1079	0.050
Regulator	0.005	8	2	1	0.5	0.13	REAC:R-HSA-73942	DNA Damage Revers	10622	408	0.007
Regulator	0.005	8	2	1	0.5	0.13	REAC:R-HSA-45052	HuR (ELAVL1) binds a	10622	1035	0.007
Regulator	0.005	7	2	1	0.5	0.14	REAC:R-HSA-73943	Reversal of alkylation	10622	1914	0.007
Regulator	0.040	86	2	1	0.5	0.01	REAC:R-HSA-45053	Regulation of mRNA	10622	1865	0.040
CD4+ T c	0.002	8	1	1	1	0.13	REAC:R-HSA-73942	DNA Damage Revers	10622	408	0.002
CD4+ T c	0.002	7	1	1	1	0.14	REAC:R-HSA-73943	Reversal of alkylation	10622	1914	0.002

TableS6:OS of m6A-related Cell types In the Bulk sequences						
m6A Sub	Dataset	Hazard	low	up	P.value	n
ALKBH5+CD8+ T cells-C4	GSE103479	0.887	0.574	1.37	0.591	153
B cells	GSE103479	0.54	0.273	1.07	0.077	155
CD8+ T cells	GSE103479	1.541	0.909	2.61	0.109	155
Fibroblast	GSE103479	0.483	0.175	1.33	0.161	155
HNRNPA2B1+B-C1	GSE103479	0.697	0.427	1.14	0.148	153
HNRNPA2B1+CAF-C1	GSE103479	0.74	0.533	1.03	0.071	153
HNRNPA2B1+CD8+ T cells-C1	GSE103479	0.961	0.605	1.53	0.865	153
HNRNPA2B1+mac-C3	GSE103479	0.908	0.626	1.32	0.611	153
HNRNPA2B1+Reg T cells-C1	GSE103479	1.299	0.83	2.03	0.252	153
HNRNPC+CAF-C3	GSE103479	0.698	0.467	1.04	0.079	153
Macrophage	GSE103479	0.58	0.307	1.1	0.093	155
NoneMethy+B-C4	GSE103479	0.991	0.632	1.56	0.970	153
NoneMethy-CAF-C4	GSE103479	0.909	0.601	1.37	0.650	153
WTAP+B-C2	GSE103479	0.713	0.478	1.06	0.098	153
WTAP+CAF-C2	GSE103479	0.79	0.527	1.18	0.253	153
WTAP+mac-C1	GSE103479	0.711	0.507	1	0.048	153
YTHDC1 &YTHDF3+mac_C4	GSE103479	0.718	0.468	1.1	0.129	153
YTHDF2+Reg T cells-C4	GSE103479	0.885	0.591	1.33	0.553	153
Regulatory T cells	GSE103479	0.71	0.427	1.18	0.188	155
ALKBH5+CD8+ T cells-C4	GSE17538	1.558	0.988	2.46	0.057	200
B cells	GSE17538	0.736	0.47	1.15	0.181	232
CD8+ T cells	GSE17538	0.743	0.494	1.12	0.156	232
Fibroblast	GSE17538	1.918	1.275	2.88	0.002	232
HNRNPA2B1+B-C1	GSE17538	0.693	0.459	1.05	0.081	200
HNRNPA2B1+CAF-C1	GSE17538	1.458	1.077	1.97	0.015	200
HNRNPA2B1+CD8+ T cells-C1	GSE17538	0.983	0.702	1.38	0.920	200
HNRNPA2B1+mac-C3	GSE17538	1.483	1.089	2.02	0.012	200
HNRNPA2B1+Reg T cells-C1	GSE17538	1.334	0.919	1.94	0.129	200
HNRNPC+CAF-C3	GSE17538	1.206	0.795	1.83	0.379	200
Macrophage	GSE17538	1.844	0.956	3.56	0.068	232
NoneMethy+B-C4	GSE17538	1.437	0.902	2.29	0.127	200
NoneMethy-CAF-C4	GSE17538	1.916	1.311	2.8	0.001	200
WTAP+B-C2	GSE17538	0.986	0.594	1.64	0.958	200
WTAP+CAF-C2	GSE17538	1.555	1.122	2.16	0.008	200
WTAP+mac-C1	GSE17538	1.443	1.062	1.96	0.019	200
YTHDC1 &YTHDF3+mac_C4	GSE17538	1.739	1.158	2.61	0.008	200
YTHDF2+Reg T cells-C4	GSE17538	1.071	0.694	1.65	0.758	200
Regulatory T cells	GSE17538	0.724	0.48	1.09	0.123	232
ALKBH5+CD8+ T cells-C4	GSE38832	1.013	0.584	1.76	0.964	122
B cells	GSE38832	0.396	0.179	0.88	0.022	122
CD8+ T cells	GSE38832	0.111	0.015	0.82	0.031	122
Fibroblast	GSE38832	1.707	0.771	3.78	0.187	122
HNRNPA2B1+B-C1	GSE38832	0.313	0.174	0.56	0.000	122
HNRNPA2B1+CAF-C1	GSE38832	1.417	0.905	2.22	0.128	122
HNRNPA2B1+CD8+ T cells-C1	GSE38832	0.394	0.199	0.78	0.008	122
HNRNPA2B1+mac-C3	GSE38832	0.935	0.558	1.57	0.798	122
HNRNPA2B1+Reg T cells-C1	GSE38832	0.773	0.466	1.28	0.318	122
HNRNPC+CAF-C3	GSE38832	1.019	0.508	2.04	0.957	122
Macrophage	GSE38832	0.141	0.019	1.04	0.054	122
NoneMethy+B-C4	GSE38832	0.942	0.578	1.54	0.811	122
NoneMethy-CAF-C4	GSE38832	1.766	1.022	3.05	0.042	122
WTAP+B-C2	GSE38832	0.425	0.224	0.81	0.009	122
WTAP+CAF-C2	GSE38832	1.427	0.884	2.31	0.146	122

WTAP+mac-C1	GSE38832	0.907	0.55	1.5	0.702	122
YTHDC1 &YTHDF3+mac_C4	GSE38832	0.87	0.502	1.51	0.619	122
YTHDF2+Reg T cells-C4	GSE38832	0.531	0.289	0.98	0.041	122
Regulatory T cells	GSE38832	0.48	0.225	1.03	0.058	122
ALKBH5+CD8+ T cells-C4	GSE39582	1.251	0.974	1.61	0.080	557
B cells	GSE39582	0.779	0.582	1.04	0.093	562
CD8+ T cells	GSE39582	0.783	0.589	1.04	0.091	562
Fibroblast	GSE39582	1.739	1.234	2.45	0.002	562
HNRNPA2B1+B-C1	GSE39582	0.693	0.541	0.89	0.004	557
HNRNPA2B1+CAF-C1	GSE39582	1.35	1.111	1.64	0.003	557
HNRNPA2B1+CD8+ T cells-C1	GSE39582	0.787	0.649	0.96	0.015	557
HNRNPA2B1+mac-C3	GSE39582	1.103	0.899	1.35	0.346	557
HNRNPA2B1+Reg T cells-C1	GSE39582	0.852	0.666	1.09	0.200	557
HNRNPC+CAF-C3	GSE39582	1.17	0.878	1.56	0.285	557
Macrophage	GSE39582	1.426	1.04	1.96	0.027	562
NoneMethy+B-C4	GSE39582	1.144	0.918	1.43	0.232	557
NoneMethy-CAF-C4	GSE39582	1.519	1.165	1.98	0.002	557
WTAP+B-C2	GSE39582	1.113	0.879	1.41	0.374	557
WTAP+CAF-C2	GSE39582	1.295	1.07	1.57	0.008	557
WTAP+mac-C1	GSE39582	1.049	0.876	1.26	0.604	557
YTHDC1 &YTHDF3+mac_C4	GSE39582	1.141	0.925	1.41	0.217	557
YTHDF2+Reg T cells-C4	GSE39582	1.135	0.881	1.46	0.327	557
Regulatory T cells	GSE39582	0.582	0.398	0.85	0.005	562
ALKBH5+CD8+ T cells-C4	GSE41258	1.298	0.78	2.16	0.316	152
B cells	GSE41258	0.615	0.392	0.97	0.035	152
CD8+ T cells	GSE41258	0.468	0.266	0.83	0.009	152
Fibroblast	GSE41258	2.444	1.346	4.44	0.003	152
HNRNPA2B1+B-C1	GSE41258	1.031	0.703	1.51	0.877	152
HNRNPA2B1+CAF-C1	GSE41258	1.142	0.848	1.54	0.382	152
HNRNPA2B1+CD8+ T cells-C1	GSE41258	0.74	0.526	1.04	0.085	152
HNRNPA2B1+mac-C3	GSE41258	0.901	0.607	1.34	0.604	152
HNRNPA2B1+Reg T cells-C1	GSE41258	0.772	0.51	1.17	0.222	152
HNRNPC+CAF-C3	GSE41258	0.87	0.587	1.29	0.487	152
Macrophage	GSE41258	0.691	0.422	1.13	0.143	152
NoneMethy+B-C4	GSE41258	1.246	0.928	1.67	0.143	152
NoneMethy-CAF-C4	GSE41258	1.461	0.967	2.21	0.072	152
WTAP+B-C2	GSE41258	1.283	0.898	1.83	0.171	152
WTAP+CAF-C2	GSE41258	1.344	0.932	1.94	0.114	152
WTAP+mac-C1	GSE41258	0.891	0.633	1.25	0.508	152
YTHDC1 &YTHDF3+mac_C4	GSE41258	0.8	0.53	1.21	0.287	152
YTHDF2+Reg T cells-C4	GSE41258	1.431	0.897	2.28	0.132	152
Regulatory T cells	GSE41258	0.604	0.392	0.93	0.022	152
ALKBH5+CD8+ T cells-C4	GSE72970	0.695	0.506	0.95	0.024	124
B cells	GSE72970	0.588	0.32	1.08	0.087	124
CD8+ T cells	GSE72970	0.685	0.454	1.04	0.072	124
Fibroblast	GSE72970	0.384	0.185	0.8	0.011	124
HNRNPA2B1+B-C1	GSE72970	0.852	0.584	1.24	0.407	124
HNRNPA2B1+CAF-C1	GSE72970	0.691	0.495	0.97	0.030	124
HNRNPA2B1+CD8+ T cells-C1	GSE72970	0.792	0.621	1.01	0.061	124
HNRNPA2B1+mac-C3	GSE72970	0.708	0.53	0.95	0.020	124
HNRNPA2B1+Reg T cells-C1	GSE72970	0.762	0.601	0.97	0.024	124
HNRNPC+CAF-C3	GSE72970	0.698	0.45	1.08	0.109	124
Macrophage	GSE72970	0.644	0.391	1.06	0.083	124
NoneMethy+B-C4	GSE72970	0.751	0.561	1	0.053	124
NoneMethy-CAF-C4	GSE72970	0.454	0.276	0.75	0.002	124
WTAP+B-C2	GSE72970	0.754	0.581	0.98	0.035	124
WTAP+CAF-C2	GSE72970	0.624	0.443	0.88	0.007	124
WTAP+mac-C1	GSE72970	0.671	0.474	0.95	0.025	124

YTHDC1 &YTHDF3+mac_C4	GSE72970	0.725	0.501	1.05	0.089	124
YTHDF2+Reg T cells-C4	GSE72970	0.578	0.412	0.81	0.002	124
Regulatory T cells	GSE72970	0.615	0.408	0.93	0.020	124
ALKBH5+CD8+ T cells-C4	TCGA_COAD	0.835	0.62	1.13	0.237	430
B cells	TCGA_COAD	2.014	1.172	3.46	0.011	430
CD8+ T cells	TCGA_COAD	0.608	0.404	0.92	0.018	430
HNRNPA2B1+B-C1	TCGA_COAD	0.749	0.527	1.07	0.108	430
HNRNPA2B1+CAF-C1	TCGA_COAD	1.515	1.198	1.92	0.001	430
HNRNPA2B1+CD8+ T cells-C1	TCGA_COAD	0.802	0.634	1.02	0.066	430
HNRNPA2B1+mac-C3	TCGA_COAD	1.18	0.892	1.56	0.247	430
HNRNPA2B1+Reg T cells-C1	TCGA_COAD	0.787	0.625	0.99	0.042	430
HNRNPC+CAF-C3	TCGA_COAD	1.016	0.706	1.46	0.931	430
Macrophage	TCGA_COAD	0.602	0.397	0.91	0.017	430
NoneMethy+B-C4	TCGA_COAD	0.982	0.751	1.28	0.894	430
NoneMethy-CAF-C4	TCGA_COAD	1.334	0.876	2.03	0.179	430
WTAP+B-C2	TCGA_COAD	0.762	0.544	1.07	0.114	430
WTAP+CAF-C2	TCGA_COAD	1.24	0.952	1.62	0.110	430
WTAP+mac-C1	TCGA_COAD	1.13	0.818	1.56	0.460	430
YTHDC1 &YTHDF3+mac_C4	TCGA_COAD	1.04	0.663	1.63	0.865	430
YTHDF2+Reg T cells-C4	TCGA_COAD	0.686	0.484	0.97	0.035	430
Fibroblast	TCGA_COAD	1.547	0.977	2.45	0.063	430
Regulatory T cells	TCGA_COAD	1.984	1.158	3.4	0.013	430
ALKBH5+CD8+ T cells-C4	TCGA_READ	1.048	0.582	1.89	0.875	154
B cells	TCGA_READ	0.397	0.186	0.85	0.017	154
CD8+ T cells	TCGA_READ	0.488	0.197	1.21	0.122	154
HNRNPA2B1+B-C1	TCGA_READ	0.71	0.368	1.37	0.308	154
HNRNPA2B1+CAF-C1	TCGA_READ	1.044	0.616	1.77	0.874	154
HNRNPA2B1+CD8+ T cells-C1	TCGA_READ	0.747	0.443	1.26	0.274	154
HNRNPA2B1+mac-C3	TCGA_READ	1.152	0.769	1.73	0.493	154
HNRNPA2B1+Reg T cells-C1	TCGA_READ	0.814	0.493	1.34	0.420	154
HNRNPC+CAF-C3	TCGA_READ	1.004	0.505	2	0.992	154
Macrophage	TCGA_READ	0.468	0.204	1.07	0.072	154
NoneMethy+B-C4	TCGA_READ	1.157	0.689	1.94	0.581	154
NoneMethy-CAF-C4	TCGA_READ	0.8	0.433	1.48	0.476	154
WTAP+B-C2	TCGA_READ	0.917	0.519	1.62	0.767	154
WTAP+CAF-C2	TCGA_READ	1.096	0.661	1.82	0.723	154
WTAP+mac-C1	TCGA_READ	1.251	0.811	1.93	0.312	154
YTHDC1 &YTHDF3+mac_C4	TCGA_READ	0.743	0.388	1.42	0.369	154
YTHDF2+Reg T cells-C4	TCGA_READ	1.161	0.628	2.15	0.635	154
Fibroblast	TCGA_READ	0.702	0.329	1.5	0.360	154
Regulatory T cells	TCGA_READ	0.325	0.148	0.72	0.005	154
B cells	Meta-Pooled	0.727	0.613	0.86	0.001	
CD8+ T cells	Meta-Pooled	0.721	0.614	0.85	0.001	
Fibroblast	Meta-Pooled	1.511	1.257	1.82	0.001	
HNRNPA2B1+CAF-C1	Meta-Pooled	1.197	1.079	1.33	0.001	
WTAP+CAF-C2	Meta-Pooled	1.172	1.05	1.31	0.005	
HNRNPC+CAF-C3	Meta-Pooled	0.962	0.832	1.11	0.599	
NoneMethy-CAF-C4	Meta-Pooled	1.278	1.107	1.48	0.001	
Macrophage	Meta-Pooled	0.89	0.745	1.07	0.203	
WTAP+mac-C1	Meta-Pooled	1.001	0.899	1.12	0.984	
HNRNPA2B1+mac-C3	Meta-Pooled	1.046	0.937	1.17	0.422	
YTHDC1 &YTHDF3+mac_C4	Meta-Pooled	0.999	0.876	1.14	0.983	
HNRNPA2B1+CD8+ T cells-C1	Meta-Pooled	0.797	0.717	0.89	0.001	
ALKBH5+CD8+ T cells-C4	Meta-Pooled	1.011	0.885	1.15	0.876	
HNRNPA2B1+Reg T cells-C1	Meta-Pooled	0.86	0.768	0.96	0.008	
YTHDF2+Reg T cells-C4	Meta-Pooled	0.896	0.781	1.03	0.113	
Regulatory T cells	Meta-Pooled	0.677	0.571	0.8	0.001	
HNRNPA2B1+B-C1	Meta-Pooled	0.727	0.634	0.83	0.001	

WTAP+B-C2	Meta-Pooled	0.896	0.791	1.02	0.083	
NoneMethy+B-C4	Meta-Pooled	1.049	0.934	1.18	0.421	

TableS7:RFS of m6A-related Cell types In the Bulk sequences

m6A Sub	DataSet	Hazard.Rati	low	up	P.value	n
HNRNPA2B1+B-C1	GSE103479	0.22	0.118	0.41	0.000	154
WTAP+B-C2	GSE103479	0.469	0.3	0.733	0.001	154
NoneMethy+B-C4	GSE103479	0.89	0.519	1.527	0.673	154
HNRNPA2B1+CD8+ T cells-C	GSE103479	0.96	0.551	1.673	0.886	154
ALKBH5+CD8+ T cells-C4	GSE103479	0.464	0.27	0.796	0.005	154
WTAP+mac-C1	GSE103479	0.722	0.476	1.097	0.127	154
HNRNPA2B1+mac-C3	GSE103479	1.096	0.673	1.783	0.713	154
YTHDC1 &YTHDF3+mac_C4	GSE103479	0.496	0.303	0.812	0.005	154
HNRNPA2B1+Reg T cells-C1	GSE103479	1.867	1.083	3.216	0.025	154
YTHDF2+Reg T cells-C4	GSE103479	0.694	0.415	1.159	0.163	154
HNRNPA2B1+CAF-C1	GSE103479	0.528	0.352	0.793	0.002	154
WTAP+CAF-C2	GSE103479	0.568	0.352	0.918	0.021	154
HNRNPC+CAF-C3	GSE103479	0.52	0.327	0.827	0.006	154
NoneMethy-CAF-C4	GSE103479	1.041	0.64	1.694	0.871	154
HNRNPA2B1+B-C1	GSE14333	0.685	0.496	0.947	0.022	196
WTAP+B-C2	GSE14333	0.534	0.363	0.786	0.001	196
NoneMethy+B-C4	GSE14333	0.694	0.542	0.89	0.004	196
HNRNPA2B1+CD8+ T cells-C	GSE14333	1.243	1.004	1.539	0.046	196
ALKBH5+CD8+ T cells-C4	GSE14333	1.001	0.715	1.402	0.995	196
WTAP+mac-C1	GSE14333	1.506	1.059	2.144	0.023	196
HNRNPA2B1+mac-C3	GSE14333	0.896	0.616	1.306	0.569	196
YTHDC1 &YTHDF3+mac_C4	GSE14333	0.803	0.6	1.074	0.139	196
HNRNPA2B1+Reg T cells-C1	GSE14333	0.66	0.473	0.921	0.015	196
YTHDF2+Reg T cells-C4	GSE14333	0.756	0.582	0.981	0.036	196
HNRNPA2B1+CAF-C1	GSE14333	0.773	0.628	0.95	0.015	196
WTAP+CAF-C2	GSE14333	0.822	0.664	1.018	0.072	196
HNRNPC+CAF-C3	GSE14333	0.68	0.502	0.922	0.013	196
NoneMethy-CAF-C4	GSE14333	1.185	0.907	1.549	0.214	196
HNRNPA2B1+B-C1	GSE17538	0.381	0.242	0.599	0.000	200
WTAP+B-C2	GSE17538	0.875	0.507	1.51	0.633	200
NoneMethy+B-C4	GSE17538	1.441	0.853	2.435	0.172	200
HNRNPA2B1+CD8+ T cells-C	GSE17538	0.929	0.654	1.319	0.679	200
ALKBH5+CD8+ T cells-C4	GSE17538	2.073	1.232	3.488	0.006	200
WTAP+mac-C1	GSE17538	1.741	1.22	2.486	0.002	200
HNRNPA2B1+mac-C3	GSE17538	1.719	1.211	2.439	0.002	200
YTHDC1 &YTHDF3+mac_C4	GSE17538	2.688	1.627	4.44	0.000	200
HNRNPA2B1+Reg T cells-C1	GSE17538	1.484	0.998	2.207	0.051	200
YTHDF2+Reg T cells-C4	GSE17538	0.86	0.528	1.4	0.543	200
HNRNPA2B1+CAF-C1	GSE17538	1.546	1.108	2.158	0.010	200
WTAP+CAF-C2	GSE17538	1.758	1.22	2.533	0.002	200
HNRNPC+CAF-C3	GSE17538	1.029	0.652	1.622	0.904	200
NoneMethy-CAF-C4	GSE17538	2.117	1.407	3.185	0.000	200
HNRNPA2B1+B-C1	GSE33113	0.956	0.48	1.902	0.898	96
WTAP+B-C2	GSE33113	2.959	1.623	5.394	0.000	96
NoneMethy+B-C4	GSE33113	3.191	1.728	5.892	0.000	96
HNRNPA2B1+CD8+ T cells-C	GSE33113	2.158	1.096	4.249	0.026	96
ALKBH5+CD8+ T cells-C4	GSE33113	1.733	0.79	3.802	0.170	96
WTAP+mac-C1	GSE33113	3.595	2.022	6.392	0.000	96
HNRNPA2B1+mac-C3	GSE33113	2.45	1.386	4.331	0.002	96
YTHDC1 &YTHDF3+mac_C4	GSE33113	3.162	1.666	5.999	0.000	96
HNRNPA2B1+Reg T cells-C1	GSE33113	2.588	1.391	4.815	0.003	96
YTHDF2+Reg T cells-C4	GSE33113	2.465	1.007	6.031	0.048	96
HNRNPA2B1+CAF-C1	GSE33113	2.669	1.465	4.86	0.001	96

WTAP+CAF-C2	GSE33113	4.465	2.337	8.532	0.000	96
HNRNPC+CAF-C3	GSE33113	1.222	0.607	2.459	0.574	96
NoneMethy-CAF-C4	GSE33113	4.743	2.159	10.419	0.000	96
HNRNPA2B1+B-C1	GSE37892	0.811	0.433	1.517	0.512	130
WTAP+B-C2	GSE37892	2.017	1.308	3.111	0.002	130
NoneMethy+B-C4	GSE37892	1.717	1.19	2.478	0.004	130
HNRNPA2B1+CD8+ T cells-C	GSE37892	1.501	0.994	2.266	0.053	130
ALKBH5+CD8+ T cells-C4	GSE37892	1.171	0.671	2.041	0.579	130
WTAP+mac-C1	GSE37892	1.508	0.956	2.378	0.077	130
HNRNPA2B1+mac-C3	GSE37892	1.721	1.142	2.595	0.010	130
YTHDC1 &YTHDF3+mac_C4	GSE37892	2.163	1.294	3.617	0.003	130
HNRNPA2B1+Reg T cells-C1	GSE37892	1.579	1.092	2.283	0.015	130
YTHDF2+Reg T cells-C4	GSE37892	0.99	0.541	1.81	0.973	130
HNRNPA2B1+CAF-C1	GSE37892	2.118	1.288	3.484	0.003	130
WTAP+CAF-C2	GSE37892	3.18	1.2	8.424	0.020	130
HNRNPC+CAF-C3	GSE37892	0.994	0.539	1.832	0.985	130
NoneMethy-CAF-C4	GSE37892	1.437	0.786	2.626	0.239	130
HNRNPA2B1+B-C1	GSE38832	0.314	0.185	0.534	0.000	122
WTAP+B-C2	GSE38832	0.4	0.222	0.721	0.002	122
NoneMethy+B-C4	GSE38832	0.848	0.54	1.332	0.475	122
HNRNPA2B1+CD8+ T cells-C	GSE38832	0.321	0.171	0.603	0.000	122
ALKBH5+CD8+ T cells-C4	GSE38832	0.967	0.584	1.6	0.895	122
WTAP+mac-C1	GSE38832	1.043	0.669	1.627	0.853	122
HNRNPA2B1+mac-C3	GSE38832	0.913	0.567	1.471	0.710	122
YTHDC1 &YTHDF3+mac_C4	GSE38832	0.997	0.607	1.635	0.989	122
HNRNPA2B1+Reg T cells-C1	GSE38832	0.604	0.397	0.918	0.018	122
YTHDF2+Reg T cells-C4	GSE38832	0.483	0.278	0.839	0.010	122
HNRNPA2B1+CAF-C1	GSE38832	1.447	0.964	2.174	0.075	122
WTAP+CAF-C2	GSE38832	1.498	0.969	2.316	0.069	122
HNRNPC+CAF-C3	GSE38832	0.93	0.494	1.753	0.823	122
NoneMethy-CAF-C4	GSE38832	1.857	1.131	3.049	0.014	122
HNRNPA2B1+B-C1	GSE39582	0.638	0.493	0.826	0.001	557
WTAP+B-C2	GSE39582	1.178	0.926	1.5	0.182	557
NoneMethy+B-C4	GSE39582	1.172	0.936	1.469	0.166	557
HNRNPA2B1+CD8+ T cells-C	GSE39582	0.707	0.582	0.859	0.000	557
ALKBH5+CD8+ T cells-C4	GSE39582	1.108	0.858	1.43	0.432	557
WTAP+mac-C1	GSE39582	0.989	0.822	1.19	0.907	557
HNRNPA2B1+mac-C3	GSE39582	1.015	0.823	1.251	0.890	557
YTHDC1 &YTHDF3+mac_C4	GSE39582	1.072	0.868	1.323	0.518	557
HNRNPA2B1+Reg T cells-C1	GSE39582	0.736	0.573	0.945	0.016	557
YTHDF2+Reg T cells-C4	GSE39582	1.057	0.814	1.373	0.678	557
HNRNPA2B1+CAF-C1	GSE39582	1.37	1.124	1.671	0.002	557
WTAP+CAF-C2	GSE39582	1.374	1.131	1.669	0.001	557
HNRNPC+CAF-C3	GSE39582	1.212	0.901	1.629	0.204	557
NoneMethy-CAF-C4	GSE39582	1.598	1.21	2.109	0.001	557
HNRNPA2B1+B-C1	GSE41258	2.301	1.187	4.462	0.014	152
WTAP+B-C2	GSE41258	1.783	1.027	3.094	0.040	152
NoneMethy+B-C4	GSE41258	1.849	1.145	2.987	0.012	152
HNRNPA2B1+CD8+ T cells-C	GSE41258	0.509	0.274	0.945	0.032	152
ALKBH5+CD8+ T cells-C4	GSE41258	2.177	0.916	5.173	0.078	152
WTAP+mac-C1	GSE41258	0.653	0.391	1.09	0.103	152
HNRNPA2B1+mac-C3	GSE41258	1.131	0.566	2.257	0.728	152
YTHDC1 &YTHDF3+mac_C4	GSE41258	0.983	0.459	2.104	0.965	152
HNRNPA2B1+Reg T cells-C1	GSE41258	0.987	0.468	2.081	0.972	152
YTHDF2+Reg T cells-C4	GSE41258	1.467	0.664	3.242	0.343	152
HNRNPA2B1+CAF-C1	GSE41258	1.787	1.145	2.788	0.011	152
WTAP+CAF-C2	GSE41258	1.75	1.022	2.997	0.041	152
HNRNPC+CAF-C3	GSE41258	0.807	0.408	1.598	0.539	152

NoneMethy-CAF-C4	GSE41258	2.376	1.268	4.452	0.007	152
HNRNPA2B1+B-C1	GSE72970	0.862	0.611	1.217	0.400	124
WTAP+B-C2	GSE72970	0.662	0.519	0.845	0.001	124
NoneMethy+B-C4	GSE72970	0.689	0.525	0.902	0.007	124
HNRNPA2B1+CD8+ T cells-C	GSE72970	0.73	0.582	0.916	0.007	124
ALKBH5+CD8+ T cells-C4	GSE72970	0.655	0.491	0.874	0.004	124
WTAP+mac-C1	GSE72970	0.687	0.503	0.938	0.018	124
HNRNPA2B1+mac-C3	GSE72970	0.689	0.537	0.885	0.003	124
YTHDC1 &YTHDF3+mac_C4	GSE72970	0.592	0.417	0.84	0.003	124
HNRNPA2B1+Reg T cells-C1	GSE72970	0.709	0.575	0.875	0.001	124
YTHDF2+Reg T cells-C4	GSE72970	0.574	0.421	0.783	0.000	124
HNRNPA2B1+CAF-C1	GSE72970	0.563	0.411	0.77	0.000	124
WTAP+CAF-C2	GSE72970	0.666	0.497	0.892	0.006	124
HNRNPC+CAF-C3	GSE72970	0.797	0.543	1.17	0.247	124
NoneMethy-CAF-C4	GSE72970	0.504	0.327	0.777	0.002	124
HNRNPA2B1+B-C1	TCGA_COAD	0.791	0.548	1.142	0.211	430
WTAP+B-C2	TCGA_COAD	0.591	0.422	0.828	0.002	430
NoneMethy+B-C4	TCGA_COAD	0.948	0.717	1.253	0.706	430
HNRNPA2B1+CD8+ T cells-C	TCGA_COAD	0.689	0.539	0.881	0.003	430
ALKBH5+CD8+ T cells-C4	TCGA_COAD	0.841	0.614	1.151	0.279	430
WTAP+mac-C1	TCGA_COAD	1.368	1.016	1.842	0.039	430
HNRNPA2B1+mac-C3	TCGA_COAD	1.373	1.045	1.804	0.023	430
YTHDC1 &YTHDF3+mac_C4	TCGA_COAD	1.762	1.177	2.637	0.006	430
HNRNPA2B1+Reg T cells-C1	TCGA_COAD	0.742	0.586	0.941	0.014	430
YTHDF2+Reg T cells-C4	TCGA_COAD	0.571	0.401	0.815	0.002	430
HNRNPA2B1+CAF-C1	TCGA_COAD	1.807	1.419	2.302	0.000	430
WTAP+CAF-C2	TCGA_COAD	1.562	1.204	2.025	0.001	430
HNRNPC+CAF-C3	TCGA_COAD	1.216	0.836	1.769	0.306	430
NoneMethy-CAF-C4	TCGA_COAD	1.74	1.127	2.684	0.012	430
HNRNPA2B1+B-C1	TCGA_READ	1.401	0.432	4.539	0.574	154
WTAP+B-C2	TCGA_READ	3.648	1.247	10.669	0.018	154
NoneMethy+B-C4	TCGA_READ	5.08	1.681	15.35	0.004	154
HNRNPA2B1+CD8+ T cells-C	TCGA_READ	1.103	0.436	2.791	0.836	154
ALKBH5+CD8+ T cells-C4	TCGA_READ	8.144	2.119	31.292	0.002	154
WTAP+mac-C1	TCGA_READ	1.877	0.736	4.782	0.187	154
HNRNPA2B1+mac-C3	TCGA_READ	1.626	0.683	3.871	0.272	154
YTHDC1 &YTHDF3+mac_C4	TCGA_READ	5.538	1.58	19.414	0.007	154
HNRNPA2B1+Reg T cells-C1	TCGA_READ	7.324	1.007	53.283	0.049	154
YTHDF2+Reg T cells-C4	TCGA_READ	11.636	2.966	45.648	0.000	154
HNRNPA2B1+CAF-C1	TCGA_READ	5.576	1.726	18.011	0.004	154
WTAP+CAF-C2	TCGA_READ	2.761	1.065	7.153	0.037	154
HNRNPC+CAF-C3	TCGA_READ	8.595	2.204	33.511	0.002	154
NoneMethy-CAF-C4	TCGA_READ	5.474	1.635	18.328	0.006	154
B cells	GSE17538	0.487	0.245	0.967	0.040	200
B cells	GSE33113	3.216	1.265	8.174	0.014	96
B cells	GSE37892	2.258	1.177	4.332	0.014	130
B cells	GSE38832	0.336	0.161	0.703	0.004	122
B cells	GSE39582	0.82	0.605	1.113	0.203	557
B cells	GSE41258	1.581	0.735	3.401	0.242	152
B cells	TCGA_COAD	0.569	0.373	0.869	0.009	430
B cells	TCGA_READ	7.093	0.848	59.343	0.071	154
B cells	GSE14333	0.576	0.342	0.971	0.038	196
B cells	GSE103479	0.218	0.108	0.439	0.000	154
B cells	GSE72970	0.651	0.45	0.942	0.023	124
CD8+ T cells	GSE17538	2.095	0.896	4.898	0.088	200
CD8+ T cells	GSE33113	10.869	1.45	81.471	0.020	96
CD8+ T cells	GSE37892	2.088	0.954	4.57	0.065	130
CD8+ T cells	GSE38832	0.185	0.044	0.773	0.021	122

CD8+ T cells	GSE39582	0.624	0.438	0.891	0.009	557
CD8+ T cells	GSE41258	0.64	0.243	1.685	0.367	152
CD8+ T cells	TCGA_COAD	0.57	0.375	0.868	0.009	430
CD8+ T cells	TCGA_READ	4.867	0.586	40.433	0.143	154
CD8+ T cells	GSE14333	1.411	0.956	2.081	0.083	196
CD8+ T cells	GSE103479	0.582	0.314	1.078	0.085	154
CD8+ T cells	GSE72970	0.65	0.446	0.947	0.025	124
Macrophage	GSE17538	2.921	1.379	6.19	0.005	200
Macrophage	GSE33113	5.523	1.987	15.353	0.001	96
Macrophage	GSE37892	1.965	0.927	4.167	0.078	130
Macrophage	GSE38832	0.242	0.058	1.009	0.051	122
Macrophage	GSE39582	0.745	0.547	1.016	0.063	557
Macrophage	GSE41258	0.528	0.215	1.298	0.164	152
Macrophage	TCGA_COAD	1.725	0.999	2.977	0.050	430
Macrophage	TCGA_READ	2.261	0.437	11.695	0.331	154
Macrophage	GSE14333	1.673	0.99	2.827	0.054	196
Macrophage	GSE103479	0.665	0.307	1.441	0.301	154
Macrophage	GSE72970	0.645	0.42	0.992	0.046	124
Regulatory T cells	GSE17538	1.436	0.702	2.937	0.322	200
Regulatory T cells	GSE33113	6.165	1.795	21.172	0.004	96
Regulatory T cells	GSE37892	2.105	1.07	4.14	0.031	130
Regulatory T cells	GSE38832	0.368	0.175	0.774	0.008	122
Regulatory T cells	GSE39582	0.707	0.494	1.013	0.059	557
Regulatory T cells	GSE41258	0.428	0.173	1.057	0.066	152
Regulatory T cells	TCGA_COAD	0.569	0.373	0.87	0.009	430
Regulatory T cells	TCGA_READ	9.323	1.121	77.537	0.039	154
Regulatory T cells	GSE14333	0.603	0.362	1.005	0.052	196
Regulatory T cells	GSE103479	1.579	0.848	2.942	0.150	154
Regulatory T cells	GSE72970	0.584	0.403	0.846	0.004	124
Fibroblast	GSE17538	1.853	1.083	3.17	0.024	200
Fibroblast	GSE33113	5.402	1.789	16.312	0.003	96
Fibroblast	GSE37892	5.301	1.274	22.054	0.022	130
Fibroblast	GSE38832	1.751	0.853	3.596	0.127	122
Fibroblast	GSE39582	1.853	1.317	2.606	0.000	557
Fibroblast	GSE41258	2.08	0.92	4.701	0.078	152
Fibroblast	TCGA_COAD	2.548	1.632	3.978	0.000	430
Fibroblast	TCGA_READ	6.833	0.822	56.791	0.075	154
Fibroblast	GSE14333	0.751	0.535	1.054	0.097	196
Fibroblast	GSE103479	0.594	0.317	1.113	0.104	154
Fibroblast	GSE72970	0.398	0.21	0.752	0.005	124
Fibroblast	Meta-Pooled	1.38	1.171	1.626	0.001	
HNRNPA2B1+CAF-C1	Meta-Pooled	1.184	1.076	1.303	0.001	
WTAP+CAF-C2	Meta-Pooled	1.193	1.08	1.319	0.001	
HNRNPC+CAF-C3	Meta-Pooled	0.929	0.812	1.062	0.279	
NoneMethy-CAF-C4	Meta-Pooled	1.443	1.267	1.643	0.000	
Macrophage	Meta-Pooled	1.025	0.858	1.224	0.786	
WTAP+mac-C1	Meta-Pooled	1.121	1.009	1.245	0.033	
HNRNPA2B1+mac-C3	Meta-Pooled	1.109	0.997	1.233	0.057	
YTHDC1 &YTHDF3+mac_C4	Meta-Pooled	1.09	0.967	1.229	0.158	
CD8+ T cells	Meta-Pooled	0.783	0.663	0.925	0.004	
HNRNPA2B1+CD8+ T cells-C	Meta-Pooled	0.851	0.773	0.936	0.001	
ALKBH5+CD8+ T cells-C4	Meta-Pooled	0.973	0.859	1.102	0.667	
Regulatory T cells	Meta-Pooled	0.758	0.641	0.897	0.001	
HNRNPA2B1+Reg T cells-C1	Meta-Pooled	0.863	0.778	0.957	0.005	
YTHDF2+Reg T cells-C4	Meta-Pooled	0.795	0.702	0.9	0.001	
B cells	Meta-Pooled	0.712	0.608	0.834	0.001	
HNRNPA2B1+B-C1	Meta-Pooled	0.66	0.579	0.751	0.001	
WTAP+B-C2	Meta-Pooled	0.871	0.775	0.979	0.021	

NoneMethy+B-C4	Meta-Pooled	1.03	0.927	1.144	0.587	

TableS8:m6A-related Cell type signatures to response of ICB immunotherapy in the Bulk sequences				
m6A Sub	OR	CI95	P.value	Set
Fibroblast	1.793	1.032-3.117	0.039	IMvigor210(2018)PDL1_Urothelial_Cancer
Macrophage	1.079	0.628-1.853	0.783	IMvigor210(2018)PDL1_Urothelial_Cancer
Regulatory T cells	0.889	0.517-1.526	0.67	IMvigor210(2018)PDL1_Urothelial_Cancer
CD8+ T cells	0.658	0.381-1.138	0.133	IMvigor210(2018)PDL1_Urothelial_Cancer
B cells	0.782	0.454-1.344	0.373	IMvigor210(2018)PDL1_Urothelial_Cancer
WTAP+mac-C1	1.325	0.77-2.279	0.31	IMvigor210(2018)PDL1_Urothelial_Cancer
HNRNPA2B1+mac-C3	0.895	0.521-1.537	0.688	IMvigor210(2018)PDL1_Urothelial_Cancer
YTHDC1 &YTHDF3+mac_C4	0.62	0.358-1.072	0.087	IMvigor210(2018)PDL1_Urothelial_Cancer
HNRNPA2B1+CAF-C1	0.801	0.465-1.378	0.422	IMvigor210(2018)PDL1_Urothelial_Cancer
WTAP+CAF-C2	1.957	1.123-3.406	0.017	IMvigor210(2018)PDL1_Urothelial_Cancer
HNRNPC+CAF-C3	1	0.582-1.718	1	IMvigor210(2018)PDL1_Urothelial_Cancer
NoneMethy-CAF-C4	0.815	0.474-1.399	0.459	IMvigor210(2018)PDL1_Urothelial_Cancer
HNRNPA2B1+CD8+ T cells-C1	0.381	0.215-0.674	0.001	IMvigor210(2018)PDL1_Urothelial_Cancer
ALKBH5+CD8+ T cells-C4	1.042	0.607-1.79	0.881	IMvigor210(2018)PDL1_Urothelial_Cancer
HNRNPA2B1+Reg T cells-C1	0.544	0.313-0.942	0.03	IMvigor210(2018)PDL1_Urothelial_Cancer
YTHDF2+Reg T cells-C4	1.042	0.607-1.79	0.881	IMvigor210(2018)PDL1_Urothelial_Cancer
HNRNPA2B1+B-C1	0.304	0.169-0.545	0	IMvigor210(2018)PDL1_Urothelial_Cancer
WTAP+B-C2	1.586	0.916-2.745	0.099	IMvigor210(2018)PDL1_Urothelial_Cancer
NoneMethy+B-C4	1.372	0.797-2.361	0.254	IMvigor210(2018)PDL1_Urothelial_Cancer
Fibroblast	0.658	0.224-1.933	0.447	Ulloa(2013)MAGE_A3_Melanoma
Macrophage	0.327	0.106-1.008	0.052	Ulloa(2013)MAGE_A3_Melanoma
Regulatory T cells	0.182	0.054-0.613	0.006	Ulloa(2013)MAGE_A3_Melanoma
CD8+ T cells	0.182	0.054-0.613	0.006	Ulloa(2013)MAGE_A3_Melanoma
B cells	0.415	0.135-1.273	0.124	Ulloa(2013)MAGE_A3_Melanoma
WTAP+mac-C1	0.232	0.072-0.745	0.014	Ulloa(2013)MAGE_A3_Melanoma
HNRNPA2B1+mac-C3	0.327	0.106-1.008	0.052	Ulloa(2013)MAGE_A3_Melanoma

YTHDC1 &YTHDF3+mac_C4	1.35	0.46-3.959	0.585	Ulloa(2013)MAGE_A3 _Melanoma
HNRNPA2B1+CAF-C1	0.615	0.208-1.818	0.38	Ulloa(2013)MAGE_A3 _Melanoma
WTAP+CAF-C2	0.615	0.208-1.818	0.38	Ulloa(2013)MAGE_A3 _Melanoma
HNRNPC+CAF-C3	1.067	0.364-3.13	0.906	Ulloa(2013)MAGE_A3 _Melanoma
NoneMethy-CAF-C4	0.889	0.304-2.601	0.83	Ulloa(2013)MAGE_A3 _Melanoma
HNRNPA2B1+CD8+ T cells-C1	0.289	0.093-0.897	0.032	Ulloa(2013)MAGE_A3 _Melanoma
ALKBH5+CD8+ T cells-C4	1	0.342-2.922	1	Ulloa(2013)MAGE_A3 _Melanoma
HNRNPA2B1+Reg T cells-C1	0.16	0.047-0.544	0.003	Ulloa(2013)MAGE_A3 _Melanoma
YTHDF2+Reg T cells-C4	0.889	0.304-2.601	0.83	Ulloa(2013)MAGE_A3 _Melanoma
HNRNPA2B1+B-C1	0.583	0.198-1.721	0.329	Ulloa(2013)MAGE_A3 _Melanoma
WTAP+B-C2	0.833	0.284-2.445	0.74	Ulloa(2013)MAGE_A3 _Melanoma
NoneMethy+B-C4	0.692	0.234-2.046	0.506	Ulloa(2013)MAGE_A3 _Melanoma
Fibroblast	0.744	0.354-1.563	0.436	Braun(2020)PD1_CCR CC
Macrophage	0.44	0.203-0.954	0.038	Braun(2020)PD1_CCR CC
Regulatory T cells	0.607	0.286-1.285	0.192	Braun(2020)PD1_CCR CC
CD8+ T cells	0.688	0.327-1.446	0.323	Braun(2020)PD1_CCR CC
B cells	0.73	0.347-1.534	0.406	Braun(2020)PD1_CCR CC
WTAP+mac-C1	0.523	0.245-1.119	0.095	Braun(2020)PD1_CCR CC
HNRNPA2B1+mac-C3	0.631	0.298-1.337	0.229	Braun(2020)PD1_CCR CC
YTHDC1 &YTHDF3+mac_C4	0.809	0.386-1.694	0.574	Braun(2020)PD1_CCR CC
HNRNPA2B1+CAF-C1	0.774	0.368-1.627	0.499	Braun(2020)PD1_CCR CC
WTAP+CAF-C2	0.431	0.195-0.95	0.037	Braun(2020)PD1_CCR CC
HNRNPC+CAF-C3	0.533	0.249-1.141	0.105	Braun(2020)PD1_CCR CC
NoneMethy-CAF-C4	0.825	0.394-1.728	0.61	Braun(2020)PD1_CCR CC
HNRNPA2B1+CD8+ T cells-C1	1.094	0.524-2.286	0.81	Braun(2020)PD1_CCR CC
ALKBH5+CD8+ T cells-C4	0.683	0.322-1.446	0.319	Braun(2020)PD1_CCR CC
HNRNPA2B1+Reg T cells-C1	0.858	0.41-1.797	0.685	Braun(2020)PD1_CCR CC
YTHDF2+Reg T cells-C4	1.094	0.524-2.286	0.81	Braun(2020)PD1_CCR CC
HNRNPA2B1+B-C1	1.344	0.64-2.826	0.436	Braun(2020)PD1_CCR CC

WTAP+B-C2	0.969	0.464-2.026	0.934	Braun(2020)PD1_CCRCC
NoneMethy+B-C4	1.029	0.492-2.149	0.94	Braun(2020)PD1_CCRCC
Fibroblast	0.545	0.155-1.916	0.344	Gide(2019)PD1_Melanoma
Macrophage	0.545	0.155-1.916	0.344	Gide(2019)PD1_Melanoma
Regulatory T cells	0.545	0.155-1.916	0.344	Gide(2019)PD1_Melanoma
CD8+ T cells	1.222	0.353-4.236	0.752	Gide(2019)PD1_Melanoma
B cells	0.818	0.236-2.834	0.752	Gide(2019)PD1_Melanoma
WTAP+mac-C1	0.545	0.155-1.916	0.344	Gide(2019)PD1_Melanoma
HNRNPA2B1+mac-C3	0.818	0.236-2.834	0.752	Gide(2019)PD1_Melanoma
YTHDC1 &YTHDF3+mac_C4	1.833	0.522-6.439	0.344	Gide(2019)PD1_Melanoma
HNRNPA2B1+CAF-C1	0.545	0.155-1.916	0.344	Gide(2019)PD1_Melanoma
WTAP+CAF-C2	0.545	0.155-1.916	0.344	Gide(2019)PD1_Melanoma
HNRNPC+CAF-C3	0.359	0.1-1.293	0.117	Gide(2019)PD1_Melanoma
NoneMethy-CAF-C4	0.818	0.236-2.834	0.752	Gide(2019)PD1_Melanoma
HNRNPA2B1+CD8+ T cells-C1	2.786	0.773-10.043	0.117	Gide(2019)PD1_Melanoma
ALKBH5+CD8+ T cells-C4	0.231	0.061-0.87	0.03	Gide(2019)PD1_Melanoma
HNRNPA2B1+Reg T cells-C1	1.222	0.353-4.236	0.752	Gide(2019)PD1_Melanoma
YTHDF2+Reg T cells-C4	0.818	0.236-2.834	0.752	Gide(2019)PD1_Melanoma
HNRNPA2B1+B-C1	0.545	0.155-1.916	0.344	Gide(2019)PD1_Melanoma
WTAP+B-C2	0.545	0.155-1.916	0.344	Gide(2019)PD1_Melanoma
NoneMethy+B-C4	0.429	0.096-1.924	0.269	Gide(2019)PD1+CTLA4_Melanoma
Macrophage	0.111	0.019-0.66	0.016	Gide(2019)PD1+CTLA4_Melanoma
Regulatory T cells	0.231	0.047-1.136	0.071	Gide(2019)PD1+CTLA4_Melanoma
CD8+ T cells	0.231	0.047-1.136	0.071	Gide(2019)PD1+CTLA4_Melanoma
B cells	0.429	0.096-1.924	0.269	Gide(2019)PD1+CTLA4_Melanoma
WTAP+mac-C1	0.04	0.004-0.385	0.005	Gide(2019)PD1+CTLA4_Melanoma
HNRNPA2B1+mac-C3	0.231	0.047-1.136	0.071	Gide(2019)PD1+CTLA4_Melanoma
YTHDC1 &YTHDF3+mac_C4	1.32	0.305-5.709	0.71	Gide(2019)PD1+CTLA4_Melanoma
HNRNPA2B1+CAF-C1	0.231	0.047-1.136	0.071	Gide(2019)PD1+CTLA4_Melanoma

WTAP+CAF-C2	0.04	0.004-0.385	0.005	Gide(2019)PD1+CTLA4_Melanoma
HNRNPC+CAF-C3	2.333	0.52-10.468	0.269	Gide(2019)PD1+CTLA4_Melanoma
NoneMethy-CAF-C4	4.333	0.88-21.316	0.071	Gide(2019)PD1+CTLA4_Melanoma
HNRNPA2B1+CD8+ T cells-C1	0.231	0.047-1.136	0.071	Gide(2019)PD1+CTLA4_Melanoma
ALKBH5+CD8+ T cells-C4	0.758	0.175-3.274	0.71	Gide(2019)PD1+CTLA4_Melanoma
HNRNPA2B1+Reg T cells-C1	0.758	0.175-3.274	0.71	Gide(2019)PD1+CTLA4_Melanoma
YTHDF2+Reg T cells-C4	1.32	0.305-5.709	0.71	Gide(2019)PD1+CTLA4_Melanoma
HNRNPA2B1+B-C1	0.758	0.175-3.274	0.71	Gide(2019)PD1+CTLA4_Melanoma
WTAP+B-C2	0.758	0.175-3.274	0.71	Gide(2019)PD1+CTLA4_Melanoma
NoneMethy+B-C4	0.758	0.175-3.274	0.71	Gide(2019)PD1+CTLA4_Melanoma
Fibroblast	1.322	0.444-3.938	0.616	Rose(2021)ICB_Bladder_Cancer
Macrophage	0.521	0.171-1.589	0.252	Rose(2021)ICB_Bladder_Cancer
Regulatory T cells	0.715	0.24-2.129	0.547	Rose(2021)ICB_Bladder_Cancer
CD8+ T cells	0.521	0.171-1.589	0.252	Rose(2021)ICB_Bladder_Cancer
B cells	0.715	0.24-2.129	0.547	Rose(2021)ICB_Bladder_Cancer
WTAP+mac-C1	0.972	0.329-2.875	0.959	Rose(2021)ICB_Bladder_Cancer
HNRNPA2B1+mac-C3	0.521	0.171-1.589	0.252	Rose(2021)ICB_Bladder_Cancer
YTHDC1 &YTHDF3+mac_C4	0.521	0.171-1.589	0.252	Rose(2021)ICB_Bladder_Cancer
HNRNPA2B1+CAF-C1	1.322	0.444-3.938	0.616	Rose(2021)ICB_Bladder_Cancer
WTAP+CAF-C2	1.814	0.596-5.519	0.295	Rose(2021)ICB_Bladder_Cancer
HNRNPC+CAF-C3	0.756	0.254-2.254	0.616	Rose(2021)ICB_Bladder_Cancer
NoneMethy-CAF-C4	1.399	0.47-4.169	0.547	Rose(2021)ICB_Bladder_Cancer
HNRNPA2B1+CD8+ T cells-C1	0.373	0.117-1.186	0.094	Rose(2021)ICB_Bladder_Cancer
ALKBH5+CD8+ T cells-C4	1.322	0.444-3.938	0.616	Rose(2021)ICB_Bladder_Cancer
HNRNPA2B1+Reg T cells-C1	0.373	0.117-1.186	0.094	Rose(2021)ICB_Bladder_Cancer
YTHDF2+Reg T cells-C4	0.715	0.24-2.129	0.547	Rose(2021)ICB_Bladder_Cancer
HNRNPA2B1+B-C1	0.715	0.24-2.129	0.547	Rose(2021)ICB_Bladder_Cancer
WTAP+B-C2	1.814	0.596-5.519	0.295	Rose(2021)ICB_Bladder_Cancer
NoneMethy+B-C4	1.322	0.444-3.938	0.616	Rose(2021)ICB_Bladder_Cancer

Fibroblast	7.692	1.445-40.931	0.017	Hugo(2016)PD1_Met_Melanoma
Macrophage	0.217	0.049-0.954	0.043	Hugo(2016)PD1_Met_Melanoma
Regulatory T cells	0.368	0.092-1.469	0.158	Hugo(2016)PD1_Met_Melanoma
CD8+ T cells	0.217	0.049-0.954	0.043	Hugo(2016)PD1_Met_Melanoma
B cells	4.071	0.929-17.847	0.063	Hugo(2016)PD1_Met_Melanoma
WTAP+mac-C1	0.217	0.049-0.954	0.043	Hugo(2016)PD1_Met_Melanoma
HNRNPA2B1+mac-C3	0.368	0.092-1.469	0.158	Hugo(2016)PD1_Met_Melanoma
YTHDC1 &YTHDF3+mac_C4	1.487	0.392-5.651	0.56	Hugo(2016)PD1_Met_Melanoma
HNRNPA2B1+CAF-C1	0.368	0.092-1.469	0.158	Hugo(2016)PD1_Met_Melanoma
WTAP+CAF-C2	0.368	0.092-1.469	0.158	Hugo(2016)PD1_Met_Melanoma
HNRNPC+CAF-C3	1.487	0.392-5.651	0.56	Hugo(2016)PD1_Met_Melanoma
NoneMethy-CAF-C4	0.217	0.049-0.954	0.043	Hugo(2016)PD1_Met_Melanoma
HNRNPA2B1+CD8+ T cells-C1	0.114	0.021-0.611	0.011	Hugo(2016)PD1_Met_Melanoma
ALKBH5+CD8+ T cells-C4	0.595	0.156-2.265	0.447	Hugo(2016)PD1_Met_Melanoma
HNRNPA2B1+Reg T cells-C1	0.595	0.156-2.265	0.447	Hugo(2016)PD1_Met_Melanoma
YTHDF2+Reg T cells-C4	0.368	0.092-1.469	0.158	Hugo(2016)PD1_Met_Melanoma
HNRNPA2B1+B-C1	0.595	0.156-2.265	0.447	Hugo(2016)PD1_Met_Melanoma
WTAP+B-C2	0.595	0.156-2.265	0.447	Hugo(2016)PD1_Met_Melanoma
NoneMethy+B-C4	0.368	0.092-1.469	0.158	Hugo(2016)PD1_Met_Melanoma
Fibroblast	0.875	0.176-4.338	0.87	Lauss(2017)ACT_Melanoma
Macrophage	0.214	0.038-1.205	0.08	Lauss(2017)ACT_Melanoma
Regulatory T cells	0.214	0.038-1.205	0.08	Lauss(2017)ACT_Melanoma
CD8+ T cells	0.214	0.038-1.205	0.08	Lauss(2017)ACT_Melanoma
B cells	0.444	0.087-2.274	0.33	Lauss(2017)ACT_Melanoma
WTAP+mac-C1	0.444	0.087-2.274	0.33	Lauss(2017)ACT_Melanoma
HNRNPA2B1+mac-C3	0.214	0.038-1.205	0.08	Lauss(2017)ACT_Melanoma
YTHDC1 &YTHDF3+mac_C4	0.875	0.176-4.338	0.87	Lauss(2017)ACT_Melanoma
HNRNPA2B1+CAF-C1	1.714	0.339-8.67	0.515	Lauss(2017)ACT_Melanoma
WTAP+CAF-C2	0.875	0.176-4.338	0.87	Lauss(2017)ACT_Melanoma

HNRNPC+CAF-C3	1.714	0.339-8.67	0.515	Lauss(2017)ACT_Melanoma
NoneMethy-CAF-C4	0.875	0.176-4.338	0.87	Lauss(2017)ACT_Melanoma
HNRNPA2B1+CD8+ T cells-C1	1.714	0.339-8.67	0.515	Lauss(2017)ACT_Melanoma
ALKBH5+CD8+ T cells-C4	1.714	0.339-8.67	0.515	Lauss(2017)ACT_Melanoma
HNRNPA2B1+Reg T cells-C1	3.5	0.639-19.188	0.149	Lauss(2017)ACT_Melanoma
YTHDF2+Reg T cells-C4	3.5	0.639-19.188	0.149	Lauss(2017)ACT_Melanoma
HNRNPA2B1+B-C1	8	1.213-52.695	0.031	Lauss(2017)ACT_Melanoma
WTAP+B-C2	0.214	0.038-1.205	0.08	Lauss(2017)ACT_Melanoma
NoneMethy+B-C4	0.444	0.087-2.274	0.33	Lauss(2017)ACT_Melanoma
Fibroblast	0.832	0.399-1.74	0.625	Liu(2019)PD1_Met_Melanoma
Macrophage	0.907	0.435-1.891	0.794	Liu(2019)PD1_Met_Melanoma
Regulatory T cells	0.683	0.326-1.43	0.312	Liu(2019)PD1_Met_Melanoma
CD8+ T cells	0.627	0.299-1.315	0.217	Liu(2019)PD1_Met_Melanoma
B cells	0.787	0.377-1.645	0.525	Liu(2019)PD1_Met_Melanoma
WTAP+mac-C1	0.787	0.377-1.645	0.525	Liu(2019)PD1_Met_Melanoma
HNRNPA2B1+mac-C3	0.513	0.243-1.083	0.08	Liu(2019)PD1_Met_Melanoma
YTHDC1 &YTHDF3+mac_C4	1.201	0.575-2.509	0.625	Liu(2019)PD1_Met_Melanoma
HNRNPA2B1+CAF-C1	0.958	0.459-1.998	0.91	Liu(2019)PD1_Met_Melanoma
WTAP+CAF-C2	1.201	0.575-2.509	0.625	Liu(2019)PD1_Met_Melanoma
HNRNPC+CAF-C3	0.907	0.435-1.891	0.794	Liu(2019)PD1_Met_Melanoma
NoneMethy-CAF-C4	0.683	0.326-1.43	0.312	Liu(2019)PD1_Met_Melanoma
HNRNPA2B1+CD8+ T cells-C1	0.723	0.345-1.513	0.388	Liu(2019)PD1_Met_Melanoma
ALKBH5+CD8+ T cells-C4	0.646	0.309-1.352	0.247	Liu(2019)PD1_Met_Melanoma
HNRNPA2B1+Reg T cells-C1	0.683	0.326-1.43	0.312	Liu(2019)PD1_Met_Melanoma
YTHDF2+Reg T cells-C4	1.201	0.575-2.509	0.625	Liu(2019)PD1_Met_Melanoma
HNRNPA2B1+B-C1	0.907	0.435-1.891	0.794	Liu(2019)PD1_Met_Melanoma
WTAP+B-C2	1.949	0.923-4.111	0.08	Liu(2019)PD1_Met_Melanoma
NoneMethy+B-C4	0.593	0.282-1.246	0.167	Liu(2019)PD1_Met_Melanoma
Fibroblast	0.278	0.025-3.107	0.298	Riaz(2017)PD1_Melanoma

Macrophage	0	0-Inf	0.995	Riaz(2017)PD1_Melanoma
Regulatory T cells	0.278	0.025-3.107	0.298	Riaz(2017)PD1_Melanoma
CD8+ T cells	0	0-Inf	0.995	Riaz(2017)PD1_Melanoma
B cells	0	0-Inf	0.995	Riaz(2017)PD1_Melanoma
WTAP+mac-C1	0	0-Inf	0.995	Riaz(2017)PD1_Melanoma
HNRNPA2B1+mac-C3	0.278	0.025-3.107	0.298	Riaz(2017)PD1_Melanoma
YTHDC1 &YTHDF3+mac_C4	1	0.119-8.419	1	Riaz(2017)PD1_Melanoma
HNRNPA2B1+CAF-C1	1	0.119-8.419	1	Riaz(2017)PD1_Melanoma
WTAP+CAF-C2	0	0-Inf	0.995	Riaz(2017)PD1_Melanoma
HNRNPC+CAF-C3	0.278	0.025-3.107	0.298	Riaz(2017)PD1_Melanoma
NoneMethy-CAF-C4	3.6	0.322-40.275	0.298	Riaz(2017)PD1_Melanoma
HNRNPA2B1+CD8+ T cells-C1	0.278	0.025-3.107	0.298	Riaz(2017)PD1_Melanoma
ALKBH5+CD8+ T cells-C4	1	0.119-8.419	1	Riaz(2017)PD1_Melanoma
HNRNPA2B1+Reg T cells-C1	0	0-Inf	0.995	Riaz(2017)PD1_Melanoma
YTHDF2+Reg T cells-C4	0	0-Inf	0.995	Riaz(2017)PD1_Melanoma
HNRNPA2B1+B-C1	1	0.119-8.419	1	Riaz(2017)PD1_Melanoma
WTAP+B-C2	0.278	0.025-3.107	0.298	Riaz(2017)PD1_Melanoma
NoneMethy+B-C4	0	0-Inf	0.995	Riaz(2017)PD1_Melanoma
Fibroblast	1.333	0.259-6.879	0.731	VanAllen(2016)CTLA4_Met_Melanoma
Macrophage	0.75	0.145-3.867	0.731	VanAllen(2016)CTLA4_Met_Melanoma
Regulatory T cells	1.333	0.259-6.879	0.731	VanAllen(2016)CTLA4_Met_Melanoma
CD8+ T cells	0.667	0.129-3.44	0.628	VanAllen(2016)CTLA4_Met_Melanoma
B cells	0.667	0.129-3.44	0.628	VanAllen(2016)CTLA4_Met_Melanoma
WTAP+mac-C1	0.75	0.145-3.867	0.731	VanAllen(2016)CTLA4_Met_Melanoma
HNRNPA2B1+mac-C3	0.667	0.129-3.44	0.628	VanAllen(2016)CTLA4_Met_Melanoma
YTHDC1 &YTHDF3+mac_C4	1.5	0.291-7.733	0.628	VanAllen(2016)CTLA4_Met_Melanoma
HNRNPA2B1+CAF-C1	0.667	0.129-3.44	0.628	VanAllen(2016)CTLA4_Met_Melanoma
WTAP+CAF-C2	1.333	0.259-6.879	0.731	VanAllen(2016)CTLA4_Met_Melanoma
HNRNPC+CAF-C3	1.333	0.259-6.879	0.731	VanAllen(2016)CTLA4_Met_Melanoma

NoneMethy-CAF-C4	1.5	0.291-7.733	0.628	VanAllen(2016)CTLA4_Met_Melanoma
HNRNPA2B1+CD8+ T cells-C1	0.667	0.129-3.44	0.628	VanAllen(2016)CTLA4_Met_Melanoma
ALKBH5+CD8+ T cells-C4	0.75	0.145-3.867	0.731	VanAllen(2016)CTLA4_Met_Melanoma
HNRNPA2B1+Reg T cells-C1	1.5	0.291-7.733	0.628	VanAllen(2016)CTLA4_Met_Melanoma
YTHDF2+Reg T cells-C4	0.356	0.06-2.091	0.253	VanAllen(2016)CTLA4_Met_Melanoma
HNRNPA2B1+B-C1	0.667	0.129-3.44	0.628	VanAllen(2016)CTLA4_Met_Melanoma
WTAP+B-C2	0.667	0.129-3.44	0.628	VanAllen(2016)CTLA4_Met_Melanoma
NoneMethy+B-C4	1.5	0.291-7.733	0.628	VanAllen(2016)CTLA4_Met_Melanoma
Fibroblast	1	0.104-9.62	1	JaeWon(2020)PD1_NSCLC
Macrophage	0	0-Inf	0.996	JaeWon(2020)PD1_NSCLC
Regulatory T cells	0	0-Inf	0.996	JaeWon(2020)PD1_NSCLC
CD8+ T cells	0	0-Inf	0.996	JaeWon(2020)PD1_NSCLC
B cells	0	0-Inf	0.996	JaeWon(2020)PD1_NSCLC
WTAP+mac-C1	0.238	0.019-3.014	0.268	JaeWon(2020)PD1_NSCLC
HNRNPA2B1+mac-C3	0	0-Inf	0.996	JaeWon(2020)PD1_NSCLC
YTHDC1 &YTHDF3+mac_C4	0	0-Inf	0.996	JaeWon(2020)PD1_NSCLC
HNRNPA2B1+CAF-C1	0	0-Inf	0.996	JaeWon(2020)PD1_NSCLC
WTAP+CAF-C2	0.238	0.019-3.014	0.268	JaeWon(2020)PD1_NSCLC
HNRNPC+CAF-C3	0	0-Inf	0.996	JaeWon(2020)PD1_NSCLC
NoneMethy-CAF-C4	1	0.104-9.62	1	JaeWon(2020)PD1_NSCLC
HNRNPA2B1+CD8+ T cells-C1	0.238	0.019-3.014	0.268	JaeWon(2020)PD1_NSCLC
ALKBH5+CD8+ T cells-C4	0	0-Inf	0.996	JaeWon(2020)PD1_NSCLC
HNRNPA2B1+Reg T cells-C1	0	0-Inf	0.996	JaeWon(2020)PD1_NSCLC
YTHDF2+Reg T cells-C4	1	0.104-9.62	1	JaeWon(2020)PD1_NSCLC
HNRNPA2B1+B-C1	1	0.104-9.62	1	JaeWon(2020)PD1_NSCLC
WTAP+B-C2	1	0.104-9.62	1	JaeWon(2020)PD1_NSCLC
NoneMethy+B-C4	1	0.104-9.62	1	JaeWon(2020)PD1_NSCLC
Fibroblast	2.143	0.377-12.189	0.39	Nathanson(2017)CTLA4_Melanoma
Macrophage	0.467	0.082-2.655	0.39	Nathanson(2017)CTLA4_Melanoma

Regulatory T cells	0.467	0.082-2.655	0.39	Nathanson(2017)CTLA4_Melanoma
CD8+ T cells	0.467	0.082-2.655	0.39	Nathanson(2017)CTLA4_Melanoma
B cells	0.467	0.082-2.655	0.39	Nathanson(2017)CTLA4_Melanoma
WTAP+mac-C1	0.467	0.082-2.655	0.39	Nathanson(2017)CTLA4_Melanoma
HNRNPA2B1+mac-C3	0.467	0.082-2.655	0.39	Nathanson(2017)CTLA4_Melanoma
YTHDC1 &YTHDF3+mac_C4	1	0.183-5.46	1	Nathanson(2017)CTLA4_Melanoma
HNRNPA2B1+CAF-C1	2.143	0.377-12.189	0.39	Nathanson(2017)CTLA4_Melanoma
WTAP+CAF-C2	1	0.183-5.46	1	Nathanson(2017)CTLA4_Melanoma
HNRNPC+CAF-C3	1	0.183-5.46	1	Nathanson(2017)CTLA4_Melanoma
NoneMethy-CAF-C4	1	0.183-5.46	1	Nathanson(2017)CTLA4_Melanoma
HNRNPA2B1+CD8+ T cells-C1	0.467	0.082-2.655	0.39	Nathanson(2017)CTLA4_Melanoma
ALKBH5+CD8+ T cells-C4	2.143	0.377-12.189	0.39	Nathanson(2017)CTLA4_Melanoma
HNRNPA2B1+Reg T cells-C1	1	0.183-5.46	1	Nathanson(2017)CTLA4_Melanoma
YTHDF2+Reg T cells-C4	5	0.752-33.194	0.096	Nathanson(2017)CTLA4_Melanoma
HNRNPA2B1+B-C1	1	0.183-5.46	1	Nathanson(2017)CTLA4_Melanoma
WTAP+B-C2	2.143	0.377-12.189	0.39	Nathanson(2017)CTLA4_Melanoma
NoneMethy+B-C4	2.143	0.377-12.189	0.39	Nathanson(2017)CTLA4_Melanoma
