# Supplementary material for: Single-cell N6-methyladenosine regulator patterns guide intercellular communication of tumor microenvironment that contribute to colorectal cancer progression and immunotherapy
Source: J Transl Med. 2022 May 4;20:197. doi: 10.1186/s12967-022-03395-7 (PMC9066909; doi:10.1186/s12967-022-03395-7)
Supplement: Supplementary file 2 — Additional file 2: Figure S1. The expression of m6A regulators and the association of them with ImmuneScore in CRC (Related to Fig. 1). A The expression of m6A regulators in CRC samples with main TME cell types. B The association of m6A expression with ImmuneScore in different TME cell types in scRNA-seq data. Figure S2. Exploration of cancer-association fibroblast cells in CRC. Related to Fig. 2. A t-SNE plot for stromal cells reveals different distribution of fibroblasts and non-fibroblasts between normal mucosa (2197 cells) and CRC tissue (2736 cells). B Heatmap showing the top four genes in normal mucosa and tumor fibroblasts. C The prognosis of fibroblast cells by xCell in TCGA-COAD and READ. D Trajectory analysis for cancer-association fibroblast cells. E Top genes in four NMF clusters for 3462 fibroblast cells, including m6A-fib-C1 (1939 cells, HNRNPA2B1 dominant), m6A-fib-C2 (245 cells, WTAP dominant), m6A-fib-C3 (1194 cells, HNRNPC dominant), and m6A-fib-C4 (84 cells, no m6A methylation). F Heatmap showing the average expression of cell surface protein genes in four m6A fibroblast cell clusters (Kruskal–Wallis test, p < 0.001). Figure S3. Heatmap of pseudotime Trajectory analysis for TME cell subtypes in TME main cell types of CRC, including macrophage, and B cells, as well as four types of T cells (CD8 + T, CD4 + T, Treg, and NK T cells). Figure S4. Features of m6A-mac clusters in CRC. Related to Fig. 3. A t-SNE plot of 5822 macrophages by their source class in SMC dataset. B Heatmap showing the correlation of four m6A methylation regulator clusters by NMF, named as methy-mac-C1 (n = 1432), methy-mac-C2 (n = 1538), methy-mac-C3 (n = 2169), and methy-mac-C4 (n = 442) for 5822 macrophage cells. C Heatmap showing the different expressions for m6A methylation regulators in macrophages cells. D Bar plot showing the number and percentage of methy-mac clusters, including the cluster without m6A methylation regulator expression between tumor and normal samples. E [file 12967_2022_3395_MOESM2_ESM.pdf]

Supplemental Figures for "Single-cell N^6-methyladenosine regulator patterns guide intercellular communication of tumor microenvironment that contribute to colorectal cancer progression and immunotherapy"

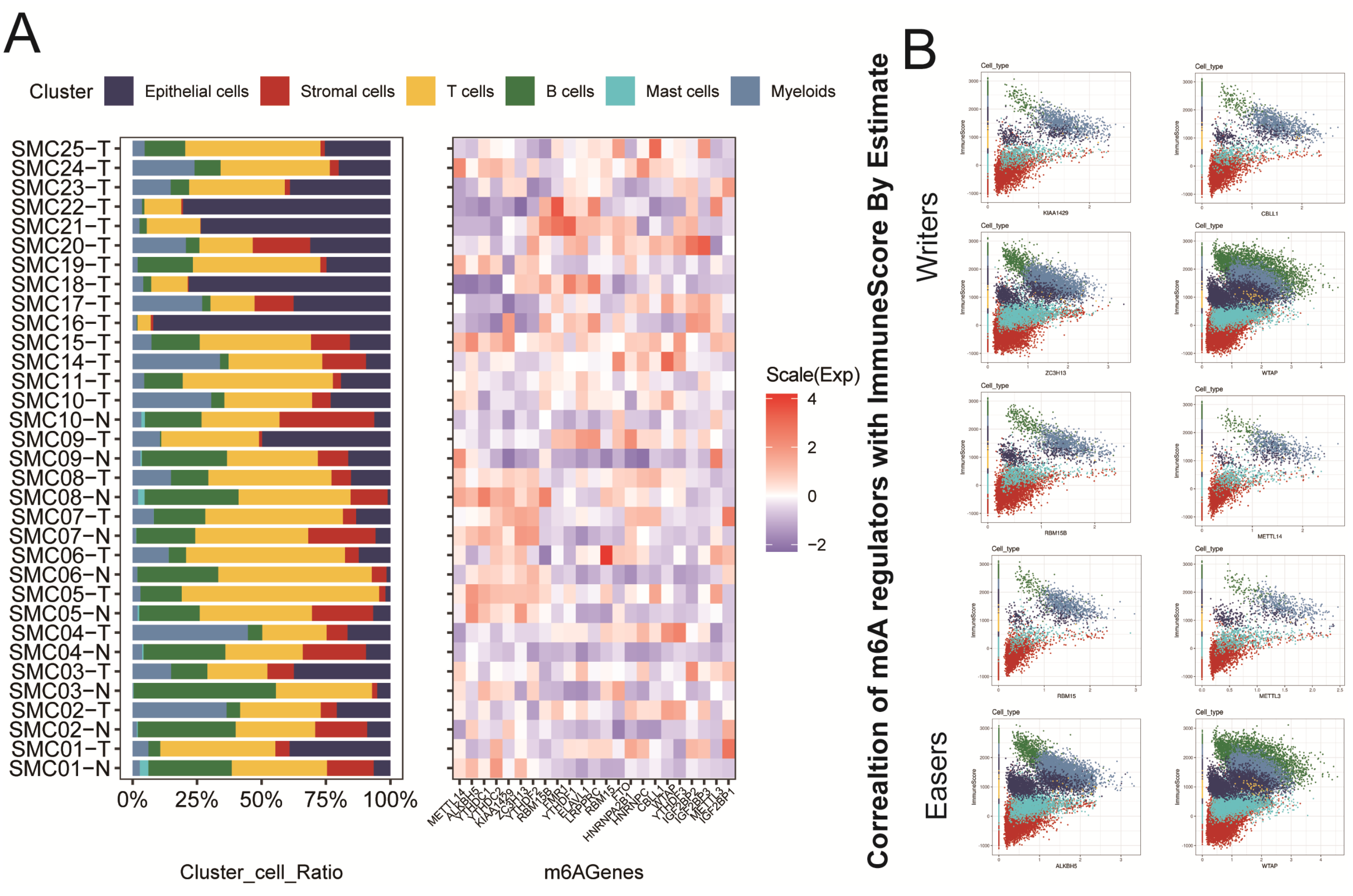

Figure S1. The expression of m^{6}A regulators and the association of them with ImmuneScore in CRC **(Related to Figure1).**

A) The expression of m^{6}A regulators in CRC samples with main TME cell types.

B) The association of m^{6}A expression with ImmuneScore in different TME cell types in scRNA-seq data.

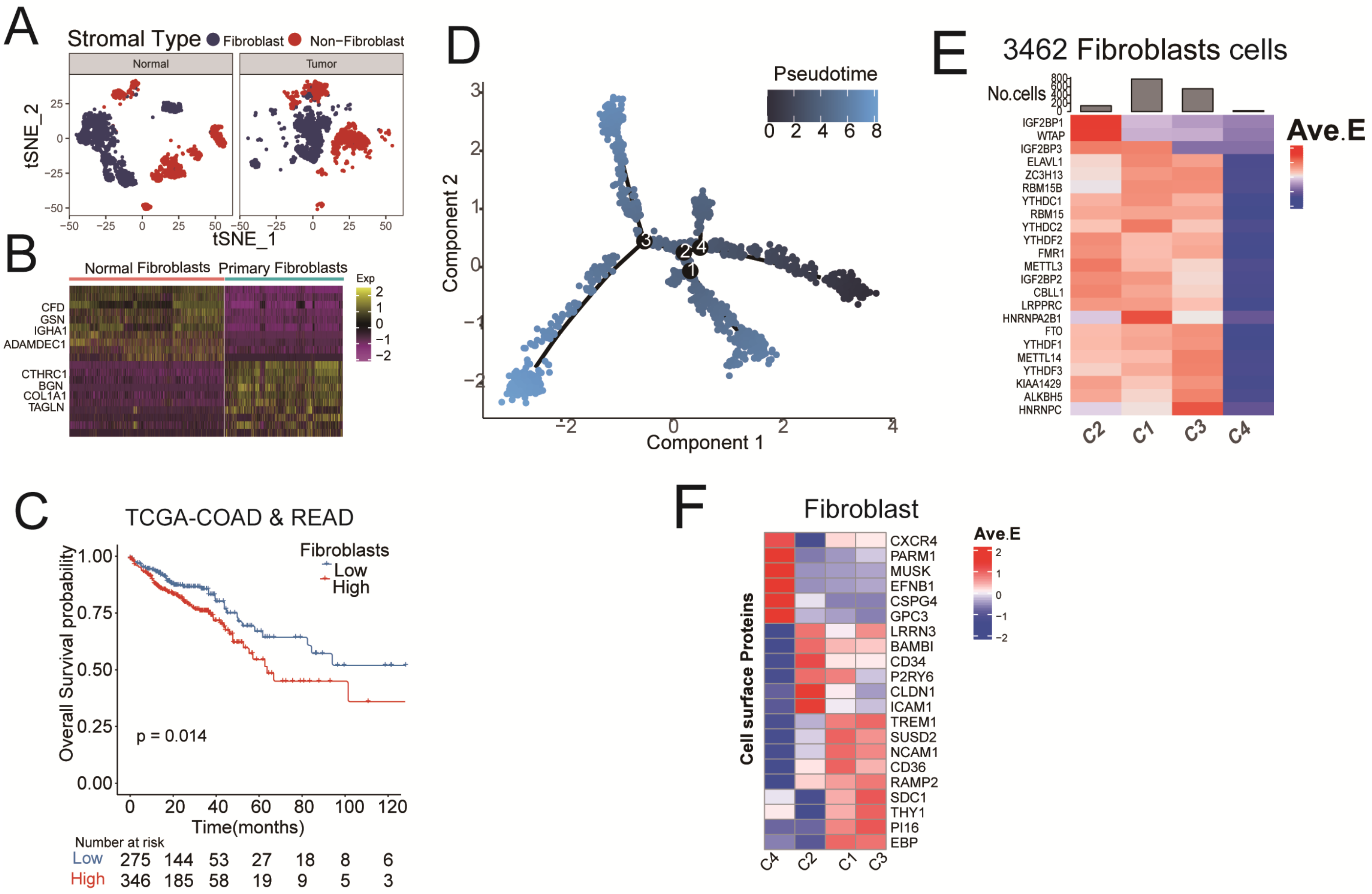

Figure S2. Exploration of cancer-association fibroblast cells in CRC. **Related to Figure2.**

(A) t-SNE plot for stromal cells reveals different distribution of fibroblasts and non-fibroblasts between normal mucosa (2197 cells) and CRC tissue (2736 cells).

(B) Heatmap showing the top four genes in normal mucosa and tumor fibroblasts.

(C) The prognosis of fibroblast cells by xCell in TCGA-COAD and READ.

(D) Trajectory analysis for cancer-association fibroblast cells.

(E) Top genes in four NMF clusters for 3462 fibroblast cells, including m^{6}A-fib-C1 (1939 cells, HNRNPA2B1 dominant), m^{6}A-fib-C2 (245 cells, WTAP dominant), m^{6}A-fib-C3 (1194 cells, HNRNPC dominant), and m^{6}A-fib-C4 (84 cells, no m^{6}A methylation).

(F) Heatmap showing the average expression of cell surface protein genes in four m^{6}A fibroblast cell clusters (Kruskal-Wallis test, $p<0.001$).

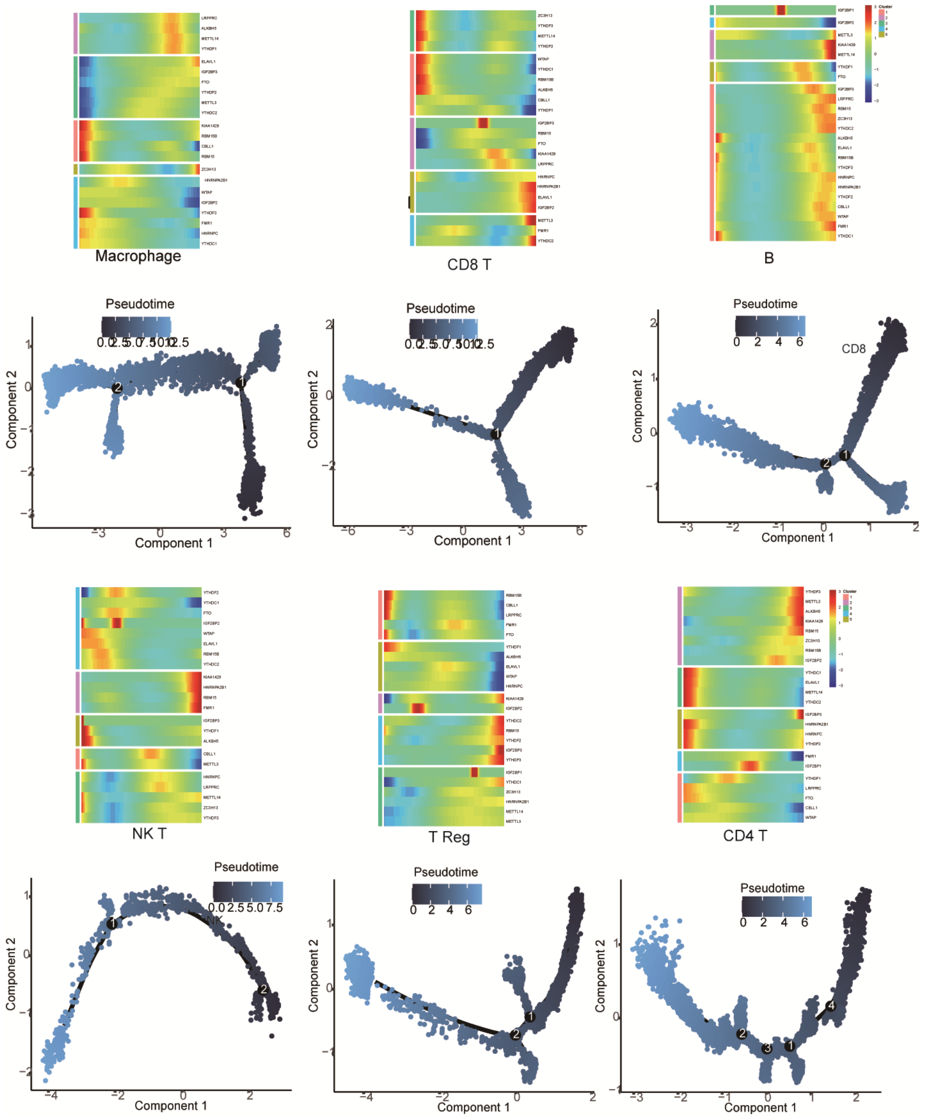

Figure S3. Heatmap of pseudotime Trajectory analysis for TME cell subtypes in TME main cell types of CRC, including macrophage, and B cells, as well as four types of T cells (CD8+ T, CD4+ T, Treg, and NK T cells).

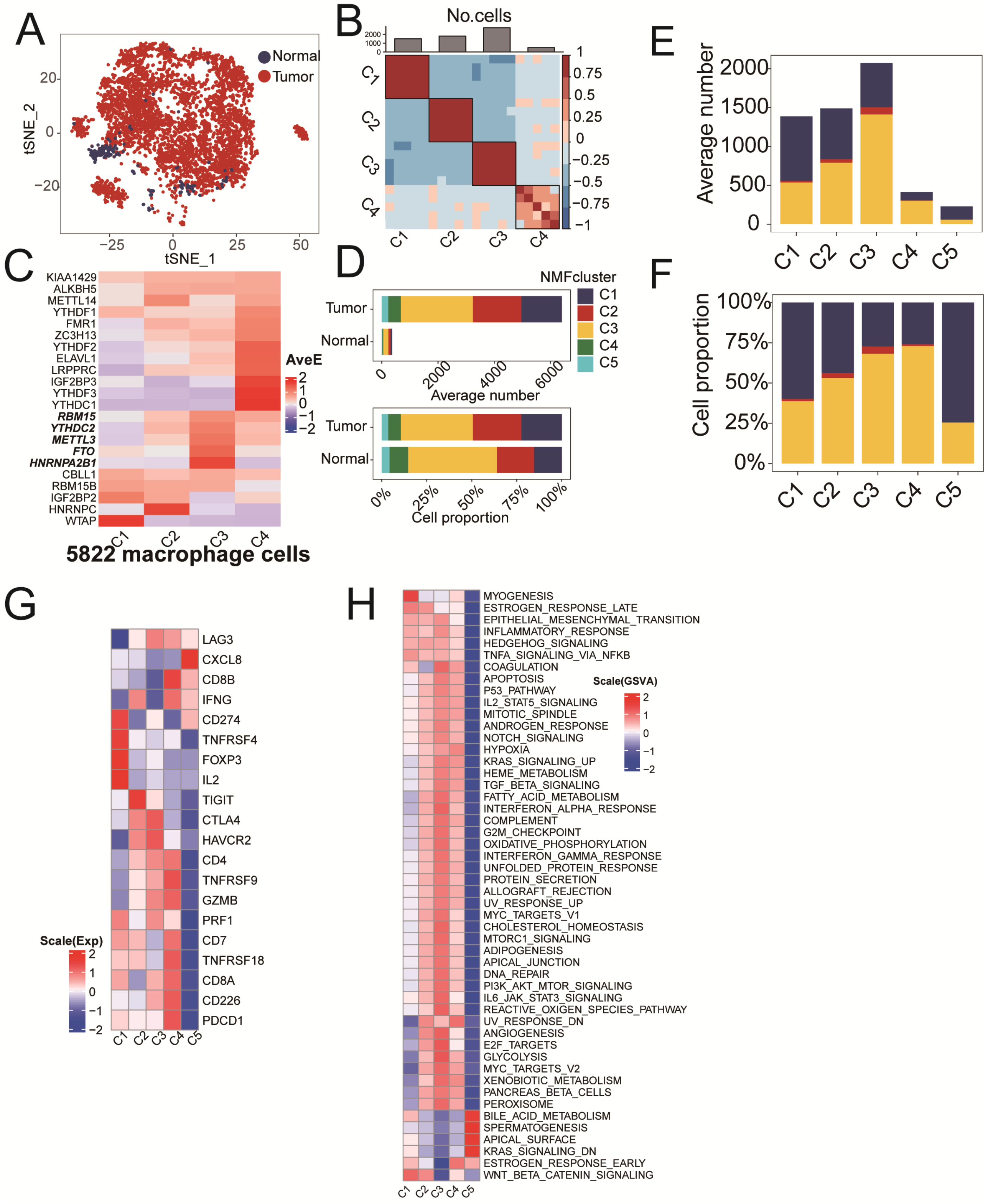

Figure S4. Features of m^{6}A-mac clusters in CRC. **Related to Figure 3.**

A) t-SNE plot of 5822 macrophages by their source class in SMC dataset.

B) Heatmap showing the correlation of four m^{6}A methylation regulator clusters by NMF, named as methy-mac-C1 (n=1432), methy-mac-C2 (n=1538), methy-mac-C3 (n=2169), and methy-mac-C4 (n=442) for 5822 macrophage cells.

(C) Heatmap showing the different expressions for m^6A methylation regulators in macrophages cells.

(D) Bar plot showing the number and percentage of methy-mac clusters, including the cluster without m^6A methylation regulator expression between tumor and normal samples.

(E) and (F) The m^6A-mac clusters were related to proinflammatory, proliferating and SPP1+ macrophage cells.

(G) The distribution of checkpoints gene expression among five m^6A-mac clusters.

(H) The activity of hallmark pathways among five m^6A-mac clusters.

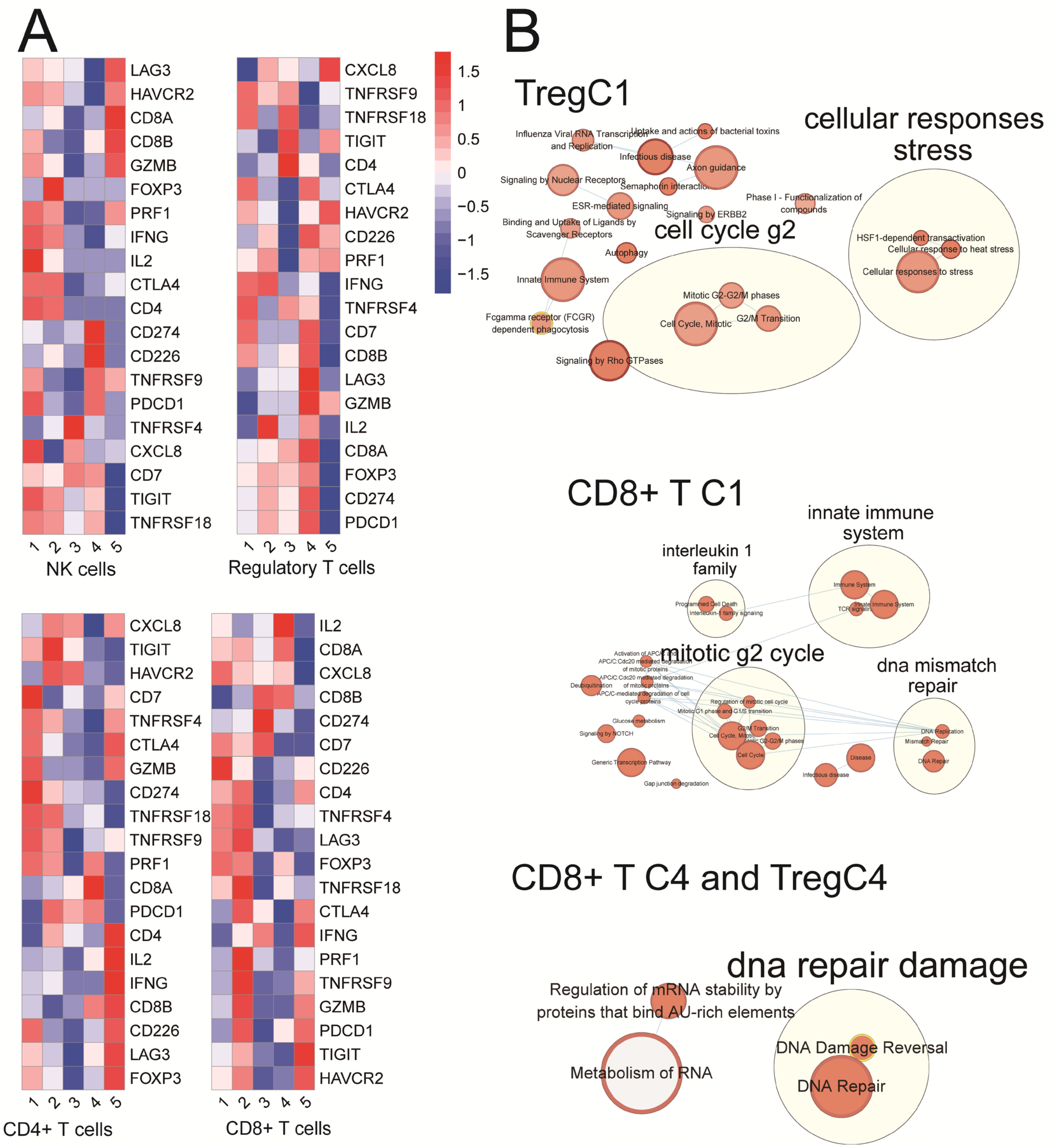

FigureS5. The immune features and prognosis of m^{6}A-T cell clusters in CRC. **Related to Figure 4.**

(A) The distribution of checkpoints gene expression among m^{6}A-related T sub cluster cells, including CD4+, Cd8+ NK, and Regulatory T cells.

(B) Enrichment cluster analysis for activated signaling ways and functions of **m6A-related macrophage types** in the Cytoscape by the REAC database.

A

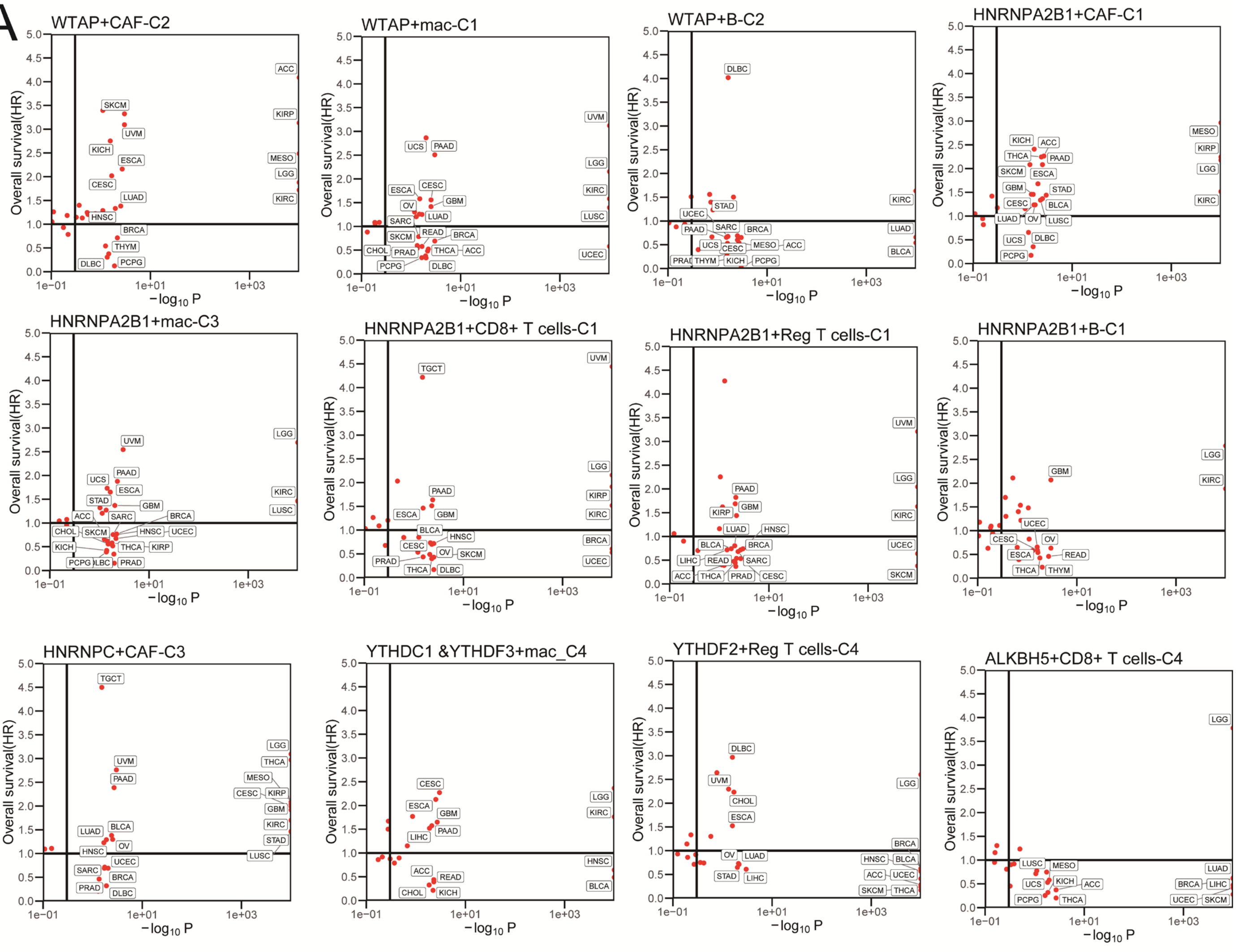

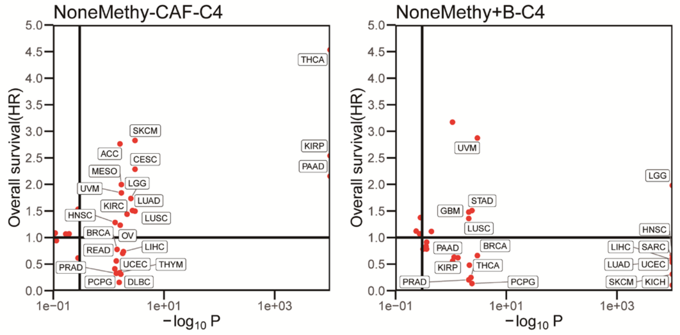

B

Prognosis of m6A related Cell clusters in PanCancer

Methy-celltype	poor	good
HNRNPA2B1+CAF-C1	17	3
WTAP+CAF-C2	16	5
HNRNPC+CAF-C3	14	8
NoneMethy-CAF-C4	12	4
WTAP+mac-C1	13	11
HNRNPA2B1+mac-C3	10	13
YTHDC1 &YTHDF3+mac_C4	7	6
HNRNPA2B1+CD8+ T cells-C1	8	10
ALKBH5+CD8+ T cells-C4	2	12
HNRNPA2B1+Reg T cells-C1	6	13
YTHDF2+Reg T cells-C4	5	11
HNRNPA2B1+B-C1	3	7
WTAP+B-C2	3	14
NoneMethy+B-C4	6	13

FigureS6. The dynamic effects to the prognosis of m6A-related subtype TME cells in the Pan Cancer patients. **Related to Figure 5.**

(A) The significant prognosis of the m6A-related cells, including CAF subtype cells (HNRNPA2B1+CAF-C1; WTAP+CAF-C2; HNRNPC+CAF-C3; NoneMethy-CAF-C4), macrophage subtype (WTAP+mac-C1;HNRNPA2B1+mac-C3;YTHDC1 &YTHDF3+mac_C4), T subtype cells(HNRNPA2B1+CD8+ T cells-C1;ALKBH5+CD8+ T cells-C4; HNRNPA2B1+Reg T cells-C1; YTHDF2+Reg T cells-C4), and B subtype cells(HNRNPA2B1+B-C1; WTAP+B-C2; NoneMethy+B-C4).

(B) Tables showed dynamic changes of the number of the prognosis among different subtypes in Pan-Cancer patients.
